# Complete Pathway Refactoring and Combinatorial Biosynthesis of Anthracyclines for Modulation of Histone Eviction and DNA Damage Bioactivities

**DOI:** 10.1002/anie.1050964

**Published:** 2026-09-23

**Authors:** Rongbin Wang, Heli Tirkkonen, Sabina Y. van der Zanden, Larissa Ponomareva, Isaline Fagon, Leslie Fève, Kendall Paige, Courtney L. Brown, Nora Schwartz, Jacob Hecht, Jennifer Nguyen, Jon S. Thorson, Jacques Neefjes, S. Eric Nybo, Khaled A. Shaaban, Mikko Metsä‐Ketelä

**Affiliations:** ^1^ Department of Life Technologies University of Turku Turku Finland; ^2^ Department of Cell and Chemical Biology (CCB) and Oncode Institute Leiden University Medical Center (LUMC) Leiden the Netherlands; ^3^ Center for Pharmaceutical Research and Innovation College of Pharmacy University of Kentucky Lexington Kentucky USA; ^4^ Department of Pharmaceutical Sciences College of Pharmacy University of Kentucky Lexington Kentucky USA; ^5^ Chemistry and Chemical Engineering Department SIGMA Clermont Aubière France; ^6^ Department of Pharmaceutical Sciences College of Pharmacy Ferris State University Big Rapids Michigan USA

**Keywords:** anthracyclines, Biobricks, natural product biosynthesis, *Streptomyces coelicolor*, synthetic biology

## Abstract

Anthracyclines including doxorubicin are cornerstone chemotherapeutic agents with high cytotoxic potency, but their use in cancer treatment is limited by irreversible dose‐dependent cardiotoxicity. Recent investigations indicate that cardiotoxicity‐free anthracyclines may be achieved through uncoupling DNA damage from chromatin damage. However, semi‐synthetic derivatization is complicated by anthracycline structural complexity. Here, we report a modular synthetic biology platform for the complete refactoring and combinatorial biosynthesis of anthracyclines in *Streptomyces coelicolor* M1152Δ*matAB*. Using standardized synthetic DNA parts, we refactored three full biosynthetic pathways of nogalamycin (38 genes), doxorubicin (31 genes), and aclacinomycin (31 genes). The pathways were organized into four functional modules (aglycone biosynthesis, TDP‐carbohydrate formation, glycosylation, and tailoring reactions) to facilitate efficient reconstruction. The modular design facilitated streamlined combinatorial biosynthesis yielding 16 derivatives, including 13 novel analogs. Bioactivity profiling across human cancer cell lines identified six potent congeners. Their cytotoxicity correlated with in vitro DNA binding affinities. Two hit molecules harbored desired bioactivity profiles promoting histone eviction at a faster rate than doxorubicin, without causing DNA double strand breaks. Our approach highlights the power of synthetic biology in rational natural product engineering and provides a scalable strategy for exploring new and improved anthracycline anti‐cancer agents.

## Graphical abstract

A synthetic biology platform was established for the complete refactoring of complex anthracycline biosynthetic pathways in *Streptomyces coelicolor* using standardized, chemically synthetic DNA parts. This modular construction enabled streamlined combinatorial biosynthesis, yielding novel analogs that decouple DNA damage from chromatin damage (histone eviction), offering a scalable strategy for searching for detoxified chemotherapeutics.

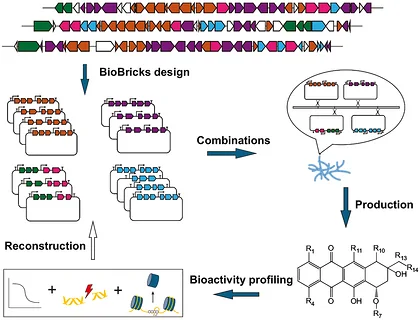

## 1 Introduction

Microorganisms are a fertile source of bioactive natural products that have provided approximately two‐thirds of antibiotics and one‐third of chemotherapeutic agents in clinical use [1]. Gram‐positive *Streptomyces* soil bacteria are a vibrant source of chemical diversity and produce natural products, such as the clinically used anti‐cancer anthracyclines doxorubicin (**1**) and aclacinomycin A (**2**) (Figure 1a) [2, 3]. Anthracyclines are effective anti‐proliferative agents that are prescribed annually to more than three million patients [4, 5]. Unfortunately, most anthracyclines, such as **1** and its semi‐synthetic derivatives cause irreversible dose‐dependent cardiotoxicity, which limits the cumulative dose that can be safely administered to patients [6]. Exceeding the recommended dose increases the risk for heart failure, and this effect limits the treatment to usually 4–6 courses [7]. Recurrent tumors cannot further be treated with these drugs. Young patients often develop cardiac problems later in life and elderly patients with existing heart conditions are excluded from doxorubicin treatment [8, 9]. Even with these precautions, cancer patients receiving anthracyclines have twice the risk of developing heart failure with an increased incidence rate correlating with the survival time of patients in remission [10]. For these reasons, the development of cardiotoxicity‐free anthracyclines would offer major benefits in cancer therapy [11]. This is illustrated by a retrospective study in which relapsed acute myeloid leukemia patients treated with the non‐cardiotoxic anthracycline **2** showed a 2‐fold improvement in five‐year overall survival [11]. Another side effect of current anthracyclines is the development of therapy induced tumors, which is the result of errors introduced in DNA during the DNA repair process following doxorubicin induced DNA breaks. Anthracycline **2** does not induce DNA breaks and does not cause these second tumors [12]. However, unlike other clinical anthracyclines, the triglycoside **2** does not penetrate solid tissues and is therefore effective only against haematological malignancies [11]. New non‐cardiotoxic and non‐genotoxic anthracyclines are needed to achieve similar benefits in relapsed solid tumors.

**FIGURE 1:**
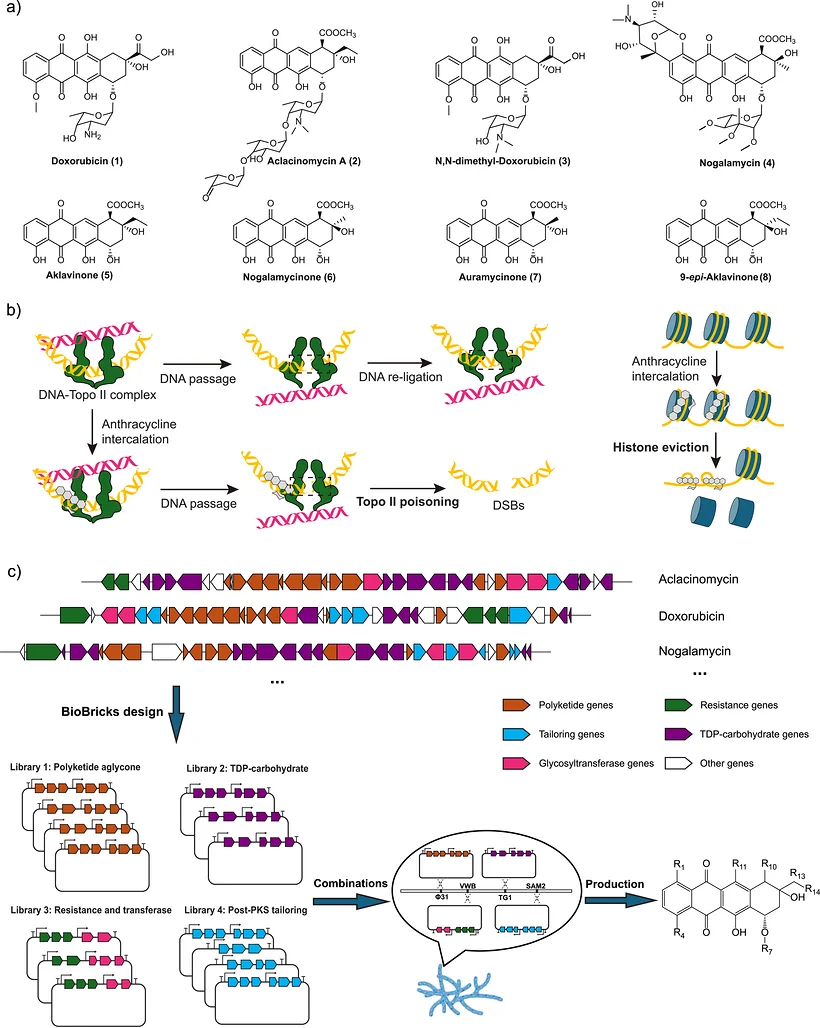
Chemical structures, mechanism of action and biosynthesis of anthracyclines. (a) Chemical structures of anthracyclines **1**–**4** and the four possible anthracyclinone aglycones **5**–**8**. (b) Models for the mechanisms of DNA damage (left) and chromatin damage (right) caused by anthracycline DNA intercalation. (c) Biosynthetic gene clusters for the aclacinomycin, doxorubicin and nogalamycin pathways, and the conceptualization of four biosynthetic modules for heterologous expression in *S. coelicolor*. Facile pathway refactoring and combinatorial biosynthesis may be achieved through introduction of different combinations of the four modules into the genomic phage integration sites (Φ31, VWB, TG1, SAM2) in the heterologous chassis. R‐groups denote functional groups amenable to modification via metabolic engineering.

The biological activities associated with anthracyclines are predominantly mediated by their ability to intercalate into DNA, which leads to inhibition of numerous cellular processes by the anthracycline‐DNA complex [13]. Evidence suggests that the cardiotoxicity of anthracyclines stems from a combination of two mechanisms: double‐stranded DNA breaks (DSBs) and chromatin damage [12, 14]. DSBs are caused by poisoning of topoisomerase II (Topo II) by the anthracyclines‐DNA complex, which blocks re‐ligation of DNA strands after the initial break (Figure 1b) [14]. In turn, chromatin damage is induced by histone eviction caused by anthracyclines competing with histones for DNA binding sites (Figure 1b), leading to epigenomic and transcriptomic changes [12]. The genomic location of DNA damage and chromatin damage are not connected [11], suggesting that these are two independent processes.

Recent investigations have uncovered that decoupling DSBs and chromatin damage bioactivities is important for the generation of low cardiotoxicity anthracyclines [12]. Selected anthracyclines, such as **2** and the experimental drug candidate *N,N*‐dimethyl‐doxorubicin (**3**) (Figure 1a) have been shown to only induce chromatin damage, but still retain high cytotoxic potency. Importantly, the clinically used **2** has been shown to lack most side effects including accumulated cardiotoxicity, but has limited bioavailability [12]. Therefore, the re‐evaluation of the bioactivities of anthracyclines could lead to the discovery of better chemotherapeutic agents, including variants that target solid tumors. However, this search has been hindered by challenges in obtaining libraries of stereochemically complex anthracyclines through organic synthesis [15]. Only few natural and semi‐synthetic anthracyclines have been characterized in detail using modern molecular techniques in vitro [12, 16] and the majority of anthracyclines discovered in the past have only been tested for their cytotoxic potency in cell lines [17].

An attractive alternative to chemical semi‐synthesis is the use of synthetic biology to program cell factories to produce desired metabolites [18, 19]. For production of anthracyclines, *Streptomyces* are optimal microbial cell factories due to an abundant pool of malonyl‐CoA and glucose‐1‐phosphate precursors, various redox cofactors, and broad antibiotic resistance [4]. Advancements in gene synthesis and genetic tools have enabled heterologous assembly of complex biosynthetic gene clusters (BGCs) in *Streptomyces* [20]. For instance, several chassis strains, such as *S. coelicolor* M1152Δ*matAB*, have been created by deleting native BGCs or nonessential genetic regions to prevent the formation of unwanted natural products [21]. Overall metabolite production and cell fitness have been improved through pleiotropic engineering [22]. Integrating and multicopy plasmids have been developed to facilitate gene transformation through intergeneric conjugation [23]. Additionally, various genetic parts, including characterized constitutive promoters, terminators, 5′ untranslated regions (5′‐UTR), and ribosome binding sites (RBS), have been screened and constructed to efficiently control *Streptomyces* gene expression [24].

The assembly of biosynthetic pathways from fully synthetic DNA is a grand challenge of metabolic engineering. Anthracyclines are type II polyketide glycosides consisting of an anthracyclinone aglycone coupled to one or more carbohydrate units [4]. The chemistry is reflected in the large size of BGCs and the biosynthesis of the classical anthracyclines **1**, **2**, and nogalamycin (**4**) (Figure 1a) require more than 30 genes (Figure 1c) [25, 26]. The characteristic tetracyclic 7,8,9,10‐tetrahydro‐5,12‐naphtacenoquinone carbon scaffold is derived from a linear decaketide that is, folded and cyclised in a controlled manner [26]. The pathways for the three natural aglycone units aklavinone (**5**), nogalamycinone (**6**), auramycinone (**7**), and the unnatural 9‐*epi*‐aklavinone (**8**) (Figure 1a) have recently been assembled using BioBricks [21]. An important factor for the bioactivity of anthracyclines is glycosylation, particularly at C7 of the aglycone, since the carbohydrate unit enhances the DNA binding activity through interactions with the minor groove of DNA [4]. The carbohydrate units are initially formed as diverse TDP‐aminosugars and TDP‐deoxysugars prior to attachment [27]. The branching TDP‐carbohydrate pathways are well suited for assembly by BioBricks, as exemplified by our recent parallel assembly of six glycosylation pathways [28]. Glycosyltransferases (GTs) are responsible for the regiospecific attachment of the carbohydrate moieties, typically at positions C1, C7, or C10 in the aglycones [29]. The final layer of complexity is generated in tailoring steps, where the anthracyclines are modified in various redox and group transfer reactions. Typical modifications include C1, C10, and C11 hydroxylations, C10 decarboxylation, and C1/C4 regioisomerization, which have recently been investigated using BioBricks [30]. The natural combinations and permutations of these gene sets during anthracycline evolution have led to great chemical diversity and the discovery of more than 500 anthracyclines to date [31, 32].

Here we have established a complete synthetic biology platform for systematic exploration of the anthracycline chemical space in *Streptomyces*. We report the first complete refactoring of three natural anthracyclines from synthetic DNA. Our modular pathway design principles allowed combinatorial biosynthesis to rapidly assemble artificial pathways leading to the generation of 13 novel anthracyclines. Subsequent evaluation of cytotoxicity, DNA binding affinity, and profiling of DNA and chromatin damage resulted in two potent anthracycline analogs with desired activity profiles. Our study provided valuable insights on the structure‐activity relationship (SAR) of anthracyclines, setting the stage for the development of improved anthracycline anti‐cancer drugs in the future. This work provides an example of rapid large‐scale systematic exploration to generate novel chemistry by functional gene combinations and highlights the utility of BioBricks in the parallel assembly of highly branching biosynthetic pathways.

## 2 Results and Discussion

### 2.1 Design Philosophy and Establishment of the Platform

We selected BioBricks assembly for construction of anthracycline BGCs, since our goal was to assemble several branching pathways in parallel to produce new anthracycline variants. BioBricks is based on recycling of protected restriction enzyme cleavage sites that allows facile addition of any single genes to any construct from a reusable synthetic DNA library [21]. The method excels in stepwise pathway assembly, and merging genetic material from several related gene clusters in various combinations and permutations. To achieve a bottom‐up synthetic biology platform, we sourced synthetic DNA according to the BioBricks [RFC‐10] standard and built all the necessary genetic tools, including promoters, terminators, RBS, and biosynthetic genes (Tables S1, S2) [21]. Furthermore, BioBricks design is tailored to overcome the difficulties associated with multi‐gene assembly techniques of high GC content DNA [33] and low recombination efficiency in *Streptomyces* [34].

*S. coelicolor* M1152∆*matAB* was selected as the chassis for heterologous expression due to (i) minimal genetic background, (ii) lack of endogenous gene clusters, (iii) enhanced secondary metabolite production, and (iv) improved biomass accumulation [21, 22]. We designed five BioBricks compatible plasmids, which could be transformed by intergeneric conjugation and integrated to five actinophage sites in the genome of *S. coelicolor* M1152∆*matAB* (Table S1) [35, 36]. The complex anthracycline pathways were refactored into four modules (Figure 1c), which included functionalities for (i) aglycone biosynthesis (burnt orange), (ii) TDP‐carbohydrate biosynthesis (purple), (iii) self‐resistance (green) and carbohydrate transfer (pink), and (iv) tailoring steps (light blue).

To initiate the study, we overcame several engineering challenges to streamline anthracycline pathway refactoring. First, we sought to improve the production titer of **5**, a key synthon for **1**, **2**, and **3** (Figure 2a). We refactored plasmid pEN10001 to pAKV7 by changing promoters, gene order and inclusion of terminators sequences to improve the titer one‐fold to 5 mg/L. Additional incorporation of LXA‐1180 resin into the fermentation resulted in another four‐fold improvement to 20 mg/L (Figure 2a).

**FIGURE 2:**
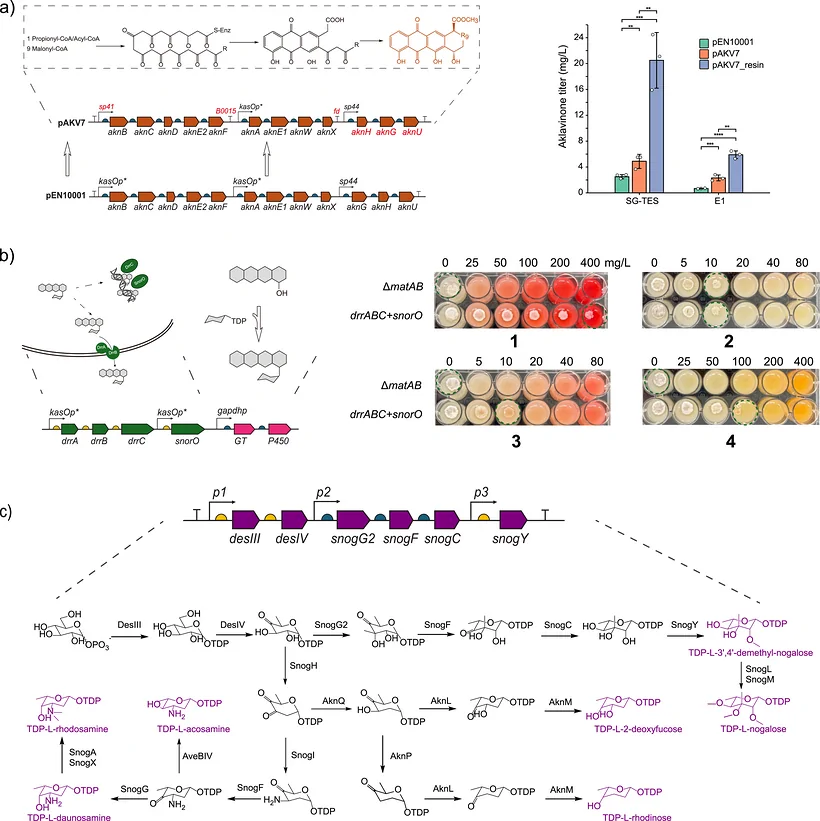
Design, assembly, and verification of BioBricks modules. (a) Optimization of aklavinone production. The stronger promoter *sp41* was utilized to enhance the transcription of *aknBCDE2F*. Terminators B0015 and *fd* were inserted to insulate and stabilize the transcripts of operons. The fourth ring cyclase gene *aknH* was set next to the promoter to enhance transcription. The statistical difference of the product yields in two culture media (SG‐TES and E1) displayed in the bar chart was conducted by one‐way ANOVA with multiple comparisons, **p < 0.01, ***p < 0.001. (b) Construction and functionality of resistance and GT modules. Left panel, schematic illustration of enzyme mechanisms and gene cassette design. Right panel, microbial inhibition assay verifying resistance gene functionality. The engineered strain carrying *drrABC* and *snorO* genes was compared with its parental strain M1152Δ*matAB* on ISP4 agar plates supplemented with increasing concentrations of anthracyclines **1–4**. Green dashed circles mark the highest tolerable concentrations. (c) Pathways and gene cassettes for biosynthesis of various TDP‐deoxysugars. The SBOL diagram presents the gene cassette of TDP‐l‐3′,4′‐demethyl‐nogalose as an example. Yellow semi‐circles in SBOL diagrams in Figure 2 represent synthetic RBS BBa_B0034 (5′‐AAAGAGGAGAAA‐3′), while those in cyan are native RBS of the genes.

Second, we developed a drug resistance and glycosylation module. These functionalities were merged, as anthracyclines typically acquire biological activity after glycosylation and therefore providing self‐resistance at this step was deemed necessary [4]. To confer self‐defence against anthracycline antibiotics, we utilized an ATP‐binding cassette transporter (*drrAB*) and two UvrA‐like excinucleases (*drrC, snorO*) for improved DNA repair (Figure 2a) [37, 38, 39]. A microbial inhibition assay confirmed that *S. coelicolor* M1152∆*matAB* was susceptible to **1**, **2**, **3,** and **4**, whereas the strain transformed with the resistance genes exhibited increased resistance to **1**, **3,** and **4** (Figure 2b). Of note, the resistance to the triglycosylated **3** was not improved, indicating that the substrate scope of the *drrAB* transporter may not extend beyond monoglycosylated anthracyclines. This result nonetheless gave confidence that the selected resistance genes could provide sufficient self‐defence and drug efflux against various anthracycline analogs. To incorporate the carbohydrate transfer functionality, genes encoding glycosyltransferase (GT), and auxiliary CYP450 genes were included in the module (Figure 2b). We assembled cassettes for glycosylation of **1** (*dnrQS*) [40], **2** (*aknSTK*) [41, 42], and **4** (*snogEND*) [25, 43] and assessed the functionality by complementation experiments (Figures 2b, S1).

Third, we cloned the gene sets for neutral TDP‐deoxysugars, such as TDP‐l‐nogalose (*snogG2FCYLM*) [25] from the nogalamycin pathway, TDP‐2‐deoxy‐l‐fucose (*aknLMQ*) [41], and TDP‐l‐rhodinose (addition of *aknP*) [44] from the aclacinomycin pathway (Figure 2c). We also assembled gene cassettes for TDP‐aminosugars (Figure 2c), including the core construct for TDP‐l‐daunosamine (*desIII*, *desIV*, *snogHIFG*) [45, 46]. TDP‐l‐rhodosamine was obtained by inclusion of the *O*‐methyltransferases (*snogA, snogX*) [46], while production of TDP‐l‐acosamine was achieved by changing the 4’‐ketoreductase (*snogG* to *aveBIV* from the avermectin pathway) [47]. These constructs were functionally tested via exhaustive feeding and genetic complementation studies to ensure their use for total biosynthesis (Figure S1). Of note, this is the first time that the TDP‐l‐nogalose, TDP‐2‐deoxy‐l‐fucose, and TDP‐l‐rhodinose pathways have been experimentally verified in vivo, thus completing our understanding of the last remaining steps in the biosynthesis of **2** and **4**.

### 2.2 Complete Refactoring of Natural Anthracycline Pathways

After completion of the individual modules, we focused on the total biosynthesis of the nogalamycin, doxorubicin, and aclacinomycin pathways. To accomplish the complete refactoring of the nogalamycin pathway, we assembled 38 genes from the native pathway or utilized homologs from related secondary metabolism pathways (Figure 3a). The carbon scaffold **6** was provided by the nogalamycinone plasmid pRW10000 [21]. Introduction of the resistance, glycosyltransferase and TDP‐carbohydrate modules led to the production of l‐3’,4’‐demethyl‐nogalose‐nogalamycinone (**11**) in RW‐30 strain (Figure 3a), which was confirmed by comparison to an authentic standard and by MS analysis (Figures S2, S3). The production profile reflected substantive metabolic flux to **11**, indicating balanced expression and robust attachment of the first carbohydrate at C7. The introduced modules also included genes for the biosynthesis and transfer of the rhodosamine carbohydrate, but the second glycosylation event did not occur in the strain due to lack of genes for 1‐hydroxylation. Introduction of the fourth tailoring module for 1‐hydroxylation (*snoaWL2*) [48] and 2″‐hydroxylation (*snoT*), epoxyoxocin ring formation (*snoK*), 4″‐epimerization (*snoN*) and *O‐*methylations (*snogLM*) resulted in the total biosynthesis of **4** in RW‐48 strain (Figure 3a) [49, 50]. The clean product yield of 33.6 mg/L of **4** in E1 medium was confirmed by comparison to an authentic standard in UHPLC analysis and by LC‐ESI(‐)‐HRMS (Figure S4, calculated: 786.2973 [M‐H]^−^; found: 786.2990 [M‐H]^−^).

**FIGURE 3:**
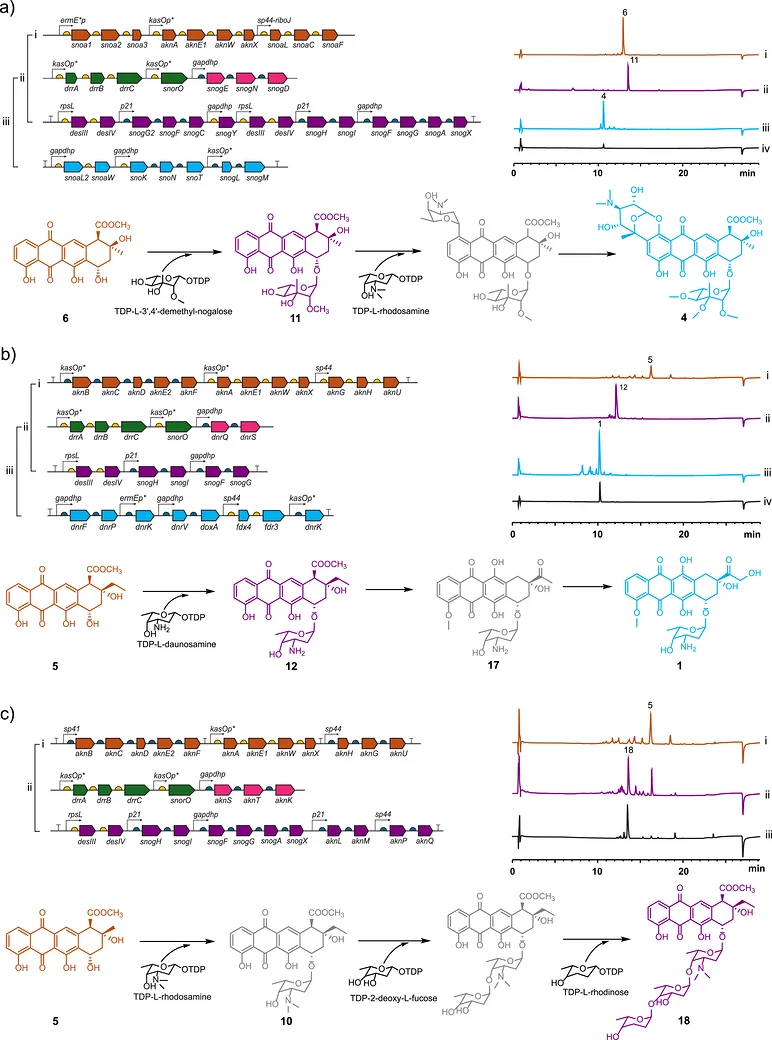
Complete refactoring of natural anthracycline pathways. The biosynthetic genes in the SBOL diagrams, HPLC chromatogram traces, and compound structures in pathway schemes are color‐coded for consistency. Roman numerals in the SBOL diagrams and HPLC chromatogram traces are used to link the plasmid combinations to the metabolite production profiles. Authentic standards are shown in black and structures of intermediates in pathway schemes are shown in grey. (a) Nogalamycin pathway refactoring. (i) Production profile of strain NOG at 430 nm; (ii) Production profile of strain RW‐30 at 430 nm; (iii) Production profile of strain RW‐48 at 470 nm; (iv) Authentic standard of **4** at 470 nm. (b) Doxorubicin pathway refactoring. (i) Production profile of strain AKV1 at 430 nm; (ii) Production profile of strain RW‐17 at 430 nm; (iii) Production profile of strain RW‐38 at 490 nm; (iv) Authentic standard **1** at 490 nm. (c) Aclacinomycin pathway refactoring. (i) Production profile of strain AKV7 at 430 nm; (ii) Production profile of strain RW‐21 at 430 nm; (iii) Authentic standard **18** at 430 nm.

For the 31 gene doxorubicin pathway, the aklavinone producing strain M1152∆*matAB*::pEN10001 (AKV1) was chosen as the initial chassis [21]. Transformation of the expression cassettes for the glycosyltransferases and TDP‐l‐daunosamine resulted in the appearance of 11‐deoxy‐rhodomycin D (**12**) as the main product (Figure 3b), which was identified by comparison to an authentic standard and MS analysis (Figures S3, S5). However, addition of the doxorubicin tailoring module revealed 4‐*O*‐methylation (*dnrK*) [51] as a bottleneck step and necessitated step‐wise development cycles (Figure S6a). Introduction of the 11‐hydroxylation (*dnrF*) [52] and 15‐demethylation (*dnrP*) [53] activities led to full conversion of the substrate **12** to 15‐demethoxy‐rhodomycin D (**14**) (Figures S6b, S7), but only 18.7% was converted further to 13‐deoxydaunomycin (**15**) by *dnrK* (Figures S6c, S8). The P450 mono‐oxygenase (*doxA*) and the auxiliary gene (*dnrV*) catalyze three consecutive late‐stage oxidation steps to generate 13‐dihydrodaunorubicin (**16**), daunorubicin (**17**), and the final product **1**. Conservation of the natural operon structure for *doxA* and *dnrV* was important for formation of **1** (Figures S6d, S9‐S10) [54] and the activity of the P450 enzyme was further improved by addition of the natural native ferredoxin (*fdx4*) and ferredoxin reductase (*fdr3*) for transferring electrons to DoxA (Figure S6e) [55]. To improve flux to **1**, we included another copy of *dnrK* with the strong promoter kasOp*, which resulted in a clean production profile of **1** in strain RW‐42 with a yield of 1.5 mg/L (Figures 3b and S6f).

Refactoring of the 31 gene pathway for the triglycosylated aclacinomycin N (**18**) was facilitated by our expanding BioBricks construct library. We utilized the improved strain AKV7 for initial production of aklavinone (Figure 3c). The resistance cassette was enriched with the native aclacinomycin glycosyltransferase (*aknST*) [41] for the first sugar unit TDP‐l‐rhodosamine and the second bifunctional glycosyltransferase (*aknK*) [42], which appends both TDP‐2‐deoxy‐l‐fucose and TDP‐l‐rhodinose. For biosynthesis of the required TDP‐carbohydrates, we resourced the existing TDP‐l‐rhodosamine cassette from the nogalamycin pathway and included four genes (*aknLMPQ*) to achieve formation of the two neutral deoxysugars (Figures 2c and 3c). The production strain yielded 2.4 mg/L of **18** in strain RW‐21, which was identified based on comparison to an authentic standard (Figure 3c) and by HRMS analysis (calculated: 814.3645 [M+H]^+^; found: 814.3639 [M+H]^+^, Figure S11). Accumulation of the intermediate **5** was also detected from the strain (Figure 3c), which highlighted the challenges in balancing expression for aglycone production, tandemly‐acting glycosyltransferases, and multiple TDP‐sugar donors.

### 2.3 Combinatorial Biosynthesis for Novel Anthracycline Analogs

Anthracyclines share similar core structures, but exhibit great structural diversity when the sugar units and multifaceted tailoring modifications are considered [4, 27]. Our BioBricks platform offered straightforward opportunities to increase the structural diversity of anthracyclines by combining genetic elements from different biosynthetic pathways using combinatorial biosynthesis [56]. We rationalized that our modular pathway design would provide access to unexplored anthracycline chemical space using rare non‐aklavinone aglycones in combination with atypical aminosugar substituents that have been shown to impact histone eviction and double strand break activities [12, 14, 57].

We estimated that the key factor for successful combinatorial biosynthesis of anthracyclines depends on the promiscuity of the glycosyltransferases. The anthracycline 7‐*O*‐glycosyltransferases possess a GT‐B fold with an *N‐*terminal domain that binds the aglycone acceptor and a *C‐*terminal domain that binds the sugar nucleotide donor [58]. In order to probe the substrate tolerance, we first utilized the daunorubicin glycosyltransferase (*dnrQS*) (Figure 4a) to combine the natural TDP‐l‐daunosamine to non‐cognate aglycones **7**, **8,** and **6** resulting in **19**, **20,** and **21**, respectively (Figure 4b). Next, we transferred TDP‐l‐acosamine, which is the 4’‐epimer TDP‐l‐daunosamine in the clinically relevant epirubicin [4], to **8** and **6** to generate **22** and **23** (Figure 4b), respectively. We switched to the aclacinomycin glycosyltransferase (*aknST*) (Figure 4a) to be able to utilize TDP‐l‐rhodosamine, which could be transferred to all three unnatural aglycones **7**, **8,** and **6** resulting in **24**, **25,** and **26** (Figure 4b), respectively. The use of neutral deoxysugars, such as l‐3’,4’‐demethyl‐nogalose was enabled with the nogalamycin glycosyltransferase (*snogEN*) (Figure 4a), which we used to transfer the carbohydrate unit to the non‐cognate C21 carbon aglycones **8** and **5** to generate **27** and **28** (Figure 4b), respectively.

**FIGURE 4:**
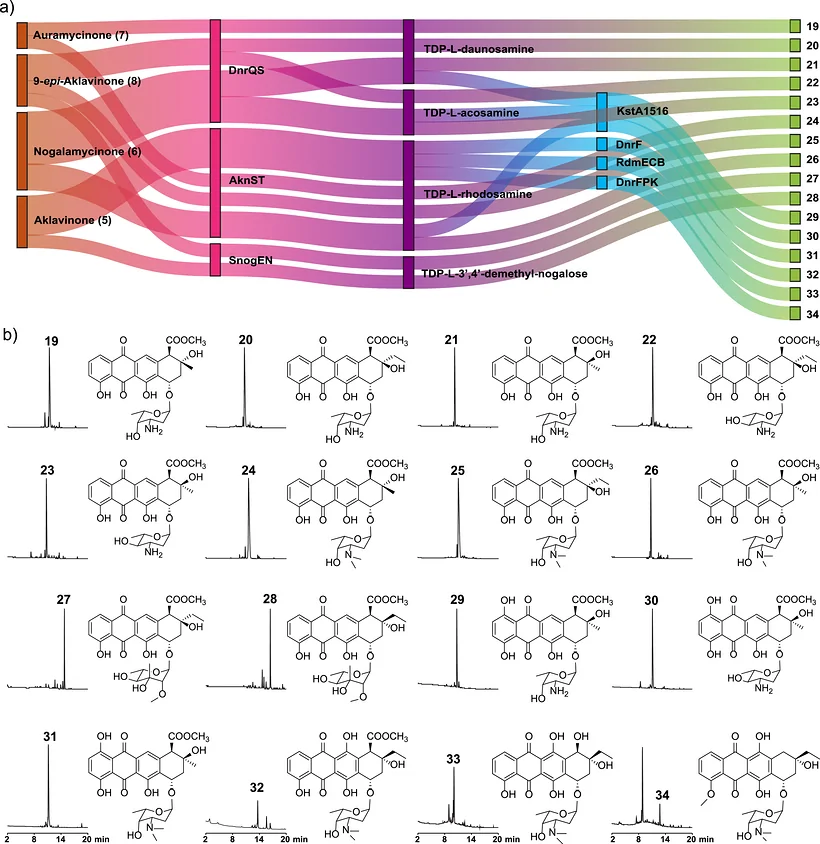
Combinatorial biosynthesis for novel anthracyclines. (a) Pathline graph depictions of various module combinations resulting in the production of novel anthracyclines. The module combinations for aglycones (burnt orange), GTs (pink), TDP‐carbohydrates (purple), and tailoring (light blue) are shown in columns, while the generated compounds **19**–**34** are shown in rows. For clarity, aglycone and TDP‐carbohydrate modules are denoted based on compound structure, while GT and tailoring modules are depicted using gene names. (b) HPLC traces showing the production profiles and their structures. Traces for **19–28** were recorded at 430 nm, while traces for **29–34** were recorded at 490 nm.

Next, we enriched selected strains with additional tailoring functionalities to further increase the chemical diversity. We recruited C1 hydroxylation activity from the kosinostatin pathway (*kstA15, kstA16*) [30, 59] (Figure 4a) and incorporated the genes into three nogalamycinone‐based strains, resulting in the new congeners, **29**, **30**, and **31** (Figure 4b). We utilized strain RW‐19 producing **10** as a host to further demonstrate regiospecific modifications. Addition of C11 hydroxylation (*dnrF*) activity resulted in the production of the known anthracycline rhodomycin T (**32**) (Figure 4b). Biosynthetic material from the rhodomycin pathway was used for C10 demethylation (*rdmC*), C10 hydroxylation (*rdmB*), and C11 hydroxylation (*rdmE*) (Figure 4a), which resulted in the formation of rhodomycin B (**33**) [60] (Figure 4b). In order to generate **3**, we introduced the tailoring genes from the doxorubicin pathway (*dnrFPKV+doxA+fdx4+fdr3*) to the aclacinomycin T producing strain. However, only the penultimate intermediate **34** was detected (Figure 4b), indicating that multifunctional C13 and C14 hydroxylase (*doxA*) does not accept modified substrates well, as has been noted recently [55].

Through the use of our comprehensive combinatorial biosynthesis platform, we obtained 16 desired anthracycline analogs (**19**‐**34**) by combining four genetic cassettes. The efficient and balanced expression of the required modules afforded quantitative and efficient conversion to the intended anthracyclines in most cases, facilitating downstream purification and structure elucidation from 1–5 L fermentation. Three additional anthracyclines (**37**‐**39**) were isolated and characterized from the large‐scale cultures during the downstream work. The chemical structures of the isolated compounds (**6**, **19–31,** and **35–39**) were established based on HRESI‐MS and cumulative analyses of 1D (^1^H and ^13^C‐NMR) and 2D (COSY, HSQC, HMBC, TOCSY, and NOESY) NMR spectroscopy (Figure S12–S185 and Table S5–S12), and by comparison with literature [48, 61, 62, 63]. Briefly, the stereochemistry at C‐9 position of the four aglycones were distinguished by NOESY correlations between H‐7/CH_2_‐8, CH_3_‐13 (nogalamycin‐type) or CH_3_‐14 (aklavinone‐type) with H‐10, alongside H‐7 coupling constants and comparison to reference structures, such as nogalamycin (**4**). Glycosylation at the aglycone C‐7 position was confirmed by reciprocal HMBC correlations between H‐7/C‐1’ and H‐1’/C‐7. The sugar configurations were determined by NOESY data, coupling constants, and comparison with literature [48]. In addition, the structures of three known molecules (**32‐**‐**34**) were identified based on their MS spectra and comparison to authentic standards (Figures S186‐S189).

### 2.4 Bioactivity Profiling of Novel Anthracycline Derivatives

We next evaluated the medicinal potency of the newly synthesized anthracyclines. Calculation of the physicochemical properties of the molecules in the library revealed diversity in charge state, logP and pKa (Table S13). We carried out cytotoxicity test of compounds **4–6**, **19–31**, and **35–39** across five human cancer cell lines: A549 (non‐small cell lung), PC3 (prostate), HCT116 (colorectal), and two Merkel cell carcinoma lines, MKL1 and MCC26. Two rounds of preliminary screenings at 80 and 10 µM emphasized the importance of amino sugars attached at the C7 position, and led to exclusion of **5**, **6**, **19**, **27**, **28**, **35**, **36**, and **39** with EC_50_> 10 µM from further analysis (Table 1, Figures S190–S192). Subsequent dose‐response analysis of the remaining compounds (**19–26**, **29–31**, **37–38**), with **1** and **4** as positive controls, identified six glycosylated analogs **22**, **23**, **25**, **26**, **30**, and **31** that harbored good cytotoxic potency (EC_50_ = 1.16–9.38 µM) (Table 1, Figure S192).

**TABLE 1:** Cytotoxic activities of novel anthracyclines.

Compounds	EC_50_ *µ*M			EC_50_ *µ*M	
	A549	PC3	HCT116	MKL1	MCC26
Doxorubicin (**1**)	0.77 ± 0.05	0.33 ± 0.01	0.17 ± 0.03	0.04 ± 0.00	0.22 ± 0.02
Nogalamycin (Nogalamycin A) (**4**)	0.10 ± 0.00	0.07 ± 0.00	0.02 ± 0.00	0.08 ± 0.00	0.06 ± 0.01
Aklavinone (**5**)	>10	>10	>10	>10	>10
Nogalamycinone (**6**)	>10	>10	>10	>10	>10
7‐*O*‐*α* ‐l‐Daunosaminyl‐auramycinone (**19**)	>10	>10	>10	>10	>10
7‐*O*‐*α* ‐l‐Daunosaminyl‐9‐*epi*‐aklavinone (**20**)	>10	>10	>10	>10	>10
7‐*O*‐*α* ‐l‐Daunosaminyl‐nogalamycinone (**21**)	>10	>10	>10	>10	>10
7‐*O*‐*α* ‐l‐Acosaminyl‐9‐*epi*‐aklavinone (**22**)	>10	>10	5.80 ± 1.75	9.38 ± 0.63	>10
7‐*O*‐*α* ‐l‐Acosaminyl‐nogalamycinone (**23**)	>10	>10	>10	4.50 ± 1.91	7.17 ± 0.86
7‐*O*‐*α* ‐l‐Rhodosaminyl‐auramycinone (**24**)	>10	>10	>10	>10	>10
7‐*O*‐*α* ‐l‐Rhodosaminyl‐9‐*epi*‐aklavinone (**25**)	>10	4.86 ± 0.07	1.48 ± 0.50	2.38 ± 0.14	8.08 ± 1.22
7‐*O*‐*α* ‐l‐Rhodosaminyl‐nogalamycinone (**26**)	>10	7.40 ± 1.94	1.56 ± 0.12	1.75 ± 0.08	8.40 ± 0.21
7‐*O*‐*α* ‐l‐3′,4′‐Demethyl‐nogalosyl‐9‐*epi*‐aklavinone (**27**)	>80	>80	>80	>80	>80
7‐*O*‐*α* ‐l‐3′,4′‐Demethyl‐nogalosyl‐aklavinone (**28**)	>80	>80	>80	>80	>80
1‐Hydroxy‐7‐*O*‐*α* ‐l‐daunosaminyl‐nogalamycinone (**29**)	>10	>10	>10	>10	>10
1‐Hydroxy‐7‐*O*‐*α* ‐l‐acosaminyl‐nogalamycinone (**30**)	>10	5.67 ± 0.29	2.59 ± 0.41	1.83 ± 0.04	>10
1‐Hydroxy‐7‐*O*‐*α* ‐l‐rhodosaminyl‐nogalamycinone (**31**)	9.35 ± 1.86	4.49 ± 0.82	1.16 ± 0.03	1.59 ± 0.21	8.31 ± 1.27
7‐Deoxy‐nogalamycinone (**35**)	>80	>80	>80	>80	>80
7‐Deoxy‐auramycinone (**36**)	>80	>80	>80	>80	>80
1‐Hydroxy‐7‐*O*‐*α* ‐l‐rhodosaminyl‐auramycinone (**37**)	>10	>10	>10	>10	>10
*N*‐Acetyl‐7‐*O*‐*α* ‐l‐daunosaminyl‐9‐*epi*‐aklavinone (**38**)	>10	>10	>10	>10	>10
7‐*O*‐*α* ‐l‐Acosaminyl‐aklavinone (**39**)	>10	>10	>10	>10	>10

Cytotoxicity EC_50_ values were obtained after 72 h incubation. All compounds tested in our biological assays showed over 95% purity based on HPLC‐UV/vis profiles (see Supplementary Information). Doxorubicin (**1**) and nogalamycin (**4**) were used as positive controls (*n* = 3).

We next performed in vitro DNA‐binding competition assays to evaluate the capacity of the anthracyclines to disrupt pre‐formed, fluorescent DNA–dye complexes. To probe distinct binding modalities, we utilized two structurally differentiated dyes: PicoGreen, which features a bimodal mechanism combining intercalation with minor‐groove occupancy [64], and Hoechst 33258, a strict minor‐groove binder. In the PicoGreen displacement assay (Figures 5a, S193a), DNA‐binding affinity showed a good correlation with cytotoxicity. The positive control **1** exhibited the strongest DNA‐binding activity, with an IC_50_ of 3.0 µM, which was markedly lower than those of all other anthracyclines tested. Among the remaining compounds, **4**, **23, 25, 26, 30**, and **31** displayed relatively strong displacement activities, with IC_50_ values ranging from 21.6 to 27.7 µM, whereas **5**, **6**, **19**, **27**, **28**, **35**, **36**, **38**, and **39** produced little or no detectable displacement, with IC_50_ values exceeding 80 µM. A similar trend was observed in the Hoechst 33258 displacement assay (Figures 5b, S193b), with **1** again exhibiting the strongest DNA‐binding activity (IC_50_ = 1.2 µM). Broadly, Hoechst 33258 was displaced more readily and at substantially lower concentrations than PicoGreen. Beyond the subset that effectively displaced PicoGreen (**4**, **23**, **25**, **26**, **30**, and **31**), compounds **19**, **20**, **21**, **22**, **24**, **37,** and **39** also efficiently displaced Hoechst 33258 with IC_50_ values below 4 µM. Consistent with the PicoGreen assay, compounds **27**, **28**, and **36** showed no measurable displacement within the tested concentration range.

**FIGURE 5:**
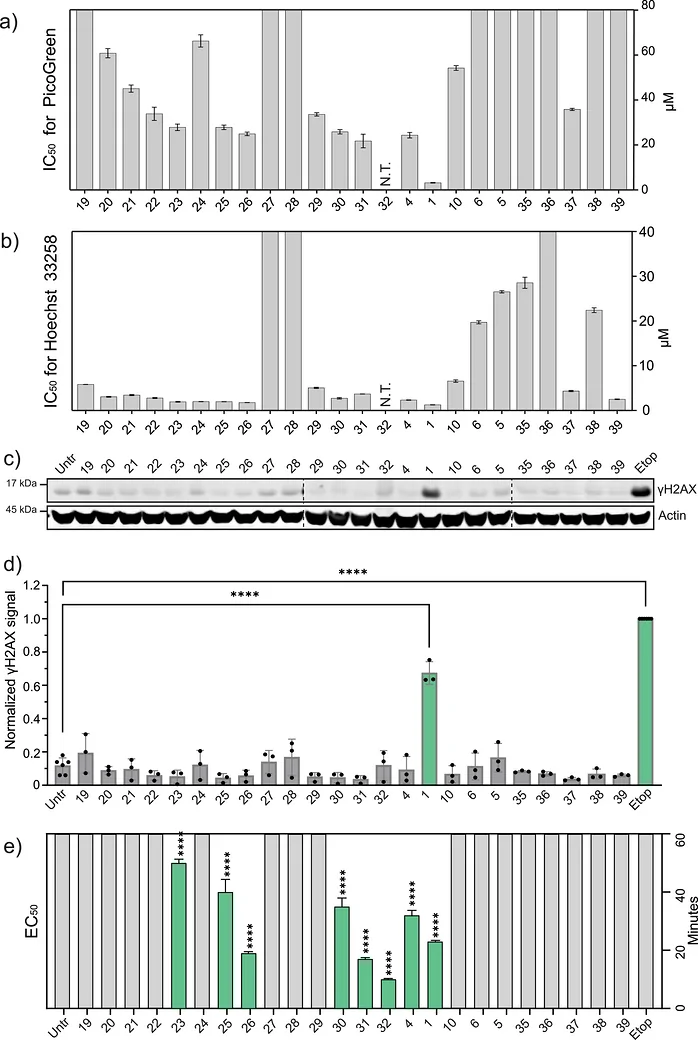
DNA binding, DNA damage, and histone eviction activities of novel anthracyclines. (a) PicoGreen dye competition assay (b) Hoechst33258 dye competition assay. In panels *a* and *b*, the fluorescent dyes bound to double‐stranded DNA exhibit strong fluorescence, which decreases when the dyes are displaced from the DNA–dye complex by anthracyclines. A series of concentrations of anthracyclines were tested, and dose–response curves were generated by nonlinear regression. The DNA‐binding affinity was quantified as IC_50_, defined as the concentration required to reduce the fluorescence intensity of the DNA‐bound dye by 50% (*n* = 3). IC_50_ values exceeding the assay range were plotted at the upper limit of the tested concentration range for visualization. (c) Western blot indicating the γH2AX levels after treatment. MelJuSo cells were treated for 2 h with 10 µM of the indicated compounds. Subsequently, γH2AX levels were detected by Western blot. Actin was used as loading control and position of protein weight markers are indicated. (d) Quantification of γH2AX signal normalized to actin. Ordinary one‐way ANOVA, ****p < 0.0001; grey bars do not show a significant difference with the control sample. (e) The rate of histone eviction activity for indicated compounds. The histone eviction activity was quantified and plotted as EC_50_ (the time at which 50% of the initial signal is reduced). Untreated cells and etoposide‐treated cells were used as negative controls; doxorubicin (**1**) and rhodomycin T (**32**) were used as positive controls [16]. EC_50_ values exceeding the assay range were plotted at the upper limit of the tested concentration range for visualization. Non‐linear regression with one‐way ANOVA with multiple comparisons, ****p < 0.0001, all grey bars do not show significant difference with the untreated control. Known anthracyclines **1**, **4**, and **32**, and the synthetic topoisomerase II poison Etoposide (Etop) were used as positive controls. N.T.: Not tested.

Next, we assessed formation of DSBs in DNA by measuring γH2AX protein levels via Western blot analysis. Production of the phosphorylated γH2AX histone variant is induced during DNA damage and the protein is a widely used marker for detecting DSBs [65]. Specifically, wild‐type MelJuSo cancer cells were treated with the indicated compounds and the known topoisomerase II inhibitor etoposide was used as positive control [66]. Among all tested compounds, only **1** induced γH2AX under these experimental conditions (Figures 5c, d, S193), indicating that the other variants likely do not mediate their biological effect through induction of DSBs in DNA

Finally, we evaluated chromatin damage activity by monitoring histone eviction using photoactivatable GFP‐tagged histone H2A (PAGFP‐H2A) in live‐cell imaging [67]. Fluorescence intensity was measured in real time following treatment with compounds using time‐lapse confocal microscopy. The rate of histone eviction was quantified as the EC_50_ (the time at which fluorescence intensity in the photoactivated region decreased by 50%) (Figure 5e). As previously reported, doxorubicin (**1**) evicted histones with an EC_50_ of 23 min [11, 12, 67, 68]. Notably, six novel variants described here demonstrated histone eviction activity, with **26** and **31** showing greater potency (EC_50_ values of 19 and 17 min, respectively) compared to **1**.

Overall, biological activity profiling illuminated SAR information and established the medicinal potential of these novel anthracyclines. In the cytotoxicity test, we identified six active compounds containing either α‐l‐rhodosamine or α‐l‐acosamine, suggesting their critical roles in cytotoxic activity when attached to the C‐7 position (Table 1). Additionally, 1‐hydroxy derivatives (**30**, **31**) were more active than their corresponding 1‐deoxy forms (**23**, **26**). The PicoGreen DNA dye competition assay indicated that the cytotoxicity is closely correlated with DNA binding affinity (Figure 5a). Both aglycones and sugar units appeared to influence DNA binding affinity. The stereochemistry and substituent pattern at C9 markedly affected DNA binding, which is consistent with earlier reports promoting the importance of C9 for stabilization of the anthracycline–DNA complex [69]. Interestingly, compounds with 9R configuration (i.e., aklavinone‐ and auramycinone‐type compounds) exhibited significantly lower DNA‐binding affinities than their 9S counterparts (i.e., 9‐*epi*‐aklavinone and nogalamycinone‐type compounds). This stereochemical impact is well exemplified by comparing compounds **10**, **24**, **25,** and **26**, which differ exclusively at C9. In effect, compounds with auramycinone‐type scaffolds **19**, **24,** and **37** could only displace the minor groove binding Hoechst 33258 dye, but not the intercalating PicoGreen (Figure 5a, b) under the concentrations tested. Oxidative tailoring of the aglycone at C1/C13/C14 appeared to further enhance DNA binding affinity [69], possibly by providing additional hydrogen bonding possibilities with DNA. In terms of carbohydrate units, all compounds with amino sugars (excluding compound **38** with an unexpected bulky acyl group) were able to displace the Hoechst 33 258 dye, supporting the critical role of hydrogen‐bonding interactions between the amino sugar and the DNA minor groove [70]. Meanwhile, aglycones and anthracyclines containing neutral sugars (**5**, **6**, **27**, and **28**) showed weak or no DNA binding.

In terms of anthracycline induced DNA damage, the molecular basis has remained unresolved due to challenges in obtaining structures of anthracycline‐DNA‐Topo II complexes to determine the catalytic step at which the enzyme is poisoned [14]. Indirect evidence suggests that **1** induces DNA damage by stabilizing a late‐stage Topo II re‐ligation complex similarly to etoposide [71], while other anthracyclines may poison the enzyme at an earlier step without causing DSB. Early reports have even suggested that **2** may prevent the binding of Topo II to DNA entirely [72]. Previous studies have highlighted the importance of carbohydrate *N,N*‐dimethylation in prevention of DSB formation in aklavinone based compounds. However, our work focusing on non‐aklavinone scaffolds demonstrates that the aglycone unit can also influence DNA damage, since **19**, **20,** and **21** appended with α‐l‐daunosamine did not cause DSB (Figure 5d), although this observation should be considered with the caveat that the lower cytotoxic activity (Table 1) may have contributed to the lack of DNA damage. Nonetheless, it is noteworthy that the two most potent novel anthracyclines based on 9‐*epi*‐aklavinone (**25**) and nogalamycinone (**26**) scaffolds containing α‐l‐rhodosamine (Table 1) did not induce DNA damage (Figure 5d).

Recent data has suggested that the cytotoxicity of anthracyclines arises primarily from chromatin damage rather than DNA damage and that the carbohydrate units of anthracyclines are prominent in histone eviction activity [12]. We observed strong correlation between cytotoxicity (Table 1), DNA binding efficiency and histone eviction (Figure 5e), with compounds bearing α‐l‐acosamine or α‐l‐rhodosamine displayed greater potency than those containing α‐l‐daunosamine. The correlation extended even to the type of the aglycone scaffold: Auramycinone‐based compounds failed to promote histone eviction, whereas derivatives of 9‐*epi*‐aklavinone and nogalamycinone exhibited pronounced chromatin‐disruption potential (Figure 5e). We also observed that histone eviction was enhanced by specific aglycone tailoring modifications with C1‐hydroxylation demonstrating a moderate effect, while C11‐hydroxylation significantly increased the activity [57]. While the precise mechanisms underlying the distinct bioactivity profile of anthracyclines with α‐l‐rhodosamine remain unclear, our results align with reports that N,N‐dimethylation may alter anthracycline‐DNA interactions [57]. Beyond direct binding effects, N,N‐dimethylation may abolish formaldehyde‐linked aminal adduct formation of α‐l‐daunosamine at guanine N^2^, possibly shifting the binding mode to reversible intercalation and altering the dynamics of DNA–histone eviction [57].

## 3 Conclusion

We have developed a robust synthetic biology platform for the complete biosynthesis of anthracyclines. This bottom‐up system integrates a genetically tractable *Streptomyces* host, modular expression constructs, a set of finely tunable promoters, effective terminators, and a curated collection of biosynthetic genes specific to anthracyclines. This work demonstrates that complex natural product biosynthetic pathways can be completely refactored in actinomycetes. By functionally reorganizing these pathways into multi‐gene modules of polyketide aglycones, self‐defense and GTs, TDP‐carbohydrates, and post‐PKS tailoring, we successfully achieved the complete biosynthesis of three important anthracyclines: nogalamycin, doxorubicin, and aclacinomycin. This marks the first time, such complex biosynthetic pathways, involving up to 38 genes, have been fully reconstituted from synthetic DNA in a *Streptomyces* heterologous system.

The modular nature of our platform facilitated streamlined combinatorial biosynthesis, leading to 13 novel compounds through strategic reshuffling of biosynthetic modules for novel anthracycline derivatives. The novel molecules could be created rapidly in a period of a few months, since the process did not require additional gene cloning steps; the functionalities could be repurposed from existing BioBricks modules and libraries. In effect, the limiting factor for the platform appears to be structure elucidation of the anthracyclines, despite the clean production profiles achieved through our Design‐Build‐Test‐Learn development cycles with well‐characterized promoters of varying strengths and orthologous genes (Figure 4b). The modularity of the platform solved some of the fundamental issues impeding previous efforts to achieve combinatorial biosynthesis of anthracyclines [73, 74, 75, 76, 77, 78], where cloning and co‐expression of large complete anthracycline gene clusters from a single expression vector remained arduous. These challenges necessitated the development of alternative strategies in the past, such as bioconversion and co‐cultivation [79, 80], which are however not ideal for large scale manufacturing due the increased complexity of the fermentation processes.

The bioactivity assays led to the discovery of two novel anthracycline analogs (**26**, **31**), which demonstrated the desired activity profile for next‐generation anthracyclines; the compounds evicted histones faster than **1**, but did not cause DNA damage. The cytotoxicity of the hit molecules was generally lower than those of the reference anthracyclines, such as **1** and **4**, but it is noteworthy that the improved SAR understanding provides a robust starting point for further development rounds for improving the cytotoxicity without compromising the desired bioactivity profile. The synthetic biology platform presented here is well‐suited for lead development and allows combinatorial exploration of (i) further redox tailoring reactions, (ii) bi‐glycosylated anthracyclines through inclusion of additional GTs, and (iii) discovery of novel modification through the use of genetic material from uncharacterized unknown anthracycline BGCs. Overall, the presented approach offers a scalable and systematic strategy for reconstituting complex biosynthetic pathways and accelerating the discovery of new bioactive molecules. This modular approach is readily applicable to other families of glycosylated aromatic polyketides, highlighting its broad potential in natural product biosynthesis and drug development.

## Author Contributions

**Heli Tirkkonen**: investigation, methodology, data curation, writing – review and editing. **Isaline Fagon**: investigation. **Sabina Y. van der Zanden**: investigation, methodology, data curation, writing – original draft. **Kendall Paige**: investigation. **Courtney L. Brown**: investigation. **Jacob Hecht**: investigation. **Rongbin Wang**: writing – original draft, investigation, methodology, visualization, data curation. **Jon S. Thorson**: resources, writing – review and editing, funding acquisition. **Khaled A. Shaaban**: investigation, writing – review and editing, supervision, resources, data curation, methodology, conceptualization, project administration. **Leslie Fève**: investigation. **S. Eric Nybo**: writing – review and editing, investigation, funding acquisition, supervision, resources, conceptualization, project administration. **Mikko Metsä‐ketelä**: conceptualization, funding acquisition, writing – original draft, writing – review and editing, supervision, resources, project administration. **Larissa Ponomareva**: investigation, formal analysis, writing – review and editing. **Nora Schwartz**: investigation. **Jacques Neefjes**: funding acquisition, supervision, resources, writing – review and editing. **Jennifer Nguyen**: investigation.

## Conflicts of Interest

The authors declare no conflicts of interest.

## Supporting information



The authors have cited additional references within the Supporting Information [81, 82, 83, 84, 85, 86, 87, 88, 89, 90, 91, 92, 93, 94, 95, 96, 97].**Supporting File**: anie74966‐sup‐0001‐SuppMat.docx.

## References

[1] D. J. Newman and G. M. Cragg , “Natural Products as Sources of New Drugs Over the Nearly Four Decades From 01/1981 to 09/2019/2019,” Journal of Natural Products 83 (2020): 770–803, 10.1021/acs.jnatprod.9b01285. 32162523

[2] F. Arcamone , G. Cassinelli , G. Fantini , et al., “Adriamycin, 14‐Hydroxydaimomycin, a New Antitumor Antibiotic From S. Peucetius Var. Caesius,” Biotechnology and Bioengineering 11 (1969): 1101–1110, 10.1002/bit.260110607. 5365804

[3] A. Murzyn , J. Orzeł , N. Obajtek , et al., “Aclarubicin: Contemporary Insights Into Its Mechanism of Action, Toxicity, Pharmacokinetics, and Clinical Standing,” Cancer Chemotherapy and Pharmacology 94 (2024): 123–139, 10.1007/s00280-024-04693-1. PMC11390774 38965080

[4] M. B. Hulst , T. Grocholski , J. J. C. Neefjes , G. P. Van Wezel , and M. Metsä‐Ketelä , “Anthracyclines: Biosynthesis, Engineering and Clinical Applications,” Natural Product Reports 39 (2022): 814–841, 10.1039/D1NP00059D. 34951423

[5] A. Moreno‐Arciniegas , A. García , M. Kelm , et al., RESILIENCE Trial Investigators , “Rationale and Design of RESILIENCE : A Prospective Randomized Clinical Trial Evaluating Remote Ischaemic Conditioning for the Prevention of Anthracycline Cardiotoxicity,” European J of Heart Fail 26 (2024): 2213–2222. 10.1002/ejhf.3395 39212445

[6] S. Zhang , X. Liu , T. Bawa‐Khalfe , et al., “Identification of the Molecular Basis of Doxorubicin‐Induced Cardiotoxicity,” Nature Medicine 18 (2012): 1639–1642, 10.1038/nm.2919. 23104132

[7] M. Volkova and R. Russell , “Anthracycline Cardiotoxicity: Prevalence, Pathogenesis and Treatment,” CCR 7 (2012): 214–220, 10.2174/157340311799960645. PMC3322439 22758622

[8] D. A. Mulrooney , M. W. Yeazel , T. Kawashima , et al., “Cardiac Outcomes in a Cohort of Adult Survivors of Childhood and Adolescent Cancer: Retrospective Analysis of the Childhood Cancer Survivor Study Cohort,” BMJ 339 (2009): b4606–b4606, 10.1136/bmj.b4606. PMC3266843 19996459

[9] S. M. Swain , F. S. Whaley , and M. S. Ewer , “Congestive Heart Failure in Patients Treated with Doxorubicin,” Cancer 97 (2003): 2869–2879, 10.1002/cncr.11407. 12767102

[10] C. M. Larsen , M. Garcia Arango , H. Dasari , et al., “Association of Anthracycline With Heart Failure in Patients Treated for Breast Cancer or Lymphoma, 1985‐2010,” JAMA Network Open 6 (2023): e2254669, 10.1001/jamanetworkopen.2022.54669, e2254669. PMC9898820 36735254

[11] X. Qiao , S. Y. Van Der Zanden , X. Li , et al., “Diversifying the Anthracycline Class of Anti‐cancer Drugs Identifies Aclarubicin for Superior Survival of Acute Myeloid Leukemia Patients,” Molecular Cancer 23 (2024): 120, 10.1186/s12943-024-02034-7. PMC11149191 38831402

[12] X. Qiao , S. Y. Van Der Zanden , D. P. A. Wander , et al., “Uncoupling DNA Damage From Chromatin Damage to Detoxify Doxorubicin,” Proceedings of the National Academy of Sciences of the United States of America 117 (2020): 15182–15192, 10.1073/pnas.1922072117. PMC7334570 32554494

[13] G. L. Beretta and F. Zunino in , “(Ed.: K. Krohn), Springer Berlin Heidelberg, Berlin, Heidelberg,” (2008): 1–19.

[14] S. Y. Van Der Zanden , X. Qiao , and J. Neefjes , “New Insights Into the Activities and Toxicities of the Old Anticancer Drug Doxorubicin,” FEBS Journal 288 (2021): 6095–6111. 10.1111/febs.15583 PMC8597086 33022843

[15] J. W. Lown , “Discovery and Development of Anthracycline Antitumour Antibiotics,” Chemistry Society Reviews 22 (1993): 165, 10.1039/cs9932200165.

[16] M. A. Van Gelder , S. Y. Van Der Zanden , M. B. L. Vriends , et al., “Re‐Exploring the Anthracycline Chemical Space for Better Anti‐Cancer Compounds,” Journal of Medicinal Chemistry 66 (2023): 11390–11398. 10.1021/acs.jmedchem.3c00853 PMC10461226 37561481

[17] M. B. Martins‐Teixeira and I. Carvalho , “Antitumour Anthracyclines: Progress and Perspectives,” ChemMedChem 15 (2020): 933–948, 10.1002/cmdc.202000131. 32314528

[18] L. Li , L. W. Maclntyre , and S. F. Brady , “Refactoring Biosynthetic Gene Clusters for Heterologous Production of Microbial Natural Products,” Current Opinion in Biotechnology 69 (2021): 145–152, 10.1016/j.copbio.2020.12.011. PMC8238852 33476936

[19] E. Kim , B. S. Moore , and Y. J. Yoon , “Reinvigorating Natural Product Combinatorial Biosynthesis With Synthetic Biology,” Nature Chemical Biology 11 (2015): 649–659, 10.1038/nchembio.1893. PMC4757526 26284672

[20] H.‐S. Kang and E.‐S. Kim , “Recent Advances in Heterologous Expression of Natural Product Biosynthetic Gene Clusters in Streptomyces Hosts,” Current Opinion in Biotechnology 69 (2021): 118–127, 10.1016/j.copbio.2020.12.016. 33445072

[21] R. Wang , J. Nguyen , J. Hecht , et al., “A BioBricks Metabolic Engineering Platform for the Biosynthesis of Anthracyclinones in *Streptomyces coelicolor* ,” ACS Synthetic Biology 11 (2022): 4193–4209, 10.1021/acssynbio.2c00498. PMC9764417 36378506

[22] J. P. Gomez‐Escribano and M. J. Bibb , “Engineering *Streptomyces coelicolor* for Heterologous Expression of Secondary Metabolite Gene Clusters,” Microbial Biotechnology 4 (2011): 207–215, 10.1111/j.1751-7915.2010.00219.x. PMC3818861 21342466

[23] S. Hwang , Y. Lee , J. H. Kim , et al., “Streptomyces as Microbial Chassis for Heterologous Protein Expression,” Frontiers in Bioengineering and Biotechnology 9 (2021): 804295, 10.3389/fbioe.2021.804295. PMC8724576 34993191

[24] C. Bai , Y. Zhang , X. Zhao , et al., “Exploiting a Precise Design of Universal Synthetic Modular Regulatory Elements to Unlock the Microbial Natural Products in Streptomyces,” Proceedings of the National Academy of Sciences of the United States of America 112 (2015): 12181–12186, 10.1073/pnas.1511027112. PMC4593075 26374838

[25] V. Siitonen , M. Claesson , P. Patrikainen , et al., “Identification of Late‐Stage Glycosylation Steps in the Biosynthetic Pathway of the Anthracycline Nogalamycin,” ChemBioChem 13 (2012): 120–128, 10.1002/cbic.201100637. 22120896

[26] M. Metsä‐Ketelä , J. Niemi , P. Mäntsälä , and G. Schneider , “Anthracycline Biosynthesis: Genes, Enzymes and Mechanisms,” Topics in Current Chemistry 282 (2008): 101–140, 10.1007/978-3-540-75815-0.

[27] K. V. Brown , B. N. Wandi , M. Metsä‐Ketelä , and S. E. Nybo , “Pathway Engineering of Anthracyclines: Blazing Trails in Natural Product Glycodiversification,” Journal of Organic Chemistry 85 (2020): 12012–12023, 10.1021/acs.joc.0c01863. PMC7541800 32938175

[28] H. Tirkkonen , K. V. Brown , M. Niemczura , et al., “Engineering BioBricks for Deoxysugar Biosynthesis and Generation of New Tetracenomycins,” ACS Omega 8 (2023): 21237–21253, 10.1021/acsomega.3c02460. PMC10269268 37332790

[29] J. Niemi , M. Metsä‐Ketelä , G. Schneider , and P. Mäntsälä , “Biosynthetic Anthracycline Variants,” Topics in Current Chemistry 282 (2008): 75–99, 10.1007/128_2007_13.

[30] R. Wang , B. Nji Wandi , N. Schwartz , et al., “Diverse Combinatorial Biosynthesis Strategies for C‐H Functionalization of Anthracyclinones,” ACS Synthetic Biology 13 (2024): 1523–1536. 10.1021/acssynbio.4c00043 PMC11101304 38662967

[31] H. Laatsch , Fotso in Anthracycline Chemistry and Biology I (Ed.: K. Krohn) (Springer, 2008), 3–74.

[32] R. H. Baltz , “Renaissance in Antibacterial Discovery from Actinomycetes,” Current Opinion in Pharmacology 8 (2008): 557–563, 10.1016/j.coph.2008.04.008. 18524678

[33] L. Li , W. Jiang , and Y. Lu in , Synthetic Metabolic Pathways (Eds.: M.K. Jensen, J.D. Keasling) (Springer, 2018), 203–209, 10.1007/978-1-4939-7295-1.

[34] Y. Parag , “Genetic Recombination in Streptomyces Griseus,” Journal of Bacteriology 133 (1978): 1027–1031, 10.1128/jb.133.2.1027-1031.1978. PMC222116 415037

[35] J. T. Nguyen , K. K. Riebschleger , K. V. Brown , N. M. Gorgijevska , and S. E. Nybo , “A BioBricks Toolbox for Metabolic Engineering of the Tetracenomycin Pathway,” Biotechnology Journal 17 (2022): 2100371, 10.1002/biot.202100371. PMC8920762 34719127

[36] C. Aubry , J. L. Pernodet , and S. Lautru , “Modular and Integrative Vectors for Synthetic Biology Applications in *Streptomyces* spp,” Applied and Environmental Microbiology 85, no. 16 (2019): e00485–19, 10.1128/AEM.00485-19. PMC6677859 31175189

[37] N. Lomovskaya , S. K. Hong , S. U. Kim , L. Fonstein , K. Furuya , and R. C. Hutchinson , “The *Streptomyces peucetius* drrC Gene Encodes a UvrA‐Like Protein Involved in Daunorubicin Resistance and Production,” Journal of Bacteriology 178 (1996): 3238–3245, 10.1128/jb.178.11.3238-3245.1996. PMC178076 8655504

[38] P. G. Guilfoile and C. R. Hutchinson , “A Bacterial Analog of the Mdr Gene of Mammalian Tumor Cells Is Present in *Streptomyces peucetius*, the Producer of Daunorubicin and Doxorubicin,” Proceedings of the National Academy of Sciences 88 (1991): 8553–8557, 10.1073/pnas.88.19.8553. PMC52547 1924314

[39] E. Tenconi and S. Rigali , “Self‐Resistance Mechanisms to DNA‐Damaging Antitumor Antibiotics in Actinobacteria,” Current Opinion in Microbiology 45 (2018): 100–108, 10.1016/j.mib.2018.03.003. 29642052

[40] S. L. Otten , X. Liu , J. Ferguson , and C. R. Hutchinson , “Cloning and Characterization of the Streptomyces Peucetius dnrQS Genes Encoding a Daunosamine Biosynthesis Enzyme and a Glycosyl Transferase Involved in Daunorubicin Biosynthesis,” Journal of Bacteriology 177 (1995): 6688–6692, 10.1128/jb.177.22.6688-6692.1995. PMC177529 7592454

[41] K. Räty , J. Kantola , A. Hautala , J. Hakala , K. Ylihonko , and P. Mäntsälä , “Cloning and Characterization of *Streptomyces galilaeus* Aclacinomycins Polyketide Synthase (PKS) Cluster,” Gene 293 (2002): 115–122, 10.1016/S0378-1119(02)00699-6. 12137949

[42] W. Lu , C. Leimkuhler , M. Oberthür , D. Kahne , and C. T. Walsh , “AknK Is an L‐2‐deoxyfucosyltransferase in the Biosynthesis of the *Anthracycline aclacinomycin* A,” Biochemistry 43 (2004): 4548–4558, 10.1021/bi035945i. 15078101

[43] M. Claesson , V. Siitonen , D. Dobritzsch , M. Metsä‐Ketelä , and G. Schneider , “Crystal Structure of the Glycosyltransferase SnogD From the Biosynthetic Pathway of Nogalamycin in Streptomyces Nogalater,” FEBS Journal 279 (2012): 3251–3263, 10.1111/j.1742-4658.2012.08711.x. 22804797

[44] K. Räty , A. Hautala , S. Torkkell , et al., “Characterization of Mutations in Aclacinomycin A‐Non‐Producing Streptomyces Galilaeus Strains With Altered Glycosylation Patterns,” Microbiology 148 (2002): 3375–3384, 10.1099/00221287-148-11-3375. 12427929

[45] J. D. Kittendorf and D. H. Sherman , “The Methymycin/Pikromycin Pathway: a Model for Metabolic Diversity in Natural Product Biosynthesis,” Bioorganic & Medicinal Chemistry 17 (2009): 2137–2146, 10.1016/j.bmc.2008.10.082. PMC2843759 19027305

[46] V. Siitonen , B. Nji Wandi , A.‐P. Törmänen , and M. Metsä‐Ketelä , “Enzymatic Synthesis of the C‐Glycosidic Moiety of Nogalamycin R,” ACS Chemical Biology 13 (2018): 2433–2437, 10.1021/acschembio.8b00658. PMC6203184 30114358

[47] L. Shao , J. Huang , L. Jing , et al., “Overexpression of aveBIV Leading to the Improvement of 4′‐ epidaunorubicin Production in Streptomyces Coeruleorubidus Strain SIPI‐A0707,” Applied Microbiology and Biotechnology 87 (2010): 1057–1064, 10.1007/s00253-010-2541-3. 20390414

[48] V. Siitonen , B. Blauenburg , P. Kallio , P. Mäntsälä , and M. Metsä‐Ketelä , “Discovery of a Two‐Component Monooxygenase SnoaW/SnoaL2 Involved in Nogalamycin Biosynthesis,” Chemistry & Biology 19 (2012): 638–646, 10.1016/j.chembiol.2012.04.009. 22633415

[49] V. Siitonen , B. Selvaraj , L. Niiranen , Y. Lindqvist , G. Schneider , and M. Metsä‐Ketelä , “Divergent Non‐Heme Iron Enzymes in the Nogalamycin Biosynthetic Pathway,” Proceedings of the National Academy of Sciences 113 (2016): 5251–5256, 10.1073/pnas.1525034113. PMC4868444 27114534

[50] B. Nji Wandi , V. Siitonen , K. Palmu , and M. Metsä‐Ketelä , “The Rieske Oxygenase SnoT Catalyzes 2′′‐hydroxylation of L‐rhodosamine in Nogalamycin Biosynthesis,” Chembiochem 21 (2020): 1–6. 10.1002/cbic.202000229 32557994

[51] A. Jansson , H. Koskiniemi , P. Mäntsälä , J. Niemi , and G. Schneider , “Crystal Structure of a Ternary Complex of DnrK, a Methyltransferase in Daunorubicin Biosynthesis, With Bound Products,” Journal of Biological Chemistry 279 (2004): 41149–41156, 10.1074/jbc.M407081200. 15273252

[52] J. Niemi , Y. Wang , K. Airas , K. Ylihonko , J. Hakala , and P. Mäntsälä , “Characterization of Aklavinone‐11‐hydroxylase From *Streptomyces purpurascens* ,” Biochimica et Biophysica Acta (BBA)—Protein Structure and Molecular Enzymology 1430 (1999): 57–64. 10.1016/s0167-4838(98)00265-9 10082933

[53] K. Madduri and C. R. Hutchinson , “Functional Characterization and Transcriptional Analysis of a Gene Cluster Governing Early and Late Steps in Daunorubicin Biosynthesis in Streptomyces Peucetius,” Journal of Bacteriology 177 (1995): 3879–3884, 10.1128/jb.177.13.3879-3884.1995. PMC177111 7601857

[54] N. Lomovskaya , S. L. Otten , Y. Doi‐Katayama , et al., “Doxorubicin Overproduction in Streptomyces Peucetius: Cloning and Characterization of the dnrU Ketoreductase and dnrV Genes and the doxA Cytochrome P‐450 Hydroxylase Gene,” Journal of Bacteriology 181 (1999): 305–318, 10.1128/JB.181.1.305-318.1999. PMC103563 9864344

[55] M. B. Hulst , L. Zhang , H. U. Van Der Heul , et al., “Metabolic Engineering of Streptomyces Peucetius for Biosynthesis of N,N‐dimethylated Anthracyclines,” Frontiers in Bioengineering and Biotechnology 12 (2024): 1363803, 10.3389/fbioe.2024.1363803. PMC10936713 38481571

[56] J. Rohr , “Combinatorial Biosynthesis–An Approach in the Near Future?,” Angewandte Chemie International Edition in English 34 (1995): 881–885, 10.1002/anie.199508811.

[57] D. P. A. Wander , S. Y. Van Der Zanden , G. A. Van Der Marel , H. S. Overkleeft , J. Neefjes , and J. D. C. Codée , “Doxorubicin and Aclarubicin: Shuffling Anthracycline Glycans for Improved Anticancer Agents,” Journal of Medicinal Chemistry 63 (2020): 12814–12829, 10.1021/acs.jmedchem.0c01191. PMC7667640 33064004

[58] L. L. Lairson , B. Henrissat , G. J. Davies , and S. G. Withers , “Glycosyltransferases: Structures, Functions, and Mechanisms,” Annual Review of Biochemistry 77 (2008): 521–555, 10.1146/annurev.biochem.76.061005.092322. 18518825

[59] Z. Zhang , Y. K. Gong , Q. Zhou , et al., “Hydroxyl Regioisomerization of Anthracycline Catalyzed by a Four‐Enzyme Cascade,” Proceedings of the National Academy of Sciences of the United States of America 114 (2017): 1554–1559, 10.1073/pnas.1610097114. PMC5321016 28137838

[60] Y. Wang , J. Niemi , and P. Mäntsälä , “Modification of Aklavinone and Aclacinomycins In Vitro and In Vivo by Rhodomycin Biosynthesis Gene Products,” FEMS Microbiology Letters 208 (2002): 117–122, 10.1111/j.1574-6968.2002.tb11070.x. 11934504

[61] M. K. Kharel , P. Pahari , M. D. Shepherd , et al., “Angucyclines: Biosynthesis, Mode‐of‐Action, New Natural Products, and Synthesis,” Natural Product Reports 29 (2012): 264–325, 10.1039/C1NP00068C. PMC11412254 22186970

[62] E. Jalali , F. Wang , B. R. Overbay , et al., “Biochemical and Structural Studies of the Carminomycin 4‐*O*‐methyltransferase DnrK,” Journal of Natural Products 87 (2024): 798–809. 10.1021/acs.jnatprod.3c00947 PMC11623920 38412432

[63] S. I. Elshahawi , K. A. Shaaban , M. K. Kharel , and J. S. Thorson , “A Comprehensive Review of Glycosylated Bacterial Natural Products,” Chemistry Society Reviews 44 (2015): 7591–7697, 10.1039/C4CS00426D. PMC4560691 25735878

[64] Y. Wang , H. Schellenberg , V. Walhorn , K. Toensing , and D. Anselmetti , “Binding Mechanism of PicoGreen to DNA Characterized by Magnetic Tweezers and Fluorescence Spectroscopy,” European Biophysics Journal 46 (2017): 561–566, 10.1007/s00249-017-1204-z. 28251265

[65] L. J. Kuo and L.‐X. Yang , “Gamma‐H2AX—A Novel Biomarker for DNA Double‐Strand Breaks,” In Vivo 22 (2008): 305–309. 18610740

[66] T. T. Le , M. Wu , J. H. Lee , et al., “Etoposide Promotes DNA Loop Trapping and Barrier Formation by Topoisomerase II,” Nature Chemical Biology 19 (2023): 641–650, 10.1038/s41589-022-01235-9. PMC10154222 36717711

[67] B. Pang , X. Qiao , L. Janssen , et al., “Drug‐Induced Histone Eviction From Open Chromatin Contributes to the Chemotherapeutic Effects of Doxorubicin,” Nature Communications 4 (2013): 1908, 10.1038/ncomms2921. PMC3674280 23715267

[68] M. A. Van Gelder , Y. Li , D. P. A. Wander , et al., “Novel *N,N* ‐dimethyl‐idarubicin Analogues Are Effective Cytotoxic Agents for ABCB1‐Overexpressing, Doxorubicin‐resistant Cells,” Journal of Medicinal Chemistry 67 (2024): 13802–13812. 10.1021/acs.jmedchem.4c00614 PMC11345819 39088428

[69] J. B. Chaires , S. Satyanarayana , D. Suh , I. Fokt , T. Przewloka , and W. Priebe , “Parsing the Free Energy of Anthracycline Antibiotic Binding to DNA,” Biochemistry 35 (1996): 2047–2053, 10.1021/bi952812r. 8652545

[70] E. J. Gabbay , D. Grier , R. E. Fingerle , et al., “Interaction Specificity of the Anthracyclines With Deoxyribonucleic Acid,” Biochemistry 15 (1976): 2062–2070, 10.1021/bi00655a006. 776212

[71] C. C. Wu , T. K. Li , L. Farh , et al., “Structural Basis of Type II Topoisomerase Inhibition by the Anticancer Drug Etoposide,” Science 333 (2011): 459–462, 10.1126/science.1204117. 21778401

[72] B. S. Sørensen , J. Sinding , A. H. Andersen , J. Alsner , P. B. Jensen , and O. Westergaard , “Mode of Action of Topoisomerase II‐Targeting Agents at a Specific DNA Sequence,” Journal of Molecular Biology 228 (1992): 778–786. 10.1016/0022-2836(92)90863-f 1335085

[73] K. K. Jain , E. S. Casper , N. L. Geller , et al., “A Prospective Randomized Comparison of Epirubicin and Doxorubicin in Patients With Advanced Breast Cancer,” JCO 3 (1985): 818–826, 10.1200/JCO.1985.3.6.818. 3859587

[74] J. Niemi , K. Ylihonko , J. Hakala , R. Parssinen , A. Kopio , and P. Mantsala , “Hybrid Anthracycline Antibiotics: Production of New Anthracyclines by Cloned Genes From *Streptomyces purpurascens* in *Streptomyces galilaeus* ,” Microbiology 140 (1994): 1351–1358, 10.1099/00221287-140-6-1351. 8081500

[75] T. Kunnari , J. Niemi , K. Ylihonko , P. Mäntsälä , and J. Hakala , “Hybrid Anthracyclines by a Genetically Engineered *Streptomyces galilaeus* Mutant,” Bioorganic & Medicinal Chemistry Letters 7 (1997): 725–726, 10.1016/S0960-894X(97)00092-9.

[76] H. S. Kim , Y. S. Hong , Y. H. Kim , O. J. Yoo , and J. J. Lee , “New Anthracycline Metabolites Produced by the Aklavinone 11‐hydroxylase Gene in *Streptomyces Galilaeus* ATCC 31133,” Journal of Antibiotics 49 (1996): 355–360, 10.7164/antibiotics.49.355. 8641998

[77] T. J. Kunnari , K. P. J. Ylihonko , K. D. Klika , P. I. Mäntsälä , and J. M. L. Hakala , “Hybrid Anthracyclines From a Genetically Engineered *Streptomyces galilaeus* Mutant,” Journal of Organic Chemistry 65 (2000): 2851–2855. 10.1021/jo980345o 10814169

[78] T. Cui , C. Jiang , J. Li , et al., “Modular Engineering of the Doxorubicin Biosynthetic Gene Cluster Based on Component Functional Clustering in Streptomyces,” Journal of Biotechnology 408 (2025): 265–274, 10.1016/j.jbiotec.2025.09.019. 41046982

[79] E. Kim , M. C. Song , M. S. Kim , et al., “One‐Pot Combinatorial Biosynthesis of Glycosylated Anthracyclines by Cocultivation of *Streptomyces* Strains Producing Aglycones and Nucleotide Deoxysugars,” ACS Comb Sci 19 (2017): 262–270. 10.1021/acscombsci.6b00194 28191923

[80] A. R. Han , J. W. Park , M. K. Lee , et al., “Development of a *Streptomyces venezuelae*‐Based Combinatorial Biosynthetic System for the Production of Glycosylated Derivatives of Doxorubicin and Its Biosynthetic Intermediates,” Applied and Environmental Microbiology 77 (2011): 4912–4923, 10.1128/AEM.02527-10. PMC3147398 21602397

[81] T. Kieser , M. J. Bibb , M. J. Buttner , K. F. Chater , and D. A. Hopwood , Practical Streptomyces Genetics (John Innes Foundation Norwich, 2000).

[82] J. F. Castro , V. Razmilic , J. P. Gomez‐Escribano , B. Andrews , J. A. Asenjo , and M. J. Bibb , “Identification and Heterologous Expression of the Chaxamycin Biosynthesis Gene Cluster From *Streptomyces leeuwenhoekii* ,” Applied and Environmental Microbiology 81 (2015): 5820–5831, 10.1128/AEM.01039-15. PMC4551226 26092459

[83] K. A. Shaaban , X. Wang , S. I. Elshahawi , et al., “Herbimycins D–F, Ansamycin Analogues From Streptomyce s sp. RM‐7‐15, Ansamycin Analogues From *Streptomyces* sp. RM‐7‐15,” Journal of Natural Products 76 (2013): 1619–1626, 10.1021/np400308w. PMC3852429 23947794

[84] X. Wang , K. A. Shaaban , S. I. Elshahawi , et al., “Frenolicins C–G, Pyranonaphthoquinones From Streptomyces sp. RM‐4‐15, Pyranonaphthoquinones From *Streptomyces* sp. RM‐4‐15,” Journal of Natural Products 76 (2013): 1441–1447, 10.1021/np400231r. PMC3862173 23944931

[85] K. A. Shaaban , S. I. Elshahawi , X. Wang , et al., “Cytotoxic Indolocarbazoles From *Actinomadura melliaura* ATCC 39691,” Journal of Natural Products 78 (2015): 1723–1729. 10.1021/acs.jnatprod.5b00429 PMC4515175 26091285

[86] D. C. Savi , K. A. Shaaban , F. M. W. R. Gos , et al., “Phaeophleospora Vochysiae Savi & Glienke sp. nov. Isolated From Vochysia Divergens Found in the Pantanal, Brazil, Produces Bioactive Secondary Metabolites,” Scientific Reports 8 (2018): 3122, 10.1038/s41598-018-21400-2. PMC5814415 29449610

[87] Y. Zhang , M. T. Cheema , L. V. Ponomareva , et al., “Himalaquinones A–G, Angucyclinone‐Derived Metabolites Produced by the Himalayan Isolate Streptomyces sp. PU‐MM59, Angucyclinone‐derived Metabolites Produced by the Himalayan Isolate *Streptomyces* sp. PU‐MM59,” Journal of Natural Products 84 (2021): 1930–1940, 10.1021/acs.jnatprod.1c00192. PMC8565601 34170698

[88] Z. Fang , S. Chen , Y. Zhu , et al., “A New Uridine Derivative and a New Indole Derivative From the Coral‐associated *Actinomycete pseudonocardia* sp. SCSIO 11457. SCSIO 11457,” Natural Product Research 35 (2021): 188–194, 10.1080/14786419.2019.1616729. 31137966

[89] W. Wang , X. Li , J. Wang , S. Xiang , X. Feng , and K. Yang , “An Engineered Strong Promoter for Streptomycetes,” Applied and Environmental Microbiology 79 (2013): 4484–4492, 10.1128/AEM.00985-13. PMC3697493 23686264

[90] M. J. Bibb , G. R. Janssen , and J. M. Ward , “Cloning and Analysis of the Promoter Region of the Erythromycin Resistance Gene (ermE) of Streptomyces Erythraeus,” Gene 38 (1985): 215–226, 10.1016/0378-1119(85)90220-3. 2998943

[91] G. Labes , M. Bibb , and W. Wohlleben , “Isolation and Characterization of a Strong Promoter Element From the *Streptomyces ghanaensis* Phage I19 Using the Gentamicin Resistance Gene (aacC1) of Tn1696 as Reporter,” Microbiology 143 (1997): 1503–1512, 10.1099/00221287-143-5-1503. 9168600

[92] T. Siegl , B. Tokovenko , M. Myronovskyi , and A. Luzhetskyy , “Design, Construction and Characterisation of a Synthetic Promoter Library for Fine‐Tuned Gene Expression in Actinomycetes,” Metabolic Engineering 19 (2013): 98–106, 10.1016/j.ymben.2013.07.006. 23876413

[93] M. L. Chiu , M. Folcher , T. Katoh , et al., “Broad Spectrum Thiopeptide Recognition Specificity of the Streptomyces Lividans TipAL Protein and its Role in Regulating Gene Expression,” Journal of Biological Chemistry 274 (1999): 20578–20586, 10.1074/jbc.274.29.20578. 10400688

[94] J. Otsuka and T. Kunisawa , “Characteristic Base Sequence Patterns of Promoter and Terminator Sites in φX174 and Fd Phage DNAs,” Journal of Theoretical Biology 97 (1982): 415–436, 10.1016/0022-5193(82)90374-5. 6215541

[95] J. Brown , “The iGEM Competition: Building With Biology,” IET Synthetic Biology 1 (2007): 3–6, 10.1049/iet-stb:20079020.

[96] J. Huff , A. Czyz , R. Landick , and M. Niederweis , “Taking Phage Integration to the Next Level as a Genetic Tool for Mycobacteria,” Gene 468 (2010): 8–19, 10.1016/j.gene.2010.07.012. PMC2952446 20692326

[97] M. Niemczura , A. Nuutila , R. Wang , K. Rauhanen , S. E. Nybo , and M. Metsä‐Ketelä , “Three‐Enzyme Cascade Catalyzes Conversion of Auramycinone to Resomycin in Chartreusin Biosynthesis,” ACS Chemical Biology 20 (2025): 1457–1463, 10.1021/acschembio.5c00205. PMC12281474 40598839

