# Supplementary material for: Complete Pathway Refactoring and Combinatorial Biosynthesis of Anthracyclines for Modulation of Histone Eviction and DNA Damage Bioactivities
Source: Angew Chem Int Ed Engl. 2026 Sep 23;65(45):e1050964. doi: 10.1002/anie.1050964 (PMC13632653; doi:10.1002/anie.1050964)
Supplement: Supplementary file 1 [file ANIE-65-e1050964-s001.docx]

Supporting information for

**Complete pathway refactoring and combinatorial biosynthesis of anthracyclines for modulation of histone eviction and DNA damage bioactivities**

Rongbin Wang^[a]^, Heli Tirkkonen^[a]^, Sabina Y. van der Zanden^[b]^, Larissa Ponomareva^[c], [d]^, Isaline Fagon^[e]^, Leslie Fève^[e]^, Kendall Paige^[f]^, Courtney L. Brown^[f]^, Nora Schwartz^[f]^, Jacob Hecht^[f]^, Jennifer Nguyen^[f]^, Jon S. Thorson^[c], [d]^, Jacques Neefjes^[b]^, S. Eric Nybo*^[f]^, Khaled A. Shaaban*^[c], [d]^ and Mikko Metsä-Ketelä*^[a]^

[a]	Dr. R. Wang, H. Tirkkonen, Dr. M. Metsä-Ketelä*
Department of Life Technologies, University of Turku, FIN-20014, Finland
E-mail: mianme@utu.fi

[b]	Dr. S. Y. van der Zanden, Dr. J. Neefjes
Department of Cell and Chemical Biology (CCB) and Oncode Institute, Leiden University Medical Center (LUMC), the Netherlands

[c]	Dr. L. Ponomareva, Dr. J. S. Thorson, Dr. K. A. Shaaban*
Center for Pharmaceutical Research and Innovation, College of Pharmacy, University of Kentucky, Lexington, Kentucky 40536, United States
E-mail: Khaled_shaaban@uky.edu

[d]	Dr. L. Ponomareva, Dr. J. S. Thorson, Dr. K. A. Shaaban*
Department of Pharmaceutical Sciences, College of Pharmacy, University of Kentucky, Lexington, Kentucky 40536, United States
E-mail: Khaled_shaaban@uky.edu

[e]	I. Fagon, L. Fève
Chemistry and Chemical Engineering Department, SIGMA Clermont, Aubière, France

[f]	K. Paige, C. L. Brown, N. Schwartz, J. Hecht, J. Nguyen, Dr. S. E. Nybo*
Department of Pharmaceutical Sciences, College of Pharmacy, Ferris State University, Big Rapids, MI 49307, United States
E-mail: EricNybo@Ferris.edu

**Materials and Methods**

**Strains and culture conditions**

The *Escherichia coli* (*E. coli*) strains TOP10 and JM109 were used for gene manipulation and protein expression. *E. coli* ET12567/pUZ8002 and *E. coli* ET12567/pR9406 were used for intergeneric conjugation with *Streptomyces*^[81-82]^. *Streptomyces coelicolor* M1146, M1152, M1152*ΔmatAB*, and *Streptomyces lividans* K4−114 were used as the heterogeneous expression hosts. *Streptomyces albus* was used for glycosyltransferase complementation experiments. *E. coli* strains were cultivated at 37°C in LB broth or LB agar plates for selection. *Streptomyces* were grown at 30°C in liquid media TSB, SG-TES (Soytone 10 g/L, Glucose 20 g/L, Yeast extract 5 g/L, TES free acid 5.73 g/L, CoCl_2_ 1 mg/L), MS, and E1 for strain cultivation and compound production. For solid cultivation, MS and ISP4 agar were applied^[81]^. When screening the exconjugants from intergeneric conjugation, 10 mM MgCl_2_ was added to the plates. *Streptomyces* fermentation processes for compound production are as follows: *Streptomyces* strains from glycerol stocks were revived on the solid plates for 3-4 days. Then, the *Streptomyces* cells were pre-cultured in 50 mL of TSB or SG-TES medium for 3-4 days and finally inoculated in fresh SG-TES or E1 producing media for 5-7 days in 250-2000 mL Erlenmeyer ﬂasks. When appropriate, chloramphenicol (25 μg/mL), ampicillin (100 μg/mL), kanamycin (50 μg/mL), apramycin (50 μg/mL), viomycin (20 μg/mL), hygromycin (50 μg/mL), and nalidixic acid (25 μg/mL) were supplemented to media for the strain selection.

**Mammalian cell culture**

Mammalian cell lines for cytotoxicity [A549 (non-small cell lung) and PC3 (prostate), and HCT116 (colorectal) human cancer cell lines, Merkel cells MKL1 and MCC26] were cultivated as previously described^[83]^. A549, PC3, and HCT116 cells were obtained from ATCC (Manassas, VA). Merkel cells MKL1 and MCC26 were a gift from Dr. Isaac Brownell’s laboratory (NIH/NIAMS, Bethesda, MD). Wild-type MelJuSo (melanoma) cells were cultured in Iscove’s Modified Dulbecco’s medium (IMDM) supplemented with 8% FCS. MelJuSo cells stably expressing PAGFP-H2A were maintained in IMDM supplemented with 8% FCS and G-418^[67]^. Cell lines were maintained in a humidified atmosphere of 5% CO_2_ at 37°C and regularly tested for the absence of mycoplasma.

**Feeding experiment**

For the feeding experiment, around 200 µg of **5** or **6** was added to the cultures of engineered *Streptomyces* strains. For comparison, two control groups were included: one with the engineered strains grown under normal conditions and another with the same amount of compounds added to an empty culture.

**Microbial inhibitory assay**

The microbial inhibition assay was performed as previously described^[55]^. Briefly, *Streptomyces* strains were cultivated on MS agar plates at 30°C for 3 days to generate spore suspensions. Spore concentrations were quantified and standardized by serial dilution on MS plates. ISP4 agar medium was dispensed into 24-well plates and supplemented with increasing concentrations of anthracyclines. A 5 µL aliquot of spore suspension, containing 10^4^ CFU, was spotted onto each well, and the plates were incubated at 30°C for 5 days.

**Plasmid and strain construction**

BioBricks assembly was used to assemble the genes. 3A assembly was applied as described previously. In brief, pSB1X3 (X=C, K, A, or T) series were used as the destination plasmids to carry the cloning genes, which harbor the constitutively active Red Fluorescent Protein (RFP) construct (J04450) to allow red-white screening. pSB1X3 with a different antibiotic resistance marker from both plasmids carrying the insert genes was first selected and digested by *Eco*RI and *Pst*I. Alkaline phosphatase was added to prevent self-ligation. The upstream insert was digested by *Eco*RI and *Spe*I. The downstream insert was digested by *Xba*I and *Pst*I. Instead of gel purification, all the digested reactions were purified with PCR clean up kit to remove the restriction enzymes. The ligation and transformation were followed by the standard *E. coli* techniques. After all rounds of BioBricks assembly, the final constructs were cloned into BioBricks-compatible *Streptomyces* plasmids using the responsible restriction sites. Intergeneric conjugation was applied to transform plasmids into *Streptomyces* using the standard techniques described previously^[81]^. The plasmids and strains used in this study are listed in Table S3-S4.

**Compound extraction**

Two methods were utilized for extracting compounds from the cultural supernatant. For anthracycline aglycones, the cultural supernatant was added with the same volume of ethyl-acetate with 1% acetic acid in centrifuge tubes. The phases were separated by 5 min of centrifugation at 4,000 g after 10 min of vortexing. The ethyl-acetate phase was collected and dried with vacuum. Methanol was added to suspend the extracts for analysis. For glycosylated anthracycline compounds, the metabolites were collected from the cultural supernatant and recovered by overnight binding to 20 g/L of LXA-1180 resin at 8°C with gentle shaking. The resin was collected by discarding the medium and washed with Milli-Q water. The absorbed compounds were extracted with methanol. For large scale purification, methanol was evaporated using a rotary evaporator and the dried compounds were resuspended in minimal volume of methanol. Enzymatic reaction products were extracted with chloroform by vigorous vortexing. The organic (lower) phase was dried under vacuum, resuspended in methanol.

**Analytical HPLC method**

The extracted compounds were analyzed by UHPLC (Shimadzu Nexera LC-40 system with a diode array detector) using a Phenomenex Kinetex C18 column (2.6 μm, 100 Å, 4.6×100 mm) or HPLC (Agilent 1260 Infinity II) using a Poroshell 120 Phenyl-Hexyl Column (2.7 μm, 4.6 mm×100 mm). Method A: Solvent A: 15% CH_3_CN/0.1% HCOOH; solvent B: CH_3_CN; flow rate: 0.3 mL/min; 0-2 min, 0% B; 2-20 min, 0-40% B; 20-24 min, 100% B; 24-29 min, 0% B.

**Enzymatic reactions**

Enzymatic reactions were conducted in a 1.5 mL Eppendorf tube containing 200 µL of reaction buffer (50 mM phosphate, 50 mM NaCl, pH 7.5). Specifically, substrate 32 was added to a final concentration of 10 µM, followed by enzymes EamC and RdmB addition from our lab stock at a final concentration of 5 µM. To allow RdmB reaction, 10 µM DTT and 400 µM SAM were also added. Reactions were incubated at 30°C for 2 hours.

**DNA dye competition assay**

DNA dye competition assays were performed as previously described^[11]^. Briefly, for the PicoGreen assay, 0.5 µg/mL pUC19 plasmid DNA was incubated with PicoGreen working solution in TE buffer. Subsequently, indicated drug concentrations were added to the DNA/PicoGreen reaction and incubated for another 5 minutes at room temperature followed by measurement of the PicoGreen fluorescence using a HIDEX Sense microplate reader with excitation and emission wavelengths of 480 nm and 520 nm, respectively. Similarly, for the Hoechst33258 assay, 5 µg/mL plasmid DNA was applied with 0.5 µg/mL Hoechst33258 in water and the fluorescence was measured using excitation/emission wavelengths of 350/460 nm. For both assays, the fluorescence signals were corrected for the corresponding drug concentrations in the absence of DNA, and quantified relative to untreated controls.

**Cancer cell line viability assay**

Mammalian cell line cytotoxicity assays were accomplished in triplicate following our previously reported protocols^[67, 83-87]^. Vehicle (DMSO) was used as the negative control, and doxorubicin (**1**) and nogalamycin (**4**) were used as positive controls. Doxorubicin (**1**) was purchased from Thermo Fisher Scientific (Waltham, MA). Nogalamycin (**4**) and aklavinone (**5**) were obtained from the UK-CPRI NPs Repository (Center for Pharmaceutical Research and Innovation, University of Kentucky)

**DNA damage analysis by Western blot**

MelJuSo cells were treated with the indicated drugs for 2 h at a concentration of 10 µM. Upon treatment, cells were washed with PBS followed by lysis in sodium dodecyl sulfate (SDS)-sample buffer (2% SDS, 10% glycerol, 5% β-mercaptoethanol, 60 mM Tris-HCl pH 6.8, and 0.01% bromophenol blue). Lysates were resolved by SDS/polyacrylamide gel electrophoresis followed by Western blotting. Primary antibodies used for blotting were γH2AX (1:1,000, 05-036, Millipore) and β-actin (1:10,000, A5441, Sigma, RRID: AB_476744). Images were quantified with ImageJ and for statistical analysis GraphPad Prism 10.2.3 was used.

**Chromatin damage analysis by microscopy**

MelJuSo cells stably expressing PAGFP-H2A were seeded in 35 mm live cell imaging dishes > 24 h prior to the experiment. Photoactivation and time-lapse experiment were performed as described^[67]^. All live cell imaging experiments were analyzed by a Leica SP8 confocal microscope system, 63 × lens, equipped with a climate chamber. Loss of fluorescence from the photoactivated region after treatment with the different compounds at 10 µM for 1 h was quantified using Image J software.

**Compound purification and structure elucidation**

**General methods for compound purfication and structure elucidation**: Ultraviolet-visible (UV-Vis) spectra were taken directly from analytical HPLC runs and show relative intensities. The NMR spectra were recorded on either Bruker Avance NEO 400 MHz (Bruker BioSpin Corporation, Billerica, MA) (^1^H, 400.13 MHz; ^13^C, 100.25 MHz), Varian 500 MHz (Agilent, Santa Clara, CA, USA) (^1^H, 500 MHz; ^13^C, 125.7 MHz) spectrometers and/or the Bruker Avance NEO 600 MHz NMR (^1^H, 600.37 MHz; ^13^C, 150.96 MHz) spectrometer, equipped with triple channel TCI 5-mm cryoprobe (all spectra were processed using Bruker Topspin 4.1.4 version, and 2D spectra were apodized with QSINE or SINE window functions and zero-filled to (2048 ×1024 points). Except for compounds **27** and **28**, all the spectra were analyzed and plotted using Mnova [where *δ*-values were referenced to respective solvent signals (CD_3_OD, δ_H_ 3.31 ppm, δ_C_ 49.15 ppm; CDCl_3_, δ_H_ 7.24 ppm, δ_C_ 77.23 ppm]. For compounds **27** and **28**, the NMR spectra were recorded with 600 Mhz Bruker AVANCE-III system with liquid nitrogen cooled Prodigy TCI cryoprobe or 500 MHz Bruker AVANCE-III system with liquid nitrogen cooled Prodigy BBO cryoprobe. All NMR spectra were processed in Bruker TopSpin 4.1.3 version and the signals were internally referenced to the solvent signals or tetramethylsilane. Except for compounds **27** and **28**, all the remaining high resolution electrospray ionization (HRESI) mass spectra were recorded on AB SCIEX Triple TOF^®^ 5600 system (AB Sciex, Framingham, MA, USA). High resolution electrospray ionization mass spectra for compounds **27** and **28** were recorded on Waters Acquity RDa Detector.

HPLC-UV analyses were carried out using a Shimadzu Nexera X3 system equipped with a photodiode array detector and a Kinetex C18 column (1.7 µm, 100 Å, 100 x 2.1 mm, Phenomenex) [**Method A1:** solvent A: H_2_O/0.1% formic acid; solvent B: CH_3_CN; flow rate: 0.3 mL/min; 0-2 min, 0 % B; 2-22 min, 0-100 % B; 22-24 min, 100 % B; 24-29 min, 0 % B]. HPLC-UV/MS analyses were accomplished with an Agilent InfinityLab LC/MSD mass spectrometer (*MS* Model G6125B; Agilent Technologies, Santa Clara, CA, USA) equipped with an Agilent 1260 Infinity II Series Quaternary LC system and a Phenomenex NX-C18 column (250 × 4.6 mm, 5 *μ*m; Phenomenex, Torrance, CA) [**Method A2:** solvent A: H_2_O/0.1% formic acid, solvent B: CH_3_CN; flow rate: 0.5 mL min^-1^; 0-30 min, 5-100% B (linear gradient); 30-35 min, 100% B; 35-36 min, 100%-5% B; 36-40 min, 5% B]. HPLC-UV analyses were carried out using a Shimadzu Nexera X3 system equipped with a photodiode array detector and a Kinetex C18 column (1.7 µm, 100 Å, 100 x 2.1 mm, Phenomenex, Torrance, CA) [**Method B:** solvent A: H_2_O/0.1% formic acid, solvent B: CH_3_CN; flow rate: 0.3 mL min^-1^; 0-2 min, 0 % B; 2-22 min, 0-100 % B; 22-24 min, 100 % B; 24-29 min, 0 % B). *Semi*-preparative HPLC were carried out in a Agilent 1260 Infinity II (Prep HPLC) system equipped with a Diode Array Detector (DAD) and a Gemini 5 *μ*m C_18_ 110 Å, LC column 250 × 10 mm (Phenomenex, Torrance, CA) [**Method C1:** solvent A: H_2_O/0.025% TFA; solvent B: CH_3_CN; flow rate: 5.0 mL min^-1^; 0-3 min, 10% B; 3-15 min, 10-65% B; 15-18 min, 65-100% B; 18-20 min, 100% B; 20-21 min, 100-10% B; 21-22 min, 10% B; **Method C2:** solvent A: H_2_O/0.025% TFA; solvent B: CH_3_CN; flow rate: 5.0 mL min^-1^; 0-3 min, 15% B; 3-7 min, 15-45% B; 7-22 min, 45-55% B; 22-24 min, 55-100% B; 24-26 min, 100% B; 26-27 min, 100-15% B; 27-28 min, 15% B; **Method C3:** solvent A: H_2_O/0.025% TFA; solvent B: CH_3_CN; flow rate: 5.0 mL min^-1^; 0-3 min, 25% B; 3-7 min, 25-50% B; 7-22 min, 50-100% B; 22-26 min, 100% B; 26-27 min, 100-25% B; 27-28 min, 25% B; **Method C4:** solvent A: H_2_O/0.025% TFA; solvent B: CH_3_CN; flow rate: 5.0 mL min^-1^; 0-3 min, 15% B; 3-7 min, 15-25% B; 7-23 min, 25-55% B; 23-24 min, 55-100% B; 24-25 min, 100% B; 25-26 min, 100-25% B; 26-28 min, 15% B; **Method C5:** solvent A: H_2_O/0.025% TFA; solvent B: CH_3_CN; flow rate: 5.0 mL min^-1^; 0-3 min, 15% B; 3-22 min, 15-100% B; 22-25 min, 100% B; 25-26 min, 100-15% B; 26-28 min, 15% B; **Method D:** solvent A: H_2_O/0.025% TFA; solvent B: CH_3_CN; flow rate: 5.0 mL min^-1^; 0-3 min, 15% B; 3-8 min, 15-45% B; 8-18 min, 45-100% B; 18-20 min, 100% B; 20-21 min, 100-15% B; 21-22 min, 15% B; **Method E:** solvent A: H_2_O/0.025% TFA; solvent B: CH_3_CN; flow rate: 5.0 mL min^-1^; 0-7 min, 15% B; 7-22 min, 15-55% B; 22-24 min, 55-100% B; 24-26 min, 100% B; 26-27 min, 100-15% B; 27-28 min, 15% B; **Method F:** solvent A: H_2_O/0.1% formic acid; solvent B: CH_3_CN; flow rate: 20 mL/min; 0-2 min, 0 % B; 2-22 min, 0-100 % B; 22-24 min, 100 % B; 24-29 min, 0 % B]. All solvents used were of ACS grade and purchased from the Pharmco-AAPER (Brookfield, CT). Size exclusion chromatography was performed on Sephadex LH-20 (25-100 *μ*m; GE Healthcare, Piscataway, NJ).

**Purification of compound 19 (**along with compound **21):** The reddish-brown extract (0.183 g) from strain RW-27 fermentation (20x100 mL SG-TES culture in 250mL Erlenmeyer flask) was dissolved in MeOH followed by Sephadex LH-20 (MeOH; 2.5 x 45 cm) and yielded two fractions F1 (38 mg) and F2 (43 mg). *Semi*-prep-HPLC purification (**Method C5**) of F1 and F2 afforded compounds **19** (7-*O*-*α*-l-daunosaminyl-auramycinone; 1.45 mg) and **21** (7-*O*-*α*-l-daunosaminyl-nogalamycinone; 1.93 mg), respectively, in pure form as yellow solids.

**Purification of compound 20 (**along with compound **38):** The reddish-brown extract (0.31 g) from strain RW-23 fermentation (20x100 mL SG-TES culture in 250mL Erlenmeyer flask) was dissolved in MeOH followed by Sephadex LH-20 (MeOH; 2.5 x 45 cm) to give two fractions F1 (55 mg) and F2 (63 mg). *Semi*-prep-HPLC purification (**Method C4**) of F1 and F2 afforded compounds **20** (7-*O*-*α*-l-daunosaminyl-9-*epi*-aklavinone; 2.84 mg) and **38** (*N*-acetyl-7-*O*-*α*-l-daunosaminyl-9-*epi*-aklavinone; 3.08 mg), respectively, in pure form as yellow solids.

**Purification of compound 21:** Methanol extracts from strain RW-32 cultures (8x250mL SG-TES culture in 2 L Erlenmeyer flasks) were first purified by silica chromatography. Silica chromatography was performed using high-purity grade silica (pore size 60 Å, 230-400 mesh particle size) and a gradient elution from 99:0:1 CHCl_3_/MeOH/HCOOH to 0:99:1 CHCl_3_/MeOH/HCOOH. Fractions from silica chromatography containing compounds of interest were evaporated using a rotary evaporator and suspended in minimal volume of methanol for *semi*-preparative HPLC. *Semi*-preparative HPLC were carried out using a LC-20AP/CBM-20A system with a diode array detector (Shimadzu) and phenyl-hexyl, 5 μm, 100 Å, 250 x 21.2 mm Kinetex column (Phenomenex) [**Method F**]. Fractions containing pure compounds were dried using rotary evaporator and desiccator, and compound **21** was obtained in pure form as yellow solids.

**Purification of compound 22 (**along with compound **39):** The obtained 0.85 g reddish-brown crude extract from strain RW-24 culture (40x100 mL SG-TES culture in 250mL Erlenmeyer flask) was dissolved in MeOH (10 mL) followed by Sephadex LH-20 (MeOH; 2.5 x 45 cm) yielded two fractions F1 (120 mg) and F2 (25 mg). *Semi*-prep-HPLC purification (**Method C4**) of F1 afforded compounds **22** (7-*O*-*α*-l-acosaminyl-9-*epi*-aklavinone; 2.34 mg) and **39** (7-*O*-*α*-l-acosaminyl-aklavinone; 3.09 mg), respectively, in pure form as yellow solids. Unrelated uridine-derived metabolite namely 2′-*O*-methyl-5′-*O*-(2-methyl-1-oxobutyl) uridine was purified from F2 by *Semi*-prep-HPLC purification (**Method C4**) and was obtained as white solid (1.87 mg), a known bacterial metabolite reported previously from the coral-associated actinomycete *Pseudonocardia* sp. SCSIO 11457^[88]^.

**Purification of compound 23 (**along with compounds **6** and **35):** The reddish-brown oily crude extract (0.393 g) produced by RW-33 (8 x 250 mL in 2 L flasks, E1-medium, 5 days) was dissolved in MeOH (10 mL) followed by Sephadex LH-20 (MeOH; 2.5 x 65 cm) mentored by TLC to afford five fractions. LC-MS analysis indicates the target metabolite **23** was mainly detected in fraction F1, and compounds **6** and **35** were detected in fractions F2 and F3. *Semi*-prep-HPLC purification (**Method C1**) of F1 afforded compound **23** (7-*O*-*α*-l-acosaminyl-nogalamycinone; 2.65 mg) in pure form as yellow solid. Similarly, s*emi*-prep-HPLC purification (**Method D**) of the combined fractions F2 and F3 afforded compounds **6** (nogalamycinone; 12.24 mg) and **35** (7-deoxy-nogalamycinone; 11.10 mg) in pure forms as yellow solids.

**Purification of compounds 24**, **31 (**along with compounds **26**, **35**, **36**, and **37):** About 7.0 g of the crude extract from strain RW-49 culture (60x100 mL SG-TES culture in 250 mL Erlenmeyer flask) was dissolved in MeOH (30 mL) followed by Sephadex LH-20 (MeOH; 3.5 x 50 cm) mentored by TLC and LC-MS to afford three fractions. LC-MS analysis indicates the target glycosides were mainly detected in fraction F1 (984.5 mg), and compounds **35** and **36** were detected as minor metabolites in fractions F2 (84.1 mg) and F3 (137.9 mg). About 200 mg of F1 were dissolved in 6 mL MeOH, filtered-off, followed by *Semi*-prep-HPLC purification (**Method C2**) to afford compounds **24** (7.05 mg, yellow solid), **26** (11.55 mg, yellow solid), **31** (0.66 mg, red solid), and **37** (0.79 mg, red solid) in pure forms. Similarly, s*emi*-prep-HPLC purification (**Method C3**) of the combined fractions F2 and F3 afforded compounds **35** (7-deoxy-nogalamycinone; 2.4 mg) and **36** (7-deoxy-auramycinone; 2.4 mg) in pure forms as yellow solids.

**Purification of compound 25:** The reddish-brown extract (0.55 g) produced by strain RW-25 (12 x 250 mL in 2 L flasks, E1-medium, 5 days) was subjected for silica gel column fractionation, followed by Sephadex LH-20 (MeOH; 2.5 x 45 cm) and *prep*-HPLC purification (**Method E**) afforded compound **25** (7-*O*-*α*-l-rhodosaminyl-9-*epi*-aklavinone (9.15 mg) in pure form as yellow solid.

**Purification of compounds 26:** RW-31 was cultured and purified on the same manner as RW-32 and compound **26** (7-O-α-l-rhodosaminyl-nogalamycinone) was obtained in pure form as yellow solids.

**Purification of compounds 27-28:** RW-28 was cultured and purified on the same manner as RW-32 and compounds **27**-**28** were obtained in pure form as yellow solids [7-*O*-α-l-3′,4′-demethyl-nogalosyl-9-epi-akalvinone (**27**), 7-*O*-α-l-3′,4′-demethyl-nogalosyl-akalvinone (**28**)].

**Purification of compound 29 (**along with compounds **21** and **35):** Around 0.521 g of reddish-brown oily crude extract obtained from strain RW-50 cultivation (4 x 250 mL E1 media in 2 L flasks) was dissolved in MeOH (15 mL) followed by Sephadex LH-20 (MeOH; 2.5 x 65 cm) mentored by TLC/LCMS analysis to afford two fractions F1 and F2. LC-MS analysis indicates the target glycosylated metabolites **29** and **21** was mainly detected in fraction F1, and the aglycone **35** (7-deoxy-nogalamycinone; 26.0 mg) was the main product in fraction F2. *Semi*-prep-HPLC purification (**Method C4**) of F1 afforded compounds **21** (7-*O*-*α*-l-daunosaminyl-nogalamycinone; 3.57 mg, yellow solid) and **29** (1-hydroxy-7-*O*-*α*-l-daunosaminyl-nogalamycinone; 3.75 mg, red solid) in pure forms.

**Purification of compound 30 (**along with compounds **23** and **35):** The 0.571 g of reddish-brown oily crude extract obtained from strain RW-51 cultivation (4 x 250 mL E1 media in 2 L flasks) was dissolved in MeOH (15 mL) followed by Sephadex LH-20 (MeOH; 2.5 x 65 cm) to afford two fractions F1 and F2. *Semi*-prep-HPLC purification (**Method C4**) of F1 afforded compounds **23** (7-*O*-*α*-l-Acosaminyl-nogalamycinone; 5.12 mg, yellow solid) and **30** (1-Hydroxy-7-*O*-*α*-l-acosaminyl-nogalamycinone; 5.6 mg, red solid). LC-MS analysis fraction F2 indicates the aglycone **35** (7-deoxy-nogalamycinone; 26.0 mg) was the main product in fraction F2.

**Supplementary text**

**Structure elucidation of novel compounds 6, 19–31, and 35–39.**

The chemical structures of compounds **6**, **19–31**, and **35–39** were elucidated using high-resolution electrospray ionization mass spectrometry (HRESI-MS), in conjunction with comprehensive 1D (^1^H, ^13^C) and 2D (COSY, HSQC, HMBC, TOCSY, NOESY) NMR analyses (Figure S13-14 and Tables S5-12), and by comparison with literature data. The glycosylated compounds reported herein were classified into four different sub-classes (nogalamycinone/auramycinone and aklavinone/9-*epi*-aklavinone) along with the attachment of four different sugar residues (*α*-l-acosamine*, α*-l-daunosamine*, α*-l-rhodosamine and *α*-l-3′,4′-demethyl-nogalose).

The stereochemistry at C-9 position of the four aglycone sub-structures was mainly established based on NOESY correlations. Specifically, the multiplicity and coupling constant of H-7 (dd, *J* = 5.7, 2.0-2.2 Hz) in nogalamycinone-substructure (**21**, **23**, **26**, **29**-**31**) were different from the coupling constant of H-7 (t, *J* = 7.1~7.8 Hz) for those auramycinone-substructures (**19**, **24**, **37**). This observation was also supported by the displayed strong NOESY correlation between CH_3_-13/H-10 in compounds **21**, **23**, **26**, **29**-**31**, and the weak or not detected NOESY correlation for auramycinone substructures (**19**, **24**), Figure S14. Similarly, for the 9-*epi*-aklavinone aglycone substructures, a strong NOESY correlation was observed between CH_3_-14/H-10 in compounds **20**, **22**, **25**, **27** and **38**; however, a weak or non-detected NOESY correlation was observed between CH_3_-14/H-10 in 9-*epi*-aklavinone substructures (**28**, **39**), Figure S14. In addition, the multiplicity/coupling constant of H-7 (dd, *J* = 7.7, 7.3 Hz; t, *J* = 8.3 Hz) in aklavinone-glycosides (**28**, **39**) were different from multiplicity/coupling constant of H-7 (dd, *J* = 6.2~5.1, 2.8~1.8 Hz) of the 9-*epi*-aklavinone-glycosides (**20**, **22**, **25**, **27** and **38**; Tables S11-12).

The attachment of sugar residues at C-7 position of the aglycones were established based on the strong HMBC correlation from H-7 to C-1′ and from H-1′ to C-7. The stereochemistry of the four sugar residues were established based on the NOESY correlations/coupling constants and by comparison with literature data. Specifically, the *α*-l-acosamine and *α*-l-daunosamine/*α*-l-rhodosamine were mainly differs at C-4′ stereochemistry. In addition, *α*-l-daunosamine and *α*-l-rhodosamine were mainly differs at 3′-*N*-substitution, the 3′-NH_2_ of *α*-l-daunosamine was replaced by 3′-*N*(CH_3_)_2_ in *α*-l-rhodosamine. The H-4′ coupling constant of acosamine (t, *J* = ~9.4 Hz; established the axial positions of H-4′/H-3′/H-5′) is different from the coupling constant of H-4′ (brd, *J* = ~2.8 Hz, equatorial/axial coupling constant) daunosamine/*α*-l-rhodosamine residues. Based on these insights and by comparison of the NMR data/coupling constants of the sugar residues (Tables S9-12), NOESY correlations (Figure S14), the sugar residues were established as *α*-l-acosamine (compounds **22**-**23**, **30** and **39**), *α*-l-daunosamine (compounds **19**-**21** and **29**), and *α*-l-rhodosamine (compounds **24**-**26**, **31** and **37**). In addition, compound **38** was also established to contain the *N*-acylated-*α*-l-daunosamine moiety, confirmed by cumulative full NMR analysis (Figure S13-14). Finally, compounds **27**-**28** were established to be glycosylated with the *α*-l-3′,4′-demethoxy-nogalose residue at 7-position, by full HRMS and NMR data analyses. The stereochemistry of *α*-l-3′,4′-demethoxy-nogalose was established based on the protons coupling constants (Table S12), NOESY correlations (Figure S14), and by comparison with literature data.

Finally, the oxidation in compounds **30**, **31** and **37** were established based on (+)-HRESIMS, with Δ*m/z* =16 higher than those of 1-dexoy analogs **23**, **26** and **24**, respectively. The attachment of the hydroxy-group at C-1 position in compounds **30**, **31** and **37** was established by based on cumulative analysis of 1D (^1^H and ^13^C-NMR) and 2D (COSY, HSQC, HMBC, TOCSY and NOESY) NMR data (Tables S6 and S10, and Figure S13-14), and by comparison with the 1-dexoy analogs **23**, **26** and **24**, respectively.

**Physicochemical properties of** **compounds 6**, **19**-**31** **and** **35**-**39**.

Nogalamycinone (**6**). C_21_H_18_O_8_ (398); yellow solid; UV/vis *λ*_max_ 198, 228, 258, 290 (sh), 430 nm; ^1^H NMR (CDCl_3_, 600 MHz) and ^13^C NMR (CDCl_3_, 150 MHz), see Tables S5 and S9; (‒)**-**ESI-MS: *m/z* 397 [M – H]^‒^; (+)-ESI-MS: *m/z* 381 [(M-H_2_O) + H]^+^, 363 [(M-2H_2_O) + H]^+^; (‒)**-**HRESI-MS: *m/z* 397.0944 [M – H]^‒^ (calcd for C_21_H_17_O_8_, 397.0929).

7-*O*-*α*-l-Daunosaminyl-auramycinone (**19**). C_27_H_29_NO_10_ (527); yellow solid; UV/vis *λ*_max_ 198, 228, 258, 290 (sh), 430 nm; ^1^H NMR (CD_3_OD, 600 MHz) and ^13^C NMR (CD_3_OD, 150 MHz), see Tables S5 and S9; (‒)**-**ESI-MS: *m/z* 526 [M – H]^‒^; (+)-ESI-MS: *m/z* 528 [M + H]^+^, 1055 [2M + H]^+^; (‒)**-**HRESI-MS: *m/z* 526.1765 [M – H]^‒^ (calcd for C_27_H_28_NO_10_, 526.1719).

7-*O*-*α*-l-Daunosaminyl-9-*epi*-aklavinone (**20**). C_28_H_31_NO_10_ (541); yellow solid; UV/vis *λ*_max_ 198, 228, 258, 292 (sh), 430 nm; ^1^H NMR (CD_3_OD, 600 MHz) and ^13^C NMR (CD_3_OD, 150 MHz), see Tables S7 and S11; (‒)**-**ESI-MS: *m/z* 540 [M – H]^‒^; (+)-ESI-MS: *m/z* 542 [M + H]^+^, 1083 [2M + H]^+^; (‒)**-**HRESI-MS: *m/z* 540.1924 [M – H]^‒^ (calcd for C_28_H_30_NO_10_, 540.187520).

7-*O*-*α*-l-Daunosaminyl-nogalamycinone (**21**). C_27_H_29_NO_10_ (527); yellow solid; UV/vis *λ*_max_ 198, 228, 258, 290 (sh), 430 nm; ^1^H NMR (CD_3_OD, 600 MHz) and ^13^C NMR (CD_3_OD, 150 MHz), see Tables S5 and S9; (‒)**-**ESI-MS: *m/z* 526 [M – H]^‒^; (+)-ESI-MS: *m/z* 528 [M + H]^+^, 1055 [2M + H]^+^; (‒)**-**HRESI-MS: *m/z* 526.1769 [M – H]^‒^ (calcd for C_27_H_28_NO_10_, 526.1719).

7-*O*-*α*-l-Acosaminyl-9-*epi*-aklavinone (**22**). C_28_H_31_NO_10_ (541); yellow solid; UV/vis *λ*_max_ 198, 230, 258, 298 (sh), 430 nm; ^1^H NMR (CD_3_OD, 600 MHz) and ^13^C NMR (CD_3_OD, 150 MHz), see Tables S7 and S11; (‒)**-**ESI-MS: *m/z* 540 [M – H]^‒^; (+)-ESI-MS: *m/z* 542 [M + H]^+^, 1083 [2M + H]^+^; (‒)**-**HRESI-MS: *m/z* 540.1925 [M – H]^‒^ (calcd for C_28_H_30_NO_10_, 540.1875).

7-*O*-*α*-l-Acosaminyl-nogalamycinone (**23**). C_27_H_29_NO_10_ (527); yellow solid; UV/vis *λ*_max_ 198, 228, 258, 290 (sh), 430 nm; ^1^H NMR (CD_3_OD, 600 MHz) and ^13^C NMR (CD_3_OD, 150 MHz), see Tables S5 and S9; (‒)**-**ESI-MS: *m/z* 526 [M – H]^‒^; (+)-ESI-MS: *m/z* 528 [M + H]^+^, 1055 [2M + H]^+^; (‒)**-**HRESI-MS: *m/z* 526.1738 [M – H]^‒^ (calcd for C_27_H_28_NO_10_, 526.1719); (+)**-**HRESI-MS: *m/z* 528.1922 [M + H]^+^ (calcd for C_27_H_30_NO_10_, 528.186423), 1055.3632 [2M + H]^+^ (calcd for C_54_H_59_N_2_O_20_, 1055.3655).

7-*O*-*α*-l-Rhodosaminyl-auramycinone (**24**). C_29_H_33_NO_10_ (555); yellow solid; UV/vis *λ*_max_ 198, 228, 258, 290 (sh), 430 nm; ^1^H NMR (CD_3_OD, 600 MHz) and ^13^C NMR (CD_3_OD, 150 MHz), see Tables S6 and S10; (‒)**-**ESI-MS: *m/z* 554 [M – H]^‒^; (+)-ESI-MS: *m/z* 556 [M + H]^+^; (‒)**-**HRESI-MS: *m/z* 554.2074 [M – H]^‒^ (calcd for C_29_H_32_NO_10_, 554.2032).

7-*O*-*α*-l-Rhodosaminyl-9-*epi*-aklavinone (**25**). C_30_H_35_NO_10_ (569); yellow solid; UV/vis *λ*_max_ 198, 230, 258, 298 (sh), 430 nm; ^1^H NMR (CD_3_OD, 600 MHz) and ^13^C NMR (CD_3_OD, 150 MHz), see Tables S7 and S11; (‒)**-**ESI-MS: *m/z* 568 [M – H]^‒^; (+)-ESI-MS: *m/z* 570 [M + H]^+^; (+)**-**HRESI-MS: *m/z* 570.2377 [M + H]^+^ (calcd for C_30_H_36_NO_10_, 570.2334).

7-*O*-*α*-l-Rhodosaminyl-nogalamycinone (**26**). C_29_H_33_NO_10_ (555); yellow solid; UV/vis *λ*_max_ 198, 228, 258, 292 (sh), 423 nm; ^1^H NMR (CD_3_OD, 600 MHz) and ^13^C NMR (CD_3_OD, 150 MHz), see Tables S6 and S10; (‒)**-**ESI-MS: *m/z* 554 [M – H]^‒^; (+)-ESI-MS: *m/z* 556 [M + H]^+^; (‒)**-**HRESI-MS: *m/z* 554.2078 [M – H]^‒^ (calcd for C_29_H_32_NO_10_, 554.2032).

7-*O*-*α*-l-3′,4′-Demethyl-nogalosyl-9-*epi*-akalvinone (**27**). C_30_H_34_O_12_ (586); yellow solid; UV/vis *λ*_max_ 198, 230, 258, 298 (sh), 430 nm; ^1^H NMR (CD_3_OD, 600 MHz) and ^13^C NMR (CD_3_OD, 125 MHz), see Tables S7 and S12; (+)-HRESI-MS: *m/z* 609.1931 [M + Na]^+^ (calcd for C_30_H_34_O_12_Na, 609.1942).

7-*O*-*α*-l-3′,4′-Demethyl-nogalosyl-akalvinone (**28**). C_30_H_34_O_12_ (586); yellow solid; UV/vis *λ*_max_ 198, 230, 258, 298 (sh), 430 nm; ^1^H NMR (CD_3_OD, 600 MHz) and ^13^C NMR (CD_3_OD, 150 MHz), see Tables S7 and S12; (‒)**-**HRESI-MS: *m/z* 585.1974 [M – H]^‒^ (calcd for C_30_H_33_O_12_, 585.1977).

1-Hydroxy-7-*O*-*α*-l-daunosaminyl-nogalamycinone (**29**). C_27_H_29_NO_11_ (543); yellow solid; UV/vis *λ*_max_ 198, 230, 258, 298 (sh), 495, 530 (sh) nm; ^1^H NMR (CD_3_OD, 600 MHz) and ^13^C NMR (CD_3_OD, 150 MHz), see Tables S6 and S10; (‒)**-**ESI-MS: *m/z* 542 [M – H]^‒^; (+)-ESI-MS: *m/z* 544 [M + H]^+^, 1087 [2M + H]^+^; (‒)**-**HRESI-MS: *m/z* 542.1707 [M – H]^‒^ (calcd for C_27_H_28_NO_11_, 542.1669).

1-Hydroxy-7-*O*-*α*-l-acosaminyl-nogalamycinone (**30**). C_27_H_29_NO_11_ (543); yellow solid; UV/vis *λ*_max_ 198, 230, 258, 298 (sh), 495, 530 (sh) nm; ^1^H NMR (CD_3_OD, 600 MHz) and ^13^C NMR (CD_3_OD, 150 MHz), see Tables S6 and S10; (‒)**-**ESI-MS: *m/z* 542 [M – H]^‒^; (+)-ESI-MS: *m/z* 544 [M + H]^+^, 1087 [2M + H]^+^; (‒)**-**HRESI-MS: *m/z* 542.1714 [M – H]^‒^ (calcd for C_27_H_28_NO_11_, 542.1669).

1-Hydroxy-7-*O*-*α*-l-rhodosaminyl-nogalamycinone (**31**). C_29_H_33_NO_11_ (571); red solid; UV/vis *λ*_max_ 198, 230, 258, 298 (sh), 498, 530 (sh) nm; ^1^H NMR (CD_3_OD, 600 MHz) and ^13^C NMR (CD_3_OD, 150 MHz), see Tables S6 and S10; (‒)**-**ESI-MS: *m/z* 570 [M – H]^‒^; (+)-ESI-MS: *m/z* 572 [M + H]^+^; (‒)**-**HRESI-MS: *m/z* 570.2020 [M – H]^‒^ (calcd for C_29_H_32_NO_11_, 570.1981).

7-Deoxy-nogalamycinone (**35**). C_21_H_18_O_7_ (382); yellow solid; UV/vis *λ*_max_ 200, 228, 260, 290 (sh), 432 nm; ^1^H NMR (CDCl_3_, 600 MHz) and ^13^C NMR (CDCl_3_, 150 MHz), see Tables S5 and S9; (‒)**-**ESI-MS: *m/z* 381 [M – H]^‒^; (+)-ESI-MS: *m/z* 383 [M + H]^+^, 365 [(M-H_2_O) + H]^+^, 787 [M+Na]^+^; (‒)**-**HRESI-MS: *m/z* 381.0960 [M – H]^‒^ (calcd for C_21_H_17_O_7_, 381.0979).

7-Deoxy-auramycinone (**36**). C_21_H_18_O_7_ (382); yellow solid; UV/vis *λ*_max_ 200, 228, 260, 290 (sh), 432 nm; ^1^H NMR (CDCl_3_, 600 MHz) and ^13^C NMR (CDCl_3_, 150 MHz), see Tables S5 and S9; (‒)**-**ESI-MS: *m/z* 381 [M – H]^‒^; (+)-ESI-MS: *m/z* 383 [M + H]^+^; (‒)**-**HRESI-MS: *m/z* 381.1019 [M – H]^‒^ (calcd for C_21_H_17_O_7_, 381.0979).

1-Hydroxy-7-*O*-*α*-l-rhodosaminyl-auramycinone (**37**). C_29_H_33_NO_11_ (571); red solid; UV/vis *λ*_max_ 198, 228, 258, 295 (sh), 495, 530 (sh) nm; ^1^H NMR (CD_3_OD, 600 MHz) and ^13^C NMR (CD_3_OD, 150 MHz), see Tables S6 and S10; (‒)**-**ESI-MS: *m/z* 570 [M – H]^‒^; (+)-ESI-MS: *m/z* 572 [M + H]^+^; (‒)**-**HRESI-MS: *m/z* 570.2025 [M – H]^‒^ (calcd for C_29_H_32_NO_11_, 570.1981).

*N*-Acetyl-7-*O*-*α*-l-daunosaminyl-9-*epi*-aklavinone (**38**). C_30_H_33_NO_11_ (583); yellow solid; UV/vis *λ*_max_ 198, 228, 258, 290 (sh), 432 nm; ^1^H NMR (CD_3_OD, 600 MHz) and ^13^C NMR (CD_3_OD, 150 MHz), see Tables S8 and S12; (‒)**-**ESI-MS: *m/z* 582 [M – H]^‒^; (+)-ESI-MS: *m/z* 377 [(M-sugar/-2H_2_O) + H]^+^; (‒)**-**HRESI-MS: *m/z* 582.2021 [M – H]^‒^ (calcd for C_30_H_32_NO_11_, 582.1981).

7-*O*-*α*-l-Acosaminyl-aklavinone (**39**). C_28_H_31_NO_10_ (541); yellow solid; UV/vis *λ*_max_ 198, 230, 258, 298 (sh), 430 nm; ^1^H NMR (CD_3_OD, 600 MHz) and ^13^C NMR (CD_3_OD, 150 MHz), see Tables S8 and S12; (‒)**-**ESI-MS: *m/z* 540 [M – H]^‒^; (+)-ESI-MS: *m/z* 542 [M + H]^+^, 1083 [2M + H]^+^; (‒)**-**HRESI-MS: *m/z* 540.1914 [M – H]^‒^ (calcd for C_28_H_30_NO_10_, 540.1875).

**Supplementary tables**

**Table S1**. List of BioBricks compatible *Streptomyces* plasmids

| **Plasmid** | **Resistance in *Streptomyces*** | **Integrase** | **Reference** |
| --- | --- | --- | --- |
| pSET152BB | Apramycin | ΦC31 | ^[21]^ |
| pSET154BB | Apramycin | ΦC31 | ^[21]^ |
| pOSV821 | Apramycin | ΦC31 | ^[30]^ |
| pOSV807 | Hygromycin | pSAM2 | ^[36]^ |
| pOSV808 | Hygromycin | VWB | ^[36]^ |
| pOSV811 | Kanamycin | pSAM2 | ^[36]^ |
| pOSV820* | Kanamycin | pSAM2 | This study |
| pENTG1 | Viomycin | ΦTG1 | ^[36]^ |
| pENTG3 | Viomycin | ΦTG1 | ^[21]^ |
| pENSV1 | Streptomycin | SV1 | ^[35]^ |

*pOSV811 with 5′-ECK120033736 and 3′- ECK120033737 terminators added.

**Table S2**. List of promoters and terminators on BioBricks platform

| **Promoters** | **Reference** |
| --- | --- |
| *kasOp** | ^[89]^ |
| *ermEp** | ^[90]^ |
| *gapdhp*(EL) | ^[91]^ |
| *rpsLp*(CF) | ^[24]^ |
| *SF14* | ^[91]^ |
| *p21* | ^[92]^ |
| *p15* | ^[93]^ |
| *sp41* | ^[24]^ |
| *sp42* | ^[24]^ |
| *sp44* | ^[24]^ |
| *sp44-riboJ* | ^[30]^ |
| *sp41-ItsvJ* | ^[30]^ |
| *sp42-vtmoJ* | ^[30]^ |
| **Terminators** | **Reference** |
| fd phage | ^[94]^ |
| B0015 | ^[95]^ |
| B0017 | ^[95]^ |
| tt-sbi-A | ^[96]^ |

**Table S3**. List of constructed plasmids used in this work

| **No.** | **Plasmid name** | **Backbone** | **SBOL diagram** | **Reference** |
| --- | --- | --- | --- | --- |
| **Aglycone** | | | | |
| 1 | pRW10000 | pSET154BB |  | ^[21]^ |
| 2 | pAKV7 | pSET154BB |  | This study |
| 3 | pEN10001 | pSET154BB |  | ^[21]^ |
| 4 | pEAKV2 | pOSV821 |  | ^[30]^ |
| 5 | pAURA2 | pOSV821 |  | ^[97]^ |
| **TDP-carbohydrate** | | | | |
| 6 | pDAU | pOSV820 |  | This study |
| 7 | pRHO | pOSV820 |  | This study |
| 8 | pACO | pOSV820 |  | This study |
| 9 | pDENOG | pOSV820 |  | This study |
| 10 | pRHO_DEFUC_RHODI | pOSV820 |  | This study |
| 11 | pRHO_DENOG | pOSV820 |  | This study |
| **Resistance and/or GT** | | | | |
| 12 | pR_SnogEN | pOSV808 |  | This study |
| 13 | pR_SnogEND | pOSV808 |  | This study |
| 14 | pR_DnrQS | pOSV808 |  | This study |
| 15 | pR_AknST | pOSV808 |  | This study |
| 16 | pR_AknSTK | pOSV808 |  | This study |
| 17 | pSnogEN | pOSV808 |  | This study |
| **Post tailoring** | | | | |
| 18 | pF | pENTG3 |  | This study |
| 19 | pECB | pENTG3 |  | This study |
| 20 | p15_16 | pENTG3 |  | This study |
| 21 | pFP | pENSV3 |  | This study |
| 22 | pFPK | pENSV3 |  | This study |
| 23 | pFP_K | pENSV3 |  | This study |
| 24 | pFP_K_VA | pENTG3 |  | This study |
| 25 | pFP_K_VA_x4_r3 | pENTG3 |  | This study |
| 26 | pFP_K_VA_K | pENTG3 |  | This study |
| 27 | pFP_K_VA_x4_r3_K | pENTG3 |  | This study |
| 28 | pL2W_KNT_LM | pENTG3 |  | This study |

**Table S4**. List of *Streptomyces* strains used in this work

| **Strain** | **Parental strain** | **Genotype and relevant characteristics** | **Note** | **Reference** |
| --- | --- | --- | --- | --- |
| M1152∆matAB | *S. coelicolor*  M1152 | SCP1- SCP2- Δ*act* Δ*red* Δ*cpk* Δ*cda* *rpoB*(C1298T) *Δsco2961−2962* | chassis | ^[21]^ |
| *Δ*sno*gE+snogE* | *S.albus/Δ*sno*gE* | *S.albus/Δ*sno*gE*::pSnogEN | snogE complementation | This study |
| *Δ*sno*gN+snogN* | *S.albus/Δ*sno*gN* | *S.albus/Δ*sno*gN*::pSnogEN | snogN complementation | This study |
| AKV1 | M1152∆matAB | M1152∆matAB::pEN10001 | Producer of **5** | ^[21]^ |
| AKV7 | M1152∆matAB | M1152∆matAB::pAKV7 | Producer of **5** | This study |
| EAKV2 | M1152∆matAB | M1152∆matAB::pEAKV2 | Producer of **8** | ^[30]^ |
| AURA2 | M1152∆matAB | M1152∆matAB::pAURA2 | Producer of **7** | ^[30]^ |
| NOG | M1152∆matAB | M1152∆matAB::pRW10000 | Producer of **6** | ^[21]^ |
| AknST | M1152∆matAB | M1152∆matAB:: pR_AknST | R+GT(AknST) | This study |
| SnogEN | M1152∆matAB | M1152∆matAB:: pR_SnogEN | R+GT(SnogEN) | This study |
| AknST+RHO | AknST | M1152∆matAB::pR_AknST::pRHO | R+GT(AknST)+RHO | This study |
| SnogEN+DENOG | SnogEN | M1152∆matAB::pR_SnogEN::pDENOG | R+GT(SnogEN)+DENOG | This study |
| RW-1 | AKV1 | M1152∆matAB::pEN10001::pR_DnrQS | **5**+R+GT(DnrQS) | This study |
| RW-2 | AKV7 | M1152∆matAB::pAKV7::pR_DnrQS | **5**+R+GT(DnrQS) | This study |
| RW-3 | AKV1 | M1152∆matAB::pEN10001::pR_AknST | **5**+R+GT(AknST) | This study |
| RW-4 | AKV7 | M1152∆matAB::pAKV7::pR_AknST | **5**+R+GT(AknST) | This study |
| RW-5 | AKV1 | M1152∆matAB::pEN10001::pR_AknSTK | **5**+R+GT(AknSTK) | This study |
| RW-6 | AKV7 | M1152∆matAB::pAKV7::pR_AknSTK | **5**+R+GT(AknSTK) | This study |
| RW-7 | AKV1 | M1152∆matAB::pEN10001::pR_SnogEN | **5**+R+GT(SnogEN) | This study |
| RW-8 | EAKV2 | M1152∆matAB::pEAKV2::pR_DnrQS | **8**+R+GT(DnrQS) | This study |
| RW-9 | EAKV2 | M1152∆matAB::pEAKV2::pR_AknST | **8**+R+GT(AknST) | This study |
| RW-10 | EAKV2 | M1152∆matAB::pEAKV2::pR_SnogEN | **8**+R+GT(SnogEN) | This study |
| RW-11 | AURA | M1152∆matAB::pAURA2::pR_DnrQS | **7**+R+GT(DnrQS) | This study |
| RW-12 | AURA | M1152∆matAB::pAURA2::pR_AknST | **7**+R+GT(AknST) | This study |
| RW-13 | pNOG | M1152∆matAB::pRW10000::pR_SnogEN | **7**+R+GT(SnogEN) | This study |
| RW-14 | pNOG | M1152∆matAB::pRW10000::pR_SnogEND | **6**+R+GT(SnogEND) | This study |
| RW-15 | pNOG | M1152∆matAB::pRW10000::pR_AknST | **6**+R+GT(AknST) | This study |
| RW-16 | pNOG | M1152∆matAB::pRW10000::pR_DnrQS | **6**+R+GT(DnrQS) | This study |
| RW-17 | RW-1 | M1152∆matAB::pEN10001::pR_DnrQS::pDAU | **12** | This study |
| RW-18 | RW-2 | M1152∆matAB::pAKV7::pR_DnrQS::pDAU | **12** | This study |
| RW-19 | RW-3 | M1152∆matAB::pEN10001::pR_AknST::pRHO | **10** | This study |
| RW-20 | RW-5 | M1152∆matAB::pEN10001::pR_AknSTK:: pRHO_DEFUC_RHODI | **18** | This study |
| RW-21 | RW-6 | M1152∆matAB::pAKV7::pR_AknSTK:: pRHO_DEFUC_RHODI | **18** | This study |
| RW-22 | RW-7 | M1152∆matAB::pEN10001::pR_SnogEN::pDENOG | **28** | This study |
| RW-23 | RW-8 | M1152∆matAB::pEAKV2::pR_DnrQS::pDAU | **20** | This study |
| RW-24 | RW-8 | M1152∆matAB::pEAKV2::pR_DnrQS::pACO | **22** | This study |
| RW-25 | RW-9 | M1152∆matAB::pEAKV2::pR_AknST::pRHO | **25** | This study |
| RW-26 | RW-10 | M1152∆matAB::pEAKV2::pR_SnogEN::pDENOG | **27** | This study |
| RW-27 | RW-11 | M1152∆matAB::pAURA2::pR_DnrQS::pDAU | **19** | This study |
| RW-28 | RW-12 | M1152∆matAB::pAURA2::pR_AknST::pRHO | **24** | This study |
| RW-29 | RW-13 | M1152∆matAB::pRW10000::pR_SnogEN::pDENOG | **11** | This study |
| RW-30 | RW-14 | M1152∆matAB::pRW10000::pR_SnogEND::pRHO_DENOG | **11** | This study |
| RW-31 | RW-15 | M1152∆matAB::pRW10000::pR_AknST::pRHO | **26** | This study |
| RW-32 | RW-16 | M1152∆matAB::pRW10000::pR_DnrQS::pDAU | **21** | This study |
| RW-33 | RW-16 | M1152∆matAB::pRW10000::pR_DnrQS::pACO | **23** | This study |
| RW-34 | RW-17 | M1152∆matAB::pEN10001::pR_DnrQS::pDAU::pFP | **13** | This study |
| RW-35 | RW-17 | M1152∆matAB::pEN10001::pR_DnrQS::pDAU::pFPK | **14** | This study |
| RW-36 | RW-17 | M1152∆matAB::pEN10001::pR_DnrQS::pDAU::pFP_K | **14** | This study |
| RW-37 | RW-17 | M1152∆matAB::pEN10001::pR_DnrQS::pDAU::pFP_K_VA | **1**+**15** | This study |
| RW-38 | RW-17 | M1152∆matAB::pEN10001::pR_DnrQS::pDAU::pFP_K_VA_x4_r3 | **1**+**15** | This study |
| RW-39 | RW-18 | M1152∆matAB::pAKV7::pR_DnrQS::pDAU::pFP_K_VA | **9**+**15** | This study |
| RW-40 | RW-18 | M1152∆matAB::pAKV7::pR_DnrQS::pDAU::pFP_K_VA_x4_r3 | **1**+**15**+**16**+**17** | This study |
| RW-41 | RW-17 | M1152∆matAB::pEN10001::pR_DnrQS::pDAU::pFP_K_VA_K | **1** | This study |
| RW-42 | RW-17 | M1152∆matAB::pEN10001::pR_DnrQS::pDAU::pFP_K_VA_x4_r3_K | **1** | This study |
| RW-43 | RW-18 | M1152∆matAB::pAKV7::pR_DnrQS::pDAU::pFP_K_VA_K | **16**+**17** | This study |
| RW-44 | RW-18 | M1152∆matAB::pAKV7::pR_DnrQS::pDAU::pFP_K_VA_x4_r3_K | **1**+**16**+**17** | This study |
| RW-45 | RW-19 | M1152∆matAB::pEN10001::pR_AknST::pRHO::pF | **32** | This study |
| RW-46 | RW-19 | M1152∆matAB::pEN10001::pR_AknST::pRHO::pECB | **33** | This study |
| RW-47 | RW-19 | M1152∆matAB::pEN10001::pR_AknST::pRHO pFP_K_VA_x4_r3 | **34** | This study |
| RW-48 | RW-29 | M1152∆matAB::pRW10000::pR_SnogEND::pDENOG::pL2W_KNT_LM | **4** | This study |
| RW-49 | RW-31 | M1152∆matAB::pRW10000::pR_AknST::pRHO::p15_16 | **31** | This study |
| RW-50 | RW-32 | M1152∆matAB::pRW10000::pR_DnrQS::pDAU::p15_16 | **29** | This study |
| RW-51 | RW-33 | M1152∆matAB::pRW10000::pR_DnrQS::pACO::p15_16 | **30** | This study |

**Table S5.** ^13^C (150 MHz) NMR Spectroscopic Data of **6**, **35**-**36**, **19**, **21** and **23** (*δ* in ppm).

| Position | **6** ^a)^ | **35** ^a)^ | **36** ^a)^ | **19** ^b)^ | **21** ^b)^ | **23** ^b)^ |
| --- | --- | --- | --- | --- | --- | --- |
|  | *δ*_C_, type | *δ*_C_, type | *δ*_C_, type | *δ*_C_, type | *δ*_C_, type | *δ*_C_, type |
| 1 | 120.5, CH | 120.2, CH | 120.2, CH | 121.1, CH | 121.1, CH | 121.1, CH |
| 2 | 137.8, CH | 137.4, CH | 137.3, CH | 138.8, CH | 138.8, CH | 138.8, CH |
| 3 | 125.1, CH | 124.8, CH | 124.8, CH | 125.9, CH | 125.9, CH | 125.9, CH |
| 4 | 162.9, C | 162.7, C | 162.7, C | 163.9, C | 163.9, C | 163.9, C |
| 4a | 115.9, C | 116.2, C | 116.2, C | 117.3, C | 117.3, C | 117.3, C |
| 5 | 193.1, C | 193.1, C | 193.1, C | 194.4, C | 194.3, C | 194.3, C |
| 5a | 114.9, C | 113.9, C | 113.8, C | 116.0, C | 116.2, C | 116.2, C |
| 6 | 161.2, C | 161.1, C | 161.2, C | 163.8, C | 163.5, C | 163.5, C |
| 6a | 134.4, C | 133.7, C | 133.8, C | 132.8, C | 132.7, C | 132.7, C |
| 7 | 64.4, CH | 21.7, CH_2_ | 20.4, CH_2_ | 73.8, CH | 73.7, CH | 73.9, CH |
| 8 | 41.1, CH_2_ | 32.5, CH_2_ | 31.1, CH_2_ | 41.0, CH_2_ | 39.7, CH_2_ | 39.8, CH_2_ |
| 9 | 70.8, C | 69.6, C | 69.9, C | 71.3, C | 70.3, C | 70.3, C |
| 10 | 56.2, CH | 56.2, CH | 57.7, CH | 58.8, CH | 59.3, CH | 59.3, CH |
| 10a | 142.3, C | 142.3, C | 142.2, C | 145.1, C | 144.7, C | 144.8, C |
| 11 | 120.2, CH | 120.7, CH | 121.4, CH | 121.5, CH | 121.1, CH | 121.2, CH |
| 11a | 132.5, C | 131.1, C | 131.0, C | 134.2, C | 134.3, C | 134.4, C |
| 12 | 181.4, C | 181.9, C | 181.9, C | 182.7, C | 182.6, C | 182.6, C |
| 12a | 133.7, C | 134.0, C | 134.0, C | 135.1, C | 135.1, C | 135.1, C |
| 13 | 28.9, CH_3_ | 27.7, CH_3_ | 27.7, CH_3_ | 28.3, CH_3_ | 29.5, CH_3_ | 29.6, CH_3_ |
| 14 | 173.4, C | 173.4, C | 171.9, C | 173.4, C | 172.9, C | 172.9, C |
| 15 | 53.0, CH_3_ | 52.9, CH_3_ | 52.8, CH_3_ | 52.9, CH_3_ | 52.9, CH_3_ | 52.9, CH_3_ |
| 1′ |  |  |  | 100.9, CH | 101.4, CH | 101.0, CH |
| 2′ |  |  |  | 29.5, CH_2_ | 29.7, CH_2_ | 35.6, CH_2_ |
| 3′ |  |  |  | 48.8, CH | 49.0, CH | 51.7, CH |
| 4′ |  |  |  | 68.1, CH | 68.4, CH | 74.5, CH |
| 5′ |  |  |  | 68.2, CH | 68.4, CH | 71.0, CH |
| 6′ |  |  |  | 17.1, CH_3_ | 17.1, CH_3_ | 17.9, CH_3_ |

a) CDCl_3_; b) CD_3_OD; See Supplementary Information for NMR spectra. Assignments supported by 2D HSQC and HMBC experiments.

**Table S6.** ^13^C (150 MHz) NMR Spectroscopic Data of **24**, **26**, **29**-**31** and **37** (*δ* in ppm).

| Position | **24** ^a)^ | **26** ^a)^ | **29** ^a)^ | **30** ^a)^ | **31** ^a)^ | **37** ^a)^ |
| --- | --- | --- | --- | --- | --- | --- |
|  | *δ*_C_, type | *δ*_C_, type | *δ*_C_, type | *δ*_C_, type | *δ*_C_, type | *δ*_C_, type |
| 1 | 121.0, CH | 121.0, CH | 159.5, C | 159.5, C | 159.1*, C | 159.4*, C |
| 2 | 138.8, CH | 138.8, CH | 131.2, CH | 131.2, CH | 131.1, CH | 130.9, CH |
| 3 | 125.9, CH | 125.9, CH | 131.0, CH | 130.9, CH | 131.1, CH | 130.9, CH |
| 4 | 163.8, C | 163.8, C | 159.0, C | 159.0, C | 159.1, C | 159.4, C |
| 4a | 117.2, C | 117.2, C | 113.9, C | 113.8, C | 113.9*, C | 113.8, C |
| 5 | 194.3, C | 194.1, C | 192.3, C | 192.2, C | 192.0, C | 192.3, C |
| 5a | 115.9, C | 116.1, C | 116.3, C | 116.2, C | 116.3*, C | 116.2*, C |
| 6 | 163.7, C | 163.5, C | 163.6, C | 163.6, C | 163.4*, C | 163.3, C |
| 6a | 133.8, C | 132.6, C | 132.9, C | 132.8, C | 133.9, C | 133.9, C |
| 7 | 74.0, CH | 73.9, CH | 73.7, CH | 73.9, CH | 73.9, CH | 73.9, CH |
| 8 | 41.0, CH_2_ | 39.6, CH_2_ | 39.7, CH_2_ | 39.8, CH_2_ | 39.7, CH_2_ | 40.9, CH_2_ |
| 9 | 71.8, C | 70.3, C | 70.3, C | 70.3, C | 70.3, C | 71.8, C |
| 10 | 58.8, CH | 59.3, CH | 59.3, CH | 59.3, CH | 59.3, CH | 58.8, CH |
| 10a | 145.0, C | 144.7, C | 144.6, C | 144.7, C | 144.6*, C | 144.9*, C |
| 11 | 121.5, CH | 121.1, CH | 120.9, CH | 120.8, CH | 120.7, CH | 121.1, CH |
| 11a | 133.9, C | 134.2, C | 134.2, C | 134.2, C | 133.0*, C | 133.0*, C |
| 12 | 182.7, C | 182.5, C | 187.3, C | 187.2, C | 187.3*, C | 187.4, C |
| 12a | 135.0, C | 134.9, C | 114.0, C | 113.9, C | 113.9*, C | 113.8, C |
| 13 | 28.1, CH_3_ | 29.6, CH_3_ | 29.5, CH_3_ | 29.5, CH_3_ | 29.5, CH_3_ | 27.9, CH_3_ |
| 14 | 173.2, C | 172.9, C | 172.9, C | 172.9, C | 173.0, C | 173.2, C |
| 15 | 53.1, CH_3_ | 52.9, CH_3_ | 52.9, CH_3_ | 52.9, CH_3_ | 52.9, CH_3_ | 53.2, CH_3_ |
| 1′ | 100.9, CH | 101.4, CH |  |  | 101.4, CH | 100.9, CH |
| 2′ | 27.9, CH_2_ | 28.1, CH_2_ | 101.5, CH | 101.0, CH | 28.1, CH_2_ | 28.1, CH_2_ |
| 2′-OCH_3_ |  |  | 29.7, CH_2_ | 35.6, CH_2_ |  |  |
| 3′ | 64.0, CH | 64.0, CH |  |  | 64.0, CH | 63.9, CH |
| 3′-CH_3_ |  |  | 49.0, CH | 51.7, CH |  |  |
| 3′-N(CH_3_)_2_ | 41.7, CH_3_  40.9, CH_3_ | 41.7, CH_3_  40.9, CH_3_ |  |  | 41.6, CH_3_  40.7, CH_3_ | 41.7, CH_3_  40.9, CH_3_ |
| 4′ | 65.9, CH | 65.9, CH |  |  | 66.0, CH | 65.9, CH |
| 5′ | 68.1, CH | 68.3, CH | 68.1, CH | 74.5, CH | 68.3, CH | 68.1, CH |
| 6′ | 17.1, CH_3_ | 17.1, CH_3_ | 68.4, CH | 71.0, CH | 17.0, CH_3_ | 17.1, CH_3_ |
|  |  |  | 17.1, CH_3_ | 17.9, CH_3_ |  |  |

a) CD_3_OD; See Supplementary Information for NMR spectra. Assignments supported by 2D HSQC and HMBC experiments. *Carbon shifts obtained from HMBC correlation spectrum.

**Table S7.** ^13^C NMR Spectroscopic Data of **20**, **22**, **25** and **27**-**28** (*δ* in ppm).

| Position | **20** ^a,b)^ | **22** ^a,b)^ | **25** ^a,b)^ | **27** ^a,b)^ | **28** ^a,c)^ |
| --- | --- | --- | --- | --- | --- |
|  | *δ*_C_, type | *δ*_C_, type | *δ*_C_, type | *δ*_C_, type | *δ*_C_, type |
| 1 | 121.0, CH | 121.0, CH | 121.0, CH | 120.9, CH | 120.9, CH |
| 2 | 138.8, CH | 138.8, CH | 138.8, CH | 138.6, CH | 138.6, CH |
| 3 | 125.9, CH | 125.9, CH | 125.9, CH | 125.7, CH | 125.7, CH |
| 4 | 163.9, C | 163.8, C | 163.8, C | 163.7, C | 163.7, C |
| 4a | 117.3, C | 117.2, C | 117.1, C | 117.3, C | 117.2, C |
| 5 | 194.3, C | 194.2, C | 194.1, C | 194.3, C | 194.3, C |
| 5a | 116.2, C | 116.1, C | 116.1, C | 116.1, C | 116.0, C |
| 6 | 163.4, C | 163.4, C | 163.4, C | 163.7, C | 163.7, C |
| 6a | 133.0, C | 133.0, C | 132.9, C | 132.6, C | 133.1, C |
| 7 | 73.1, CH | 73.3, CH | 73.6, CH | 73.1, CH | 73.8, CH |
| 8 | 38.5, CH_2_ | 38.7, CH_2_ | 38.8, CH_2_ | 36.4, CH_2_ | 37.8, CH_2_ |
| 9 | 72.0, C | 72.5, C | 72.6, C | 71.9, C | 73.3, C |
| 10 | 56.6, CH | 56.5, CH | 56.3, CH | 58.4, CH | 58.0, CH |
| 10a | 144.8, C | 144.9, C | 144.8, C | 144.1, C | 145.0, C |
| 11 | 121.0, CH | 121.1, CH | 121.0, CH | 121.4, CH | 121.3, CH |
| 11a | 134.3, C | 134.2, C | 134.2, C | 134.3, C | 134.1, C |
| 12 | 182.7, C | 182.5, C | 182.5, C | 182.6, C | 182.5, C |
| 12a | 135.1, C | 135.0, C | 134.9, C | 135.1, C | 135.0, C |
| 13 | 33.8, CH_2_ | 33.8, CH_2_ | 33.8, CH_2_ | 33.2, CH_2_ | 32.9, CH_2_ |
| 14 | 8.0, CH_3_ | 8.1, CH_3_ | 8.0, CH_3_ | 7.1, CH_3_ | 7.5, CH_3_ |
| 15 | 173.2, C | 173.2, C | 173.3, C | 172.8, C | 173.0, C |
| 16 | 52.9, CH_3_ | 53.0, CH_3_ | 53.0, CH_3_ | 53.0, CH_3_ | 52.8, CH_3_ |
| 1′ | 100.9, CH | 100.5, CH | 101.1, CH | 103.2, CH | 102.0, CH |
| 2′ | 29.5, CH_2_ | 35.5, CH_2_ | 27.9, CH_2_ | 86.6, CH | 86.4, CH |
| 2′-OCH_3_ |  |  |  | 59.6, CH_3_ | 59.6, CH_3_ |
| 3′ | 49.0, CH | 51.6, CH | 64.0, CH | 73.6, C | 73.7, C |
| 3′-CH_3_ |  |  |  | 19.1, CH_3_ | 19.1, CH_3_ |
| 3′-N(CH_3_)_2_ |  |  | 41.7, CH_3_  40.9, CH_3_ |  |  |
| 4′ | 68.1, CH | 74.7, CH | 65.8, CH | 76.8, CH | 76.7, CH |
| 5′ | 68.5, CH | 71.1, CH | 68.3, CH | 69.6, CH | 69.6, CH |
| 6′ | 17.9, CH_3_ | 18.0, CH_3_ | 17.1, CH_3_ | 18.5, CH_3_ | 18.5, CH_3_ |

a) CD_3_OD; b) 150 MHz; c) 125 MHz; See Supplementary Information for NMR spectra. Assignments supported by 2D HSQC and HMBC experiments.

**Table S8.** ^13^C (150 MHz) NMR Spectroscopic Data of **38** and **39** (*δ* in ppm).

| Position | **38** ^a)^ | **39** ^a)^ |
| --- | --- | --- |
|  | *δ*_C_, type | *δ*_C_, type |
| 1 | 120.9, CH | 121.0, CH |
| 2 | 138.6, CH | 138.8, CH |
| 3 | 125.8, CH | 125.9, CH |
| 4 | 163.8, C | 163.8, C |
| 4a | 117.3, C | 117.3, C |
| 5 | 194.2, C | 194.5, C |
| 5a | 116.1, C | 116.0, C |
| 6 | 163.5, C | 163.8, C |
| 6a | 133.4, C | 134.3, C |
| 7 | 72.8, CH | 74.2, CH |
| 8 | 38.8, CH_2_ | 38.6, CH_2_ |
| 9 | 72.7, C | 74.2, C |
| 10 | 56.5, CH | 57.1, CH |
| 10a | 144.7, C | 145.2, C |
| 11 | 121.0, CH | 121.6, CH |
| 11a | 134.1, C | 133.9, C |
| 12 | 182.7, C | 182.7, C |
| 12a | 135.1, C | 135.1, C |
| 13 | 33.8, CH_2_ | 34.1, CH_2_ |
| 14 | 8.0, CH_3_ | 7.1, CH_3_ |
| 15 | 173.4, C | 172.8, C |
| 16 | 52.9, CH_3_ | 53.2, CH_3_ |
| 1′ | 102.0, CH | 100.4, CH |
| 2′ | 30.6, CH_2_ | 35.4, CH_2_ |
| 2′-OCH_3_ |  |  |
| 3′ | 47.4, CH | 51.8, CH |
| 3′-CH_3_ |  |  |
| 3′-N(CH_3_)_2_ |  |  |
| 3′-N-COCH_3_ | 172.8, C |  |
| 3′-N-COCH_3_ | 22.8, CH_3_ |  |
| 4′ | 70.1, CH | 74.6, CH |
| 5′ | 68.9, CH | 70.6, CH |
| 6′ | 17.5, CH_3_ | 17.9, CH_3_ |

a) CD_3_OD; See Supplementary Information for NMR spectra. Assignments supported by 2D HSQC and HMBC experiments.

**Table S9.** ^1^H (600 MHz) NMR Spectroscopic Data of **6**, **35**-**36**, **19**, **21** and **23** (*δ* in ppm).

| Position | **6** ^a)^ | **35** ^a)^ | **36** ^a)^ | **19** ^b)^ | **21** ^b)^ | **23** ^b)^ |
| --- | --- | --- | --- | --- | --- | --- |
|  | *δ*_H_ (mult, *J* in [Hz]) | *δ*_H_ (mult, *J* in [Hz]) | *δ*_H_ (mult, *J* in [Hz]) | *δ*_H_ (mult, *J* in [Hz]) | *δ*_H_ (mult, *J* in [Hz]) | *δ*_H_ (mult, *J* in [Hz]) |
| 1 | 7.80 (dd, 7.5, 1.1) | 7.80 (dd, 7.5, 1.2) | 7.80 (dd, 7.5, 1.2) | 7.82 (dd, 7.5, 1.3) | 7.80 (dd, 7.5, 1.4) | 7.80 (dd, 7.4, 1.2) |
| 2 | 7.68 (dd, 8.4, 7.4) | 7.66 (dd, 8.5, 7.5) | 7.65 (dd, 8.3, 7.5) | 7.78 (dd, 8.2, 7.5) | 7.77 (dd, 8.2, 7.4) | 7.77 (t, 7.8) |
| 3 | 7.30 (dd, 8.4, 1.2) | 7.28 (dd, 8.4, 1.2) | 7.27 (dd, 8.4, 1.1) | 7.36 (dd, 8.2, 1.4) | 7.35 (dd, 8.1, 1.4) | 7.34 (dd, 8.0, 1.3) |
| 4-OH | 11.91 (s) | 12.06 (s) | 12.08 (s) |  |  |  |
| 6-OH | 12.83 (s) | 12.45 (s) | 12.49 (s) |  |  |  |
| 7 | 5.39 (t, 7.0) | 3.09 (dtd, 19.1, 6.7, 1.1)  2.81 (dt, 19.1, 6.7) | 3.03 (ddd, 19.1, 6.7, 3.0)  2.86 (ddd, 19.0, 10.3, 6.8) | 5.03 (t, 7.1) | 5.17 (dd, 5.7, 2.1) | 5.17 (dd, 5.7, 2.0) |
| 8 | 2.65 (dd, 13.8, 7.1)  1.87 (dd, 13.9, 7.0) | 2.31 (dt, 13.4, 6.7)  1.90 (dtd, 13.4, 6.7, 1.1) | 2.30 (ddd, 13.8, 10.2, 6.7)  1.90 (dddd, 13.9, 6.9, 3.0, 1.6) | 2.64 (dd, 13.4, 7.2)  2.26 (ddd, 13.3, 7.1, 1.4) | 2.76 (dd, 14.3, 5.7)  2.11 (dt, 14.4, 2.1) | 2.77 (dd, 14.3, 5.6)  2.13 (brd, 14.2) |
| 10 | 3.92 (s) | 3.85 (s) | 3.89 (s) | 4.00 (s) | 4.07 (s) | 4.08 (s) |
| 11 | 7.54 (brs) | 7.57 (brs) | 7.62 (s) | 7.56 (s) | 7.56 (s) | 7.56 (s) |
| 13 | 1.42 (s) | 1.32 (s) | 1.40 (s) | 1.28 (s) | 1.48 (s) | 1.51 (s) |
| 15 | 3.88 (s) | 3.84 (s) | 3.73 (s) | 3.79 (s) | 3.71 (s) | 3.71 (s) |
| 1′ |  |  |  | 5.52 (brd, 3.7) | 5.52 (brd, 3.8) | 5.50 (brd, 2.8) |
| 2′ |  |  |  | 2.07 (dd, 12.5, 3.8)  1.97 (dd, 12.7, 4.8) | 2.06 (dd, 12.8, 3.9)  1.91 (dd, 12.6, 4.7) | 2.24 (ddd, 13.1, 4.1, 1.4)  1.85 (td, 12.5, 3.9) |
| 3′ |  |  |  | 3.59 (m) | 3.45 (ddd, 12.7, 4.6, 2.8) | 3.22 (m) |
| 3′-N(CH_3_)_2_ |  |  |  |  |  |  |
| 4′ |  |  |  | 3.66 (brd, 2.9) | 3.67 (brd, 2.7) | 3.18 (t, 9.4) |
| 5′ |  |  |  | 4.17 (q, 6.1) | 4.16 (q, 6.3) | 3.92 (dq, 8.4, 6.2) |
| 6′ |  |  |  | 1.30 (d, 6.6) | 1.31 (d, 6.6) | 1.34 (d, 6.2) |

a) CDCl_3_; b) CD_3_OD; See Supplementary Information for NMR spectra. Assignments supported by 2D HSQC and HMBC experiments.

**Table S10.** ^1^H (600 MHz) NMR Spectroscopic Data of **24, 26**, **29**-**31** and **37** (*δ* in ppm).

| Position | **24** ^a)^ | **26** ^a)^ | **29** ^a)^ | **30** ^a)^ | **31** ^a)^ | **37** ^a)^ |
| --- | --- | --- | --- | --- | --- | --- |
|  | *δ*_H_ (mult, *J* in [Hz]) | *δ*_H_ (mult, *J* in [Hz]) | *δ*_H_ (mult, *J* in [Hz]) | *δ*_H_ (mult, *J* in [Hz]) | *δ*_H_ (mult, *J* in [Hz]) | *δ*_H_ (mult, *J* in [Hz]) |
| 1 | 7.76 (dd, 7.4, 1.4) | 7.74 (dd, 7.2, 2.0) |  |  |  |  |
| 2 | 7.73 (t, 7.6) | 7.72 (t, 7.8) | 7.37 (d, 9.4) | 7.33 (d, 9.4) | 7.39 (d, 9.4) | 7.36 (d, 9.6) |
| 3 | 7.31 (dd, 8.2, 1.4) | 7.30 (dd, 7.4, 2.1) | 7.35 (d, 9.4) | 7.31 (d, 9.3) | 7.37 (d, 9.3) | 7.34 (d, 9.6) |
| 4-OH |  |  |  |  |  |  |
| 6-OH |  |  |  |  |  |  |
| 7 | 5.06 (t, 7.8) | 5.16 (dd, 5.7, 2.1) | 5.17 (dd, 5.7, 2.1) | 5.17 (dd, 5.7, 2.0) | 5.19 (brdd, 5.7, 2.2) | 5.07 (t, 7.8) |
| 8 | 2.50 (dd, 13.6, 7.9)  2.40 (ddd, 13.6, 7.6, 1.9) | 2.77 (dd, 14.3, 5.8)  2.10 (brd, 14.2) | 2.78 (dd, 14.3, 5.7)  2.12 (dt, 14.4, 1.7) | 2.80 (dd, 14.3, 5.7)  2.14 (dt, 14.3, 1.7) | 2.77 (dd, 14.4, 5.7)  2.11 (brd, 14.3) | 2.50 (dd, 13.6, 8.0)  2.38 (ddd, 13.7, 7.6, 1.7) |
| 10 | 3.91 (brs) | 4.06 (brs) | 4.08 (s) | 4.09 (s) | 4.01 (s) | 3.93 (s) |
| 11 | 7.55 (s) | 7.52 (s) | 7.64 (s) | 7.62 (s) | 7.65 (s) | 7.65 (s) |
| 13 | 1.39 (s) | 1.50 (s) | 1.49 (s) | 1.52 (s) | 1.50 (s) | 1.40 (s) |
| 15 | 3.79 (s) | 3.72 (s) | 3.72 (s) | 3.73 (s) | 3.72 (s) | 3.79 (s) |
| 1′ | 5.60 (brdd, 4.0, 1.4) | 5.57 (dd, 4.0, 1.4) | 5.52 (brd, 3.7) | 5.50 (brd, 3.4) | 5.58 (brd, 3.8) | 5.60 (brd, 3.8) |
| 2′ | 2.28 (brdd, 12.5, 4.6)  2.08 (brdd, 12.7, 4.0) | 2.22 (dd, 12.6, 4.7)  2.06 (td, 12.9, 4.2) | 2.06 (dd, 12.8, 3.9)  1.91 (dd, 13.1, 4.2) | 2.25 (dd, 12.7, 3.8)  1.85 (m) | 2.21 (dd, 12.6, 4.5)  2.06 (td, 12.8, 4.1) | 2.28 (brdd, 12.2, 4.6)  2.07 (m) |
| 3′ | 3.49 (ddd, 12.9, 4.7, 2.9) | 3.35 (m) | 3.45 (dt, 12.7, 3.8) | 3.21 (m) | 3.33 (m) | 3.48 (dt, 12.8, 3.8) |
| 3′-N(CH_3_)_2_ | 2.92 (s)  2.85 (s) | 2.91 (s)  2.81 (s) |  |  | 2.91 (s)  2.81 (s) | 2.92 (s)  2.84 (s) |
| 4′ | 3.96 (brs) | 3.95 (brs) | 3.67 (brd, 2.4) | 3.19 (t, 9.8) | 3.96 (brd, 2.8) | 3.96 (brd, 2.7) |
| 5′ | 4.11 (brq, 6.5) | 4.14 (q, 6.5) | 4.16 (q, 6.1) | 3.93 (dq, 8.3, 6.3) | 4.14 (q, 6.7) | 4.12 (q, 6.6) |
| 6′ | 1.32 (d, 6.7) | 1.33 (d, 6.5) | 1.31 (d, 6.5) | 1.35 (d, 6.2) | 1.33 (d, 6.5) | 1.32 (d, 6.3) |

a) CD_3_OD; See Supplementary Information for NMR spectra. Assignments supported by 2D HSQC and HMBC experiments

**Table S11.** ^1^H (600 MHz) NMR Spectroscopic Data of **20**, **22** and **25** (*δ* in ppm).

| Position | **20** ^a)^ | **22** ^a)^ | **25** ^a)^ |
| --- | --- | --- | --- |
|  | *δ*_H_ (mult, *J* in [Hz]) | *δ*_H_ (mult, *J* in [Hz]) | *δ*_H_ (mult, *J* in [Hz]) |
| 1 | 7.81 (dd, 7.5, 1.4) | 7.74 (dd, 7.2, 2.0) | 7.71 (dd, 7.8, 2.6) |
| 2 | 7.77 (t, 7.8) | 7.72 (t, 7.5) | 7.77 (t, 7.5) |
| 3 | 7.35 (dd, 8.2, 1.4) | 7.30 (dd, 7.5, 2.0) | 7.28 (brdd, 6.7, 2.9) |
| 7 | 5.17 (dd, 6.1, 2.6) | 5.16 (dd, 6.1, 2.5) | 5.16 (dd, 6.2, 2.8) |
| 8 | 2.70 (dd, 14.3, 6.1)  2.13 (brdd, 14.4, 1.5) | 2.72 (dd, 14.3, 6.1)  2.15 (ddd, 14.4, 2.6, 1.2) | 2.72 (dd, 14.3, 6.1)  2.12 (ddd, 14.3, 2.9, 1.2) |
| 10 | 4.16 (s) | 4.17 (s) | 4.16 (s) |
| 11 | 7.55 (s) | 7.52 (s) | 7.50 (s) |
| 13 | 1.74-1.79 (m) | 1.83-1.77 (m) | 1.81 (dq,14.8, 7.4)  1.74 (dq, 14.6, 7.3) |
| 14 | 1.02 (t, 7.4) | 1.04 (t, 7.4) | 1.04 (t, 7.4) |
| 16 | 3.72 (s) | 3.74 (s) | 3.75 (s) |
| 1′ | 5.54 (brd, 3.7) | 5.50 (brd, 3.9) | 5.57 (brd, 2.6) |
| 2′ | 2.06 (brd, 3.9)  1.94 (dd, 12.5, 4.9) | 2.27 (ddd, 13.0, 4.4, 1.4)  1.86 (dd,12.7, 3.9) | 2.27 (m)  2.06 (td, 12.7, 4.0) |
| 2′-OCH_3_ |  |  |  |
| 3′ | 3.48 (m) | 3.24 (ddd, 12.3, 10.0, 4.3) | 3.36 (dt, 12.8, 4.3) |
| 3′-CH_3_ |  |  |  |
| 3′-N(CH_3_)_2_ |  |  | 2.92 (brs)  2.84 (brs) |
| 3′-NCOCH_3_ |  |  |  |
| 4′ | 3.66 (brd, 2.8) | 3.18 (t, 9.0) | 3.96 (brd, 2.9) |
| 5′ | 4.15 (q, 6.6) | 3.91 (dq, 9.0, 6.2) | 4.12 (q, 6.6) |
| 6′ | 1.30 (d, 6.6) | 1.34 (d, 6.2) | 1.32 (d, 6.6) |

a) CD_3_OD; See Supplementary Information for NMR spectra. Assignments supported by 2D HSQC and HMBC experiments.

**Table S12.** ^1^H (600 MHz) NMR Spectroscopic Data of **27**-**28 and 38**-**39** (*δ* in ppm).

| Position | **27** ^a)^ | **28** ^a)^ | **38** ^a)^ | **39** ^a)^ |
| --- | --- | --- | --- | --- |
|  | *δ*_H_ (mult, *J* in [Hz]) | *δ*_H_ (mult, *J* in [Hz]) | *δ*_H_ (mult, *J* in [Hz]) | *δ*_H_ (mult, *J* in [Hz]) |
| 1 | 7.78 (dd, 7.5, 1.0) | 7.76 (dd, 7.5, 1.0) | 7.75 (dd, 7.5, 1.6) | 7.80 (dd, 7.4, 1.4) |
| 2 | 7.76 (dd, 7.5, 8.4) | 7.73 (dd, 7.5, 8.4) | 7.72 (t, 7.6) | 7.76 (dd, 8.2, 7.5) |
| 3 | 7.34 (dd, 8.4, 1.0) | 7.31 (dd, 8.4, 1.0) | 7.29 (dd, 8.1, 1.5) | 7.34 (dd, 8.2, 1.3) |
| 7 | 5.13 (dd, 5.1, 1.8) | 4.91 (dd, 7.7, 7.3) | 5.13 (brdd, 6.1, 2.5) | 5.03 (t, 8.3) |
| 8 | 2.51 (dd, 14.9, 5.1)  2.33 (dd, 14.9, 1.8) | 2.65 (dd, 13.4, 7.7)  2.34 (dd, 13.4, 7.3) | 2.69 (dd, 14.3, 6.1)  2.15 (brdd, 14.2, 1.5) | 2.48 (brdd, 8.5 |
| 10 | 4.08 (s) | 3.96 (s) | 4.17 (s) | 3.94 (s) |
| 11 | 7.62 (s) | 7.52 (s) | 7.51 (s) | 7.61 (s) |
| 13 | 1.71 (dd, 14.0, 7.4)  1.53 (dd, 14.0, 7.4) | 1.49 (dd, 14.6, 7.4)  1.47 (dd, 14.6, 7.4) | 1.75-1.85 (m) | 1.71 (m)  1.61 (m) |
| 14 | 1.08 (t, 7.4) | 0.98 (t, 7.4) | 1.05 (t, 7.4) | 1.05 (t, 7.5) |
| 16 | 3.68 (s) | 3.76 (s) | 3.73 (s) | 3.76 (s) |
| 1′ | 5.41 (d, 1.5) | 5.41 (d, 1.3) | 5.47 (brd, 3.9) | 5.50 (brd, 3.6) |
| 2′ | 3.10 (d, 1.5) | 3.19 (d, 1.3) | 1.98 (brdd, 13.0, 4.0)  1.70 (dd, 13.1, 4.7) | 2.34 (ddd, 12.8, 4.6, 1.3)  2.06 (td, 12.8, 3.9) |
| 2′-OCH_3_ | 3.53 (s) | 3.55 (s) |  |  |
| 3′ |  |  | 4.13 (ddd, 13.1, 4.7, 2.8) | 3.36 (m) |
| 3′-CH_3_ | 1.15 (s) | 1.26 (s) |  |  |
| 3′-N(CH_3_)_2_ |  |  |  |  |
| 3′-NCOCH_3_ |  |  | 1.92 (s) |  |
| 4′ | 3.38 (d, 9.6) | 3.38 (d, 9.7) | 3.58 (brd, 2.5) | 3.17 (t, 9.5) |
| 5′ | 3.86 (dd, 9.6, 6.2) | 3.80 (dd, 9.7, 6.2) | 4.19 (brq, 6.0) | 3.88 (m) |
| 6′ | 1.30 (d, 6.2) | 1.30 (d, 6.2) | 1.27 (d, 6.7) | 1.33 (brd, 6.2) |

a) CD_3_OD; See Supplementary Information for NMR spectra. Assignments supported by 2D HSQC and HMBC experiments.

**Table S13.** Calculated physicochemical properties of the anthracyclines.

|  | **Chemaxon Marvin** | | | | | **SwissADME** | | | | |
| --- | --- | --- | --- | --- | --- | --- | --- | --- | --- | --- |
| **Compound** | **logP** | **Strongest acidic pKa** | **Strongest basic pKa** | **logS** | **charge state at pH=7.4** | **XLOGP3** | **WLOGP** | **MLOGP** | **Silicos-IT Log P** | **Consensus Log P** |
| **19** | 0.44 | 8.58 | 9.76 | -3.61 | +1 | 1.55 | 0.84 | -0.8 | 1.36 | 0.74 |
| **20** | 0.96 | 8.58 | 9.76 | -3.93 | +1 | 2.08 | 1.23 | -0.6 | 1.76 | 1.12 |
| **21** | 0.44 | 8.58 | 9.76 | -3.61 | +1 | 1.55 | 0.84 | -0.8 | 1.36 | 0.74 |
| **22** | 0.96 | 8.58 | 9.76 | -3.93 | +1 | 2.08 | 1.23 | -0.6 | 1.76 | 1.12 |
| **23** | 0.44 | 8.58 | 9.76 | -3.61 | +1 | 1.55 | 0.84 | -0.8 | 1.36 | 0.74 |
| **24** | 1.47 | 8.48 | 9.02 | -3.37 | +1 | 3.06 | 1.83 | -0.22 | 2.21 | 1.72 |
| **25** | 1.99 | 8.48 | 9.02 | -3.69 | +1 | 2.53 | 1.44 | -0.41 | 1.8 | 1.34 |
| **26** | 1.47 | 8.48 | 9.02 | -3.37 | +1 | 2.08 | 1.28 | -0.97 | 2.2 | 1.15 |
| **27** | 1.65 | 8.63 | -3.22 | -3.8 | 0 | 2.08 | 1.28 | -0.97 | 2.2 | 1.15 |
| **28** | 1.65 | 8.63 | -3.22 | -3.8 | 0 | 2.08 | 1.28 | -0.97 | 2.2 | 1.15 |
| **29** | 0.86 | 8.6 | 9.31 | -3.13 | +1 | 1.75 | 0.55 | -1.29 | 0.89 | 0.47 |
| **30** | 0.86 | 8.6 | 9.31 | -3.13 | +1 | 1.75 | 0.55 | -1.29 | 0.89 | 0.47 |
| **31** | 1.89 | 8.5 | 9.08 | -2.88 | +1 | 2.73 | 1.15 | -0.89 | 1.33 | 1.08 |
| **32** | 2.51 | 8.62 | 9.11 | -3.19 | +1 | 3.26 | 1.54 | -0.7 | 1.74 | 1.46 |
| **33** | 2.14 | 9.17 | 8.66 | -3.34 | +1 | 2.03 | 0.99 | -1.07 | 1.22 | 0.79 |
| **34** | 2.7 | 9.57 | 8.87 | -3.88 | +1 | 3.55 | 2.13 | -0.11 | 2.66 | 2.06 |
| **4** | 0.04 | 8.82 | 8.2 | -3 | +1 | 1.06 | 0.71 | -2.35 | 1.4 | 0.2 |
| **1** | 0.13 | 8.96 | 10.93 | -3.61 | +1 | 1.27 | -0.32 | -2.1 | 1.17 | 0.01 |
| **10** | 1.99 | 8.48 | 9.02 | -3.69 | +1 | 3.06 | 1.83 | -0.22 | 2.21 | 1.72 |
| **6** | 1.4 | 9.1 | -3.12 | -3.36 | 0 | 1.93 | 0.99 | -0.16 | 2.19 | 1.24 |
| **5** | 1.4 | 9.1 | -3.12 | -3.36 | 0 | 1.93 | 0.99 | -0.16 | 2.19 | 1.24 |
| **35** | 2.67 | 9.08 | -3.07 | -3.74 | 0 | 3.04 | 1.83 | 0.63 | 3.07 | 2.14 |
| **36** | 3.19 | 9.08 | -3.17 | -4.1 | 0 | 3.57 | 2.22 | 0.85 | 3.47 | 2.53 |
| **37** | 1.89 | 8.5 | 9.08 | -2.88 | +1 | 2.73 | 1.15 | -0.89 | 1.33 | 1.08 |
| **38** | 2.01 | 8.63 | 0.27 | -4.12 | +1 | 2.34 | 1.41 | -0.64 | 2.21 | 1.33 |
| **39** | 0.96 | 8.58 | 9.76 | -3.93 | +1 | 2.08 | 1.23 | -0.6 | 1.76 | 1.12 |

**Supplementary figures**

**Figure S1.** Functionality of GT and sugar modules.

**a**, Complementation of *snogE* and *snogN*. i, production of strain *S. albus* / pSnogaori∆snogN complemented with *snogN*; ii, production of strain *S. albus* / pSnogaori∆snogE complemented with *snogE*; iii, authentic standard of nogalamycin F (**9**)^[25]^; iv, production profile of *S. albus* / pSnogaori∆snogE strain^[25]^; v, production profile of *S. albus* / pSnogaori∆snogN strain. Traces of i-iii were detected at 470 nm; traces iv and v were recorded at 430 nm. **b**, Bioconversion experiments for verifying the functionality of GT *aknST* and TDP-L-rhodosamine genes (*desIII+IV*, *snogHIFGAX*). i, authentic standard aclacinomycin T (**10**); ii, feeding of **5** to strain AknST+RHO with GT *aknST* and TDP-L-rhodosamine genes; iii, production of strain AknST+RHO; iv, empty culture feeding with **5**; v, authentic standard of **5**. Traces were recorded at 430 nm. **c**, Bioconversion experiments for verifying the functionality of TDP-L-3’,4’-demethyl-nogalose sugar genes (*desIII+IV*, *snogG2FC*). i, co-elution of ii and iii; ii, feeding of **6** to strain SnogEN+DENOG with GT *snogEN* and TDP-L-3’,4’-demethyl-nogalose genes; iii, strain *S. albus* / pSnogaori∆snoaW producing **6** and L-3’,4’-demethyl-nogalose-nogalamycinone (**11**)^[48]^; iv, empty culture feeding with **6**; v, production of strain SnogEN+DENOG.

[M-H]^-^

**Figure S2.** (-)-ESI-MS spectra of compound **11** from cultural supernatant extracts

**Figure S3.** Comparison of compound **11** and **12** to authentic standards.

Authentic standard **11** was purified from *S. albus* / pSnogaori∆snoaW. Authentic standard **12** was chemically synthesized previously^[57]^.

[M-H]^-^

**Figure S4.** (-)-ESI-HR-MS spectra of compound **4** from cultural supernatant extracts

[M-H]^-^

**Figure S5.** (-)-ESI-MS spectra of compound **12** from cultural supernatant extracts

**Figure S6.** Stepwise optimization of the doxorubicin pathway refactoring.

**a**, Late steps of the doxorubicin biosynthetic pathway. **b–f**, Production of compounds **14–17** and **1** by the engineered strains RW-34, RW-36, RW-37, and RW-42. The values are presented as the ratio of HPLC peak area of each compound to the total peak area at 490 nm (mean ± SD, n = 3, biological triplicates).

[M-H]^-^

**Figure S7.** (-)-ESI-MS spectra of compound **14** from cultural supernatant extracts

[M+H]^+^

**Figure S8.** (+)-ESI-MS spectra of compound **15** from cultural supernatant extracts

[M-H]^-^

**Figure S9.** (-)-ESI-MS spectra of compound **16** from cultural supernatant extracts

[M-H]^-^

**Figure S10.** (-)-ESI-MS spectra of compound **1** from cultural supernatant extracts

[M-H]^-^

**Figure S11.** (-)-ESI-HR-MS spectra of compound **18** from cultural supernatant extracts

**Figure S12.** Chemical structures of compounds **6**, **19**-**31** and **35**-**39**.

**Figure S13.** ^1^H,^1^H-COSY (▬) and selected HMBC (→) correlations of compounds **19**-**31** and **36**-**39***.*

**Figure S14.** TOCSY (▬), and selected NOESY () correlations of compounds **19**-**31** and **38**-**39**.

NOESY/TOCSY spectra of **37** were not recorded due to limited amounts.

[(M-H_2_O)+H]^+^

[M-H]^-^

[(M-2H_2_O)+H]^+^

**Figure S15**. HPLC-MS analysis of nogalamycinone (**6**).

HPLC-conditions: solvent A: H_2_O/0.1% FA; solvent B: CH_3_CN; flow rate: 0.5 mL min^-1^; 0-30 min, 5-100% B; 30-35 min, 100% B; 35-36 min, 100-5% B; 36-40 min, 5% B; Phenomenex NX-C18 column (250 × 4.6 mm, 5 *μ*m); 254 nm, 280 nm, 400 nm. UV-vis inset of full wavelength scan (190-600 nm).

[M-H]^-^

**Figure S16.** (‒)**-**HRESI-MS spectrum of nogalamycinone (**6**).

**Figure S17.** ^1^H NMR spectrum (CDCl_3_, 600 MHz) of nogalamycinone (**6**).

**Figure S18.** ^13^C NMR spectrum (CDCl_3_, 150 MHz) of nogalamycinone (**6**).

**Figure S19.** ^1^H (CDCl_3_, 600 MHz) and ^13^C (CDCl_3_, 150 MHz) NMR spectra of nogalamycinone (**6**).

[M-H]^-^

[M+H]^+^

**Figure S20**: HPLC-MS analysis of 7-*O*-*α*-l-daunosaminyl-auramycinone (**19**).

HPLC-conditions: solvent A: H_2_O/0.1% FA; solvent B: CH_3_CN; flow rate: 0.5 mL min^-1^; 0-30 min, 5-100% B; 30-35 min, 100% B; 35-36 min, 100-5% B; 36-40 min, 5% B; Phenomenex NX-C18 column (250 × 4.6 mm, 5 *μ*m); 254 nm, 280 nm, 400 nm. UV-vis inset of full wavelength scan (190-600 nm).

[M-H]^-^

**Figure S21.** (‒)**-**HRESI-MS spectrum of 7-*O*-*α*-l-daunosaminyl-auramycinone (**19**).

**Figure S22.** ^1^H NMR spectrum (CD_3_OD, 600 MHz) of 7-*O*-*α*-l-daunosaminyl-auramycinone (**19**).

[Note: The sample of this compound contains trace contamination of diketopiperazine analog, cyclo-(L-Pro-L-Phe-)]

**Figure S23.** ^13^C NMR spectrum (CD_3_OD, 150 MHz) of 7-*O*-*α*-l-daunosaminyl-auramycinone (**19**).

**Figure S24.** ^1^H (CD_3_OD, 600 MHz) and ^13^C (CD_3_OD, 150 MHz) NMR spectra of 7-*O*-*α*-l-daunosaminyl-auramycinone (**19**).

**Figure S25.** ^1^H,^1^H-COSY spectrum (CD_3_OD, 600 MHz) of 7-*O*-*α*-l-daunosaminyl-auramycinone (**19**).

**Figure S26.** HSQC spectrum (CD_3_OD, 600 MHz) of 7-*O*-*α*-l-daunosaminyl-auramycinone (**19**).

**Figure S27.** HMBC spectrum (CD_3_OD, 600 MHz) of 7-*O*-*α*-l-daunosaminyl-auramycinone (**19**).

**Figure S28.** TOCSY spectrum (CD_3_OD, 600 MHz) of 7-*O*-*α*-l-daunosaminyl-auramycinone (**19**).

**Figure S29.** NOESY spectrum (CD_3_OD, 600 MHz) of 7-*O*-*α*-l-daunosaminyl-auramycinone (**19**).

[2M+H]^+^

[M-H]^-^

[M+H]^+^

**Figure S30**: HPLC-MS analysis of 7-*O*-*α*-l-daunosaminyl-9-*epi*-aklavinone (**20**).

HPLC-conditions: solvent A: H_2_O/0.1% FA; solvent B: CH_3_CN; flow rate: 0.5 mL min^-1^; 0-30 min, 5-100% B; 30-35 min, 100% B; 35-36 min, 100-5% B; 36-40 min, 5% B; Phenomenex NX-C18 column (250 × 4.6 mm, 5 *μ*m); 210 nm, 254 nm, 280 nm, 320 nm. UV-vis inset of full wavelength scan (190-600 nm).

[M-H]^-^

**Figure S31.** (‒)**-**HRESI-MS spectrum of 7-*O*-*α*-l-daunosaminyl-9-*epi*-aklavinone (**20**).

**Figure S32.** ^1^H NMR spectrum (CD_3_OD, 600 MHz) of 7-*O*-*α*-l-daunosaminyl-9-*epi*-aklavinone (**20**).

**Figure S33.** ^13^C NMR spectrum (CD_3_OD, 150 MHz) of 7-*O*-*α*-l-daunosaminyl-9-*epi*-aklavinone (**20**).

**Figure S34.** ^1^H (CD_3_OD, 600 MHz) and ^13^C (CD_3_OD, 150 MHz) NMR spectra of 7-*O*-*α*-l-daunosaminyl-9-*epi*-aklavinone (**20**).

**Figure S35.** ^1^H,^1^H-COSY spectrum (CD_3_OD, 600 MHz) of 7-*O*-*α*-l-daunosaminyl-9-*epi*-aklavinone (**20**).

**Figure S36.** HSQC spectrum (CD_3_OD, 600 MHz) of 7-*O*-*α*-l-daunosaminyl-9-*epi*-aklavinone (**20**).

**Figure S37.** HMBC spectrum (CD_3_OD, 600 MHz) of 7-*O*-*α*-l-daunosaminyl-9-*epi*-aklavinone (**20**).

**Figure S38.** TOCSY spectrum (CD_3_OD, 600 MHz) of 7-*O*-*α*-l-daunosaminyl-9-*epi*-aklavinone (**20**).

**Figure S39.** NOESY spectrum (CD_3_OD, 600 MHz) of 7-*O*-*α*-l-daunosaminyl-9-*epi*-aklavinone (**20**).

[M+H]^+^

[M-H]^-^

**Figure S40**: HPLC-MS analysis of 7-*O*-*α*-l-daunosaminyl-nogalamycinone (**21**).

HPLC-conditions: solvent A: H_2_O/0.1% FA; solvent B: CH_3_CN; flow rate: 0.5 mL min^-1^; 0-30 min, 5-100% B; 30-35 min, 100% B; 35-36 min, 100-5% B; 36-40 min, 5% B; Phenomenex NX-C18 column (250 × 4.6 mm, 5 *μ*m); 254 nm, 280 nm, 400 nm. UV-vis inset of full wavelength scan (190-600 nm).

[M-H]^-^

**Figure S41.** (‒)**-**HRESI-MS spectrum of 7-*O*-*α*-l-daunosaminyl-nogalamycinone (**21**).

**Figure S42.** ^1^H NMR spectrum (CD_3_OD, 600 MHz) of 7-*O*-*α*-l-daunosaminyl-nogalamycinone (**21**).

**Figure S43.** ^13^C NMR spectrum (CD_3_OD, 150 MHz) of 7-*O*-*α*-l-daunosaminyl-nogalamycinone (**21**).

**Figure S44.** ^1^H (CD_3_OD, 600 MHz) and ^13^C (CD_3_OD, 150 MHz) NMR spectra of 7-*O*-*α*-l-daunosaminyl-nogalamycinone (**21**).

**Figure S45.** ^1^H,^1^H-COSY spectrum (CD_3_OD, 600 MHz) of 7-*O*-*α*-l-daunosaminyl-nogalamycinone (**21**).

**Figure S46.** HSQC spectrum (CD_3_OD, 600 MHz) of 7-*O*-*α*-l-daunosaminyl-nogalamycinone (**21**).

**Figure S47.** HMBC spectrum (CD_3_OD, 600 MHz) of 7-*O*-*α*-l-daunosaminyl-nogalamycinone (**21**).

**Figure S48.** TOCSY spectrum (CD_3_OD, 600 MHz) of 7-*O*-*α*-l-daunosaminyl-nogalamycinone (**21**).

**Figure S49.** NOESY spectrum (CD_3_OD, 600 MHz) of 7-*O*-*α*-l-daunosaminyl-nogalamycinone (**21**).

[2M+H]^+^

[M-H]^-^

[M+H]^+^

**Figure S50**: HPLC-MS analysis of 7-*O*-*α*-l-acosaminyl-9-*epi*-aklavinone (**22**).

HPLC-conditions: solvent A: H_2_O/0.1% FA; solvent B: CH_3_CN; flow rate: 0.5 mL min^-1^; 0-30 min, 5-100% B; 30-35 min, 100% B; 35-36 min, 100-5% B; 36-40 min, 5% B; Phenomenex NX-C18 column (250 × 4.6 mm, 5 *μ*m); 210 nm, 254 nm, 280 nm, 320 nm. UV-vis inset of full wavelength scan (190-600 nm).

[M-H]^-^

**Figure S51.** (‒)**-**HRESI-MS spectrum of 7-*O*-*α*-l-acosaminyl-9-*epi*-aklavinone (**22**).

**Figure S52.** ^1^H NMR spectrum (CD_3_OD, 600 MHz) of 7-*O*-*α*-l-acosaminyl-9-*epi*-aklavinone (**22**).

**Figure S53.** ^13^C NMR spectrum (CD_3_OD, 150 MHz) of 7-*O*-*α*-l-acosaminyl-9-*epi*-aklavinone (**22**).

**Figure S54.** ^1^H (CD_3_OD, 600 MHz) and ^13^C (CD_3_OD, 150 MHz) NMR spectra of 7-*O*-*α*-l-acosaminyl-9-*epi*-aklavinone (**22**).

**Figure S55.** ^1^H,^1^H-COSY spectrum (CD_3_OD, 600 MHz) of 7-*O*-*α*-l-acosaminyl-9-*epi*-aklavinone (**22**).

**Figure S56.** HSQC spectrum (CD_3_OD, 600 MHz) of 7-*O*-*α*-l-acosaminyl-9-*epi*-aklavinone (**22**).

**Figure S57.** HMBC spectrum (CD_3_OD, 600 MHz) of 7-*O*-*α*-l-acosaminyl-9-*epi*-aklavinone (**22**).

**Figure S58.** TOCSY spectrum (CD_3_OD, 600 MHz) of 7-*O*-*α*-l-acosaminyl-9-*epi*-aklavinone (**22**).

**Figure S59.** NOESY spectrum (CD_3_OD, 600 MHz) of 7-*O*-*α*-l-acosaminyl-9-*epi*-aklavinone (**22**).

[2M+H]^+^

[M-H]^-^

[M+H]^+^

**Figure S60**: HPLC-MS analysis of 7-*O*-*α*-l-acosaminyl-nogalamycinone (**23**).

HPLC-conditions: solvent A: H_2_O/0.1% FA; solvent B: CH_3_CN; flow rate: 0.5 mL min^-1^; 0-30 min, 5-100% B; 30-35 min, 100% B; 35-36 min, 100-5% B; 36-40 min, 5% B; Phenomenex NX-C18 column (250 × 4.6 mm, 5 *μ*m); 254 nm, 280 nm, 400 nm. UV-vis inset of full wavelength scan (190-600 nm).

[M-H]^-^

**Figure S61.** (‒)**-**HRESI-MS spectrum of 7-*O*-*α*-l-acosaminyl-nogalamycinone (**23**).

[2M+H]^+^

[M+H]^+^

**Figure S62.** (+)**-**HRESI-MS spectrum of 7-*O*-*α*-l-acosaminyl-nogalamycinone (**23**).

**Figure S63.** ^1^H NMR spectrum (CD_3_OD, 600 MHz) of 7-*O*-*α*-l-acosaminyl-nogalamycinone (**23**).

**Figure S64.** ^13^C NMR spectrum (CD_3_OD, 150 MHz) of 7-*O*-*α*-l-acosaminyl-nogalamycinone (**23**).

**Figure S65.** ^1^H (CD_3_OD, 600 MHz) and ^13^C (CD_3_OD, 150 MHz) NMR spectra of 7-*O*-*α*-l-acosaminyl-nogalamycinone (**23**).

**Figure S66.** ^1^H,^1^H-COSY spectrum (CD_3_OD, 600 MHz) of 7-*O*-*α*-l-acosaminyl-nogalamycinone (**23**).

**Figure S67.** HSQC spectrum (CD_3_OD, 600 MHz) of 7-*O*-*α*-l-acosaminyl-nogalamycinone (**23**).

**Figure S68.** HMBC spectrum (CD_3_OD, 600 MHz) of 7-*O*-*α*-l-acosaminyl-nogalamycinone (**23**).

**Figure S69.** TOCSY spectrum (CD_3_OD, 600 MHz) of 7-*O*-*α*-l-acosaminyl-nogalamycinone (**23**).

**Figure S70.** NOESY spectrum (CD_3_OD, 600 MHz) of 7-*O*-*α*-l-acosaminyl-nogalamycinone (**23**).

[M-H]^-^

[M+H]^+^

**Figure S71**: HPLC-MS analysis of 7-*O*-*α*-l-rhodosaminyl-9-*epi*-nogalamycinone (**24**).

HPLC-conditions: solvent A: H_2_O/0.1% FA; solvent B: CH_3_CN; flow rate: 0.5 mL min^-1^; 0-30 min, 5-100% B; 30-35 min, 100% B; 35-36 min, 100-5% B; 36-40 min, 5% B; Phenomenex NX-C18 column (250 × 4.6 mm, 5 *μ*m); 254 nm, 280 nm, 400 nm. UV-vis inset of full wavelength scan (190-600 nm).

[M-H]^-^

**Figure S72.** (‒)**-**HRESI-MS spectrum of 7-*O*-*α*-l-rhodosaminyl-9-*epi*-nogalamycinone (**24**).

**Figure S73.** ^1^H NMR spectrum (CD_3_OD, 600 MHz) of 7-*O*-*α*-l-rhodosaminyl-9-*epi*-nogalamycinone (**24**).

**Figure S74.** ^13^C NMR spectrum (CD_3_OD, 150 MHz) of 7-*O*-*α*-l-rhodosaminyl-9-*epi*-nogalamycinone (**24**).

**Figure S75.** ^1^H (CD_3_OD, 600 MHz) and ^13^C (CD_3_OD, 150 MHz) NMR spectra of 7-*O*-*α*-l-rhodosaminyl-9-*epi*-nogalamycinone (**24**).

**Figure S76.** ^1^H,^1^H-COSY spectrum (CD_3_OD, 600 MHz) of 7-*O*-*α*-l-rhodosaminyl-9-*epi*-nogalamycinone (**24**).

**Figure S77.** HSQC spectrum (CD_3_OD, 600 MHz) of 7-*O*-*α*-l-rhodosaminyl-9-*epi*-nogalamycinone (**24**).

**Figure S78.** HMBC spectrum (CD_3_OD, 600 MHz) of 7-*O*-*α*-l-rhodosaminyl-9-*epi*-nogalamycinone (**24**).

**Figure S80.** TOCSY spectrum (CD_3_OD, 600 MHz) of 7-*O*-*α*-l-rhodosaminyl-9-*epi*-nogalamycinone (**24**).

**Figure S81.** NOESY spectrum (CD_3_OD, 600 MHz) of 7-*O*-*α*-l-rhodosaminyl-9-*epi*-nogalamycinone (**24**).

[M-H]^-^

[M+H]^+^

**Figure S82**: HPLC-MS analysis of 7-*O*-*α*-l-rhodosaminyl-9-*epi*-aklavinone (**25**).

HPLC-conditions: solvent A: H_2_O/0.1% FA; solvent B: CH_3_CN; flow rate: 0.5 mL min^-1^; 0-30 min, 5-100% B; 30-35 min, 100% B; 35-36 min, 100-5% B; 36-40 min, 5% B; Phenomenex NX-C18 column (250 × 4.6 mm, 5 *μ*m); 210 nm, 254 nm, 280 nm, 400 nm. UV-vis inset of full wavelength scan (190-600 nm).

[M+H]^+^

**Figure S83.** (+)**-**HRESI-MS spectrum of 7-*O*-*α*-l-rhodosaminyl-9-*epi*-aklavinone (**25**).

**Figure S84.** ^1^H NMR spectrum (CD_3_OD, 600 MHz) of 7-*O*-*α*-l-rhodosaminyl-9-*epi*-aklavinone (**25**).

**Figure S85.** ^13^C NMR spectrum (CD_3_OD, 150 MHz) of 7-*O*-*α*-l-rhodosaminyl-9-*epi*-aklavinone (**25**).

**Figure S86.** ^1^H (CD_3_OD, 600 MHz) and ^13^C (CD_3_OD, 150 MHz) NMR spectra of 7-*O*-*α*-l-rhodosaminyl-9-*epi*-aklavinone (**25**).

**Figure S87.** ^1^H,^1^H-COSY spectrum (CD_3_OD, 600 MHz) of 7-*O*-*α*-l-rhodosaminyl-9-*epi*-aklavinone (**25**).

**Figure S88.** HSQC spectrum (CD_3_OD, 600 MHz) of 7-*O*-*α*-l-rhodosaminyl-9-*epi*-aklavinone (**25**).

**Figure S89.** HMBC spectrum (CD_3_OD, 600 MHz) of 7-*O*-*α*-l-rhodosaminyl-9-*epi*-aklavinone (**25**).

**Figure S90.** TOCSY spectrum (CD_3_OD, 600 MHz) of 7-*O*-*α*-l-rhodosaminyl-9-*epi*-aklavinone (**25**).

**Figure S91.** NOESY spectrum (CD_3_OD, 600 MHz) of 7-*O*-*α*-l-rhodosaminyl-9-*epi*-aklavinone (**25**).

[M-H]^-^

[M+H]^+^

**Figure S92**: HPLC-MS analysis of 7-*O*-*α*-l-rhodosaminyl-nogalamycinone (**26**). HPLC-conditions: solvent A: H_2_O/0.1% FA; solvent B: CH_3_CN; flow rate: 0.5 mL min^-1^; 0-30 min, 5-100% B; 30-35 min, 100% B; 35-36 min, 100-5% B; 36-40 min, 5% B; Phenomenex NX-C18 column (250 × 4.6 mm, 5 *μ*m); 254 nm, 280 nm, 400 nm. UV-vis inset of full wavelength scan (190-600 nm).

[M-H]^-^

**Figure S93.** (‒)**-**HRESI-MS spectrum of 7-*O*-*α*-l-rhodosaminyl-nogalamycinone (**26**).

**Figure S94.** ^1^H NMR spectrum (CD_3_OD, 600 MHz) of 7-*O*-*α*-l-rhodosaminyl-nogalamycinone (**26**).

**Figure S95.** ^13^C NMR spectrum (CD_3_OD, 150 MHz) of 7-*O*-*α*-l-rhodosaminyl-nogalamycinone (**26**).

**Figure S96.** ^1^H (CD_3_OD, 600 MHz) and ^13^C (CD_3_OD, 150 MHz) NMR spectra of 7-*O*-*α*-l-rhodosaminyl-nogalamycinone (**26**).

**Figure S97.** ^1^H,^1^H-COSY spectrum (CD_3_OD, 600 MHz) of 7-*O*-*α*-l-rhodosaminyl-nogalamycinone (**26**).

**Figure S98.** HSQC spectrum (CD_3_OD, 600 MHz) of 7-*O*-*α*-l-rhodosaminyl-nogalamycinone (**26**).

**Figure S99.** HMBC spectrum (CD_3_OD, 600 MHz) of 7-*O*-*α*-l-rhodosaminyl-nogalamycinone (**26**).

**Figure S100.** TOCSY spectrum (CD_3_OD, 600 MHz) of 7-*O*-*α*-l-rhodosaminyl-nogalamycinone (**26**).

**Figure S101.** NOESY spectrum (CD_3_OD, 600 MHz) of 7-*O*-*α*-l-rhodosaminyl-nogalamycinone (**26**).

[M+Na]^+^

[M+Na]^+^

**Figure S102.** (+)-HRESI-MS spectrum of 7-*O*-*α*-l-3′,4′-demethyl-nogalosyl-9-*epi*-akalvinone (**27**).

**Figure S103.** ^1^H NMR spectrum (CD_3_OD, 600 MHz) of 7-O-α-l-3′,4′-demethyl-nogalosyl-9-epi-akalvinone (**27**).

**Figure S104.** ^13^C NMR spectrum (CD_3_OD, 125 MHz) of 7-O-α-l-3′,4′-demethyl-nogalosyl-9-epi-akalvinone (**27**).

**Figure S105.** ^1^H,^1^H-COSY spectrum (CD_3_OD, 600 MHz) of 7-O-α-l-3′,4′-demethyl-nogalosyl-9-epi-akalvinone (**27**).

**Figure S106.** HSQC spectrum (CD_3_OD, 600 MHz) of 7-O-α-l-3′,4′-demethyl-nogalosyl-9-epi-akalvinone (**27**).

**Figure S107.** HMBC spectrum (CD_3_OD, 600 MHz) of 7-O-α-l-3′,4′-demethyl-nogalosyl-9-epi-akalvinone (**27**).

**Figure S108.** NOESY spectrum (CD_3_OD, 600 MHz) of 7-O-α-l-3′,4′-demethyl-nogalosyl-9-epi-akalvinone (**27**).

[M-H]^-^

[M-H]^-^

**Figure S109.** (-)-HRESI-MS spectrum of 7-O-α-l-3′,4′-demethyl-nogalosyl-akalvinone (**28**).

**Figure S110.** ^1^H NMR spectrum (CD_3_OD, 600 MHz) of 7-O-α-l-3′,4′-demethyl-nogalosyl-akalvinone (**28**).

**Figure S111.** ^13^C NMR spectrum (CD_3_OD, 150 MHz) of 7-O-α-l-3′,4′-demethyl-nogalosyl-akalvinone (**28**).

**Figure S112.** ^1^H,^1^H-COSY spectrum (CD_3_OD, 600 MHz) of 7-O-α-l-3′,4′-demethyl-nogalosyl-akalvinone (**28**).

**Figure S113.** HSQC spectrum (CD_3_OD, 600 MHz) of 7-O-α-l-3′,4′-demethyl-nogalosyl-akalvinone (**28**).

**Figure S114.** HMBC spectrum (CD_3_OD, 600 MHz) of 7-O-α-l-3′,4′-demethyl-nogalosyl-akalvinone (**28**).

**Figure S115.** NOESY spectrum (CD_3_OD, 600 MHz) of 7-O-α-l-3′,4′-demethyl-nogalosyl-akalvinone (**28**).

[M-H]^-^

[2M+H]^+^

[M+H]^+^

**Figure S116**: HPLC-MS analysis of 1-hydroxy-7-*O*-*α*-l-daunosaminyl-nogalamycinone (**29**). HPLC-conditions: solvent A: H_2_O/0.1% FA; solvent B: CH_3_CN; flow rate: 0.5 mL min^-1^; 0-30 min, 5-100% B; 30-35 min, 100% B; 35-36 min, 100-5% B; 36-40 min, 5% B; Phenomenex NX-C18 column (250 × 4.6 mm, 5 *μ*m); 254 nm, 280 nm, 400 nm. UV-vis inset of full wavelength scan (190-600 nm).

[M-H]^-^

**Figure S117.** (‒)**-**HRESI-MS spectrum of 1-hydroxy-7-*O*-*α*-l-daunosaminyl-nogalamycinone (**29**).

**Figure S118.** ^1^H NMR spectrum (CD_3_OD, 600 MHz) of 1-hydroxy-7-*O*-*α*-l-daunosaminyl-nogalamycinone (**29**).

**Figure S119.** ^13^C NMR spectrum (CD_3_OD, 150 MHz) of 1-hydroxy-7-*O*-*α*-l-daunosaminyl-nogalamycinone (**29**).

**Figure S120.** ^1^H (CD_3_OD, 600 MHz) and ^13^C (CD_3_OD, 150 MHz) NMR spectra of 1-hydroxy-7-*O*-*α*-l-daunosaminyl-nogalamycinone (**29**).

**Figure S121.** ^1^H,^1^H-COSY spectrum (CD_3_OD, 600 MHz) of 1-hydroxy-7-*O*-*α*-l-daunosaminyl-nogalamycinone (**29**).

**Figure S122.** HSQC spectrum (CD_3_OD, 600 MHz) of 1-hydroxy-7-*O*-*α*-l-daunosaminyl-nogalamycinone (**29**).

**Figure S123.** HMBC spectrum (CD_3_OD, 600 MHz) of 1-hydroxy-7-*O*-*α*-l-daunosaminyl-nogalamycinone (**29**).

**Figure S124.** TOCSY spectrum (CD_3_OD, 600 MHz) of 1-hydroxy-7-*O*-*α*-l-daunosaminyl-nogalamycinone (**29**).

**Figure S125.** NOESY spectrum (CD_3_OD, 600 MHz) of 1-hydroxy-7-*O*-*α*-l-daunosaminyl-nogalamycinone (**29**).

[2M+H]^+^

[M-H]^-^

[M+H]^+^

**Figure S126**: HPLC-MS analysis of 1-hydroxy-7-*O*-*α*-l-acosaminyl-nogalamycinone (**30**). HPLC-conditions: solvent A: H_2_O/0.1% FA; solvent B: CH_3_CN; flow rate: 0.5 mL min^-1^; 0-30 min, 5-100% B; 30-35 min, 100% B; 35-36 min, 100-5% B; 36-40 min, 5% B; Phenomenex NX-C18 column (250 × 4.6 mm, 5 *μ*m); 254 nm, 280 nm, 400 nm. UV-vis inset of full wavelength scan (190-600 nm).

[M-H]^-^

**Figure S127.** (‒)**-**HRESI-MS spectrum of 1-hydroxy-7-*O*-*α*-l-acosaminyl-nogalamycinone (**30**).

**Figure S128.** ^1^H NMR spectrum (CD_3_OD, 600 MHz) of 1-hydroxy-7-*O*-*α*-l-acosaminyl-nogalamycinone (**30**).

**Figure S129.** ^13^C NMR spectrum (CD_3_OD, 150 MHz) of 1-hydroxy-7-*O*-*α*-l-acosaminyl-nogalamycinone (**30**).

**Figure S130.** ^1^H (CD_3_OD, 600 MHz) and ^13^C (CD_3_OD, 150 MHz) NMR spectra of 1-hydroxy-7-*O*-*α*-l-acosaminyl-nogalamycinone (**30**).

**Figure S131.** ^1^H,^1^H-COSY spectrum (CD_3_OD, 600 MHz) of 1-hydroxy-7-*O*-*α*-l-acosaminyl-nogalamycinone (**30**).

**Figure S132.** HSQC spectrum (CD_3_OD, 600 MHz) of 1-hydroxy-7-*O*-*α*-l-acosaminyl-nogalamycinone (**30**).

**Figure S133.** HMBC spectrum (CD_3_OD, 600 MHz) of 1-hydroxy-7-*O*-*α*-l-acosaminyl-nogalamycinone (**30**).

**Figure S134.** TOCSY spectrum (CD_3_OD, 600 MHz) of 1-hydroxy-7-*O*-*α*-l-acosaminyl-nogalamycinone (**30**).

**Figure S135.** NOESY spectrum (CD_3_OD, 600 MHz) of 1-hydroxy-7-*O*-*α*-l-acosaminyl-nogalamycinone (**30**).

[M-H]^-^

[M+H]^+^

**Figure S136**: HPLC-MS analysis of 1-hydroxy-7-*O*-*α*-l-rhodosaminyl-nogalamycinone (**31**). HPLC-conditions: solvent A: H_2_O/0.1% FA; solvent B: CH_3_CN; flow rate: 0.5 mL min^-1^; 0-30 min, 5-100% B; 30-35 min, 100% B; 35-36 min, 100-5% B; 36-40 min, 5% B; Phenomenex NX-C18 column (250 × 4.6 mm, 5 *μ*m); 254 nm, 280 nm. UV-vis inset of full wavelength scan (190-600 nm).

[M-H]^-^

**Figure S137.** (‒)**-**HRESI-MS spectrum of 1-hydroxy-7-*O*-*α*-l-rhodosaminyl-nogalamycinone (**31**).

**Figure S138.** ^1^H NMR spectrum (CD_3_OD, 600 MHz) of 1-hydroxy-7-*O*-*α*-l-rhodosaminyl-nogalamycinone (**31**).

**Figure S139.** ^13^C NMR spectrum (CD_3_OD, 150 MHz) of 1-hydroxy-7-*O*-*α*-l-rhodosaminyl-nogalamycinone (**31**).

**Figure S140.** ^1^H,^1^H-COSY spectrum (CD_3_OD, 600 MHz) of 1-hydroxy-7-*O*-*α*-l-rhodosaminyl-nogalamycinone (**31**).

**Figure S141.** HSQC spectrum (CD_3_OD, 600 MHz) of 1-hydroxy-7-*O*-*α*-l-rhodosaminyl-nogalamycinone (**31**).

**Figure S142.** HMBC spectrum (CD_3_OD, 600 MHz) of 1-hydroxy-7-*O*-*α*-l-rhodosaminyl-nogalamycinone (**31**).

**Figure S143.** TOCSY spectrum (CD_3_OD, 600 MHz) of 1-hydroxy-7-*O*-*α*-l-rhodosaminyl-nogalamycinone (**31**).

**Figure S144.** NOESY spectrum (CD_3_OD, 600 MHz) of 1-hydroxy-7-*O*-*α*-l-rhodosaminyl-nogalamycinone (**31**).

[(M-H_2_O)+H]^+^

[M+H]^+^

[M+Na]^+^

[M-H]^-^

**Figure S145**. HPLC-MS analysis of 7-deoxy-nogalamycinone (**35**). HPLC-conditions: solvent A: H_2_O/0.1% FA; solvent B: CH_3_CN; flow rate: 0.5 mL min^-1^; 0-30 min, 5-100% B; 30-35 min, 100% B; 35-36 min, 100-5% B; 36-40 min, 5% B; Phenomenex NX-C18 column (250 × 4.6 mm, 5 *μ*m); 254 nm, 280 nm, 400 nm. UV-vis inset of full wavelength scan (190-600 nm).

[M-H]^-^

**Figure S146.** (‒)**-**HRESI-MS spectrum of 7-deoxy-nogalamycinone (**35**).

**Figure S147.** ^1^H NMR spectrum (CDCl_3_, 600 MHz) of 7-deoxy-nogalamycinone (**35**).

**Figure S148.** ^13^C NMR spectrum (CDCl_3_, 150 MHz) of 7-deoxy-nogalamycinone (**35**).

**Figure S149.** ^1^H (CDCl_3_, 600 MHz) and ^13^C (CDCl_3_, 150 MHz) NMR spectra of 7-deoxy-nogalamycinone (**35**).

[M-H]^-^

[M+H]^+^

**Figure S150**. HPLC-MS analysis of 7-deoxy-9-*epi*-nogalamycinone (7-deoxy-auramycinone; **36**). HPLC-conditions: solvent A: H_2_O/0.1% FA; solvent B: CH_3_CN; flow rate: 0.5 mL min^-1^; 0-30 min, 5-100% B; 30-35 min, 100% B; 35-36 min, 100-5% B; 36-40 min, 5% B; Phenomenex NX-C18 column (250 × 4.6 mm, 5 *μ*m); 254 nm, 280 nm, 400 nm. UV-vis inset of full wavelength scan (190-600 nm).

[M-H]^-^

**Figure S151.** (‒)**-**HRESI-MS spectrum of 7-deoxy-9-*epi*-nogalamycinone (7-deoxy-auramycinone; **36**).

**Figure S152.** ^1^H NMR spectrum (CDCl_3_, 600 MHz) of 7-deoxy-9-*epi*-nogalamycinone (7-deoxy-auramycinone; **36**).

**Figure S153.** ^13^C NMR spectrum (CDCl_3_, 150 MHz) of 7-deoxy-9-*epi*-nogalamycinone (7-deoxy-auramycinone; **36**).

**Figure S154.** ^1^H (CDCl_3_, 600 MHz) and ^13^C (CDCl_3_, 150 MHz) NMR spectra of 7-deoxy-9-*epi*-nogalamycinone (7-deoxy-auramycinone; **36**).

**Figure S155.** ^1^H,^1^H-COSY spectrum (CDCl_3_, 600 MHz) of 7-deoxy-9-*epi*-nogalamycinone (7-deoxy-auramycinone; **36**).

**Figure S156.** HSQC spectrum (CDCl_3_, 600 MHz) of 7-deoxy-9-*epi*-nogalamycinone (7-deoxy-auramycinone; **36**).

**Figure S157.** HMBC spectrum (CDCl_3_, 600 MHz) of 7-deoxy-9-*epi*-nogalamycinone (7-deoxy-auramycinone; **36**).

[M-H]^-^

[M+H]^+^

**Figure S158**. HPLC-MS analysis of 1-hydroxy-7-*O*-*α*-l-rhodosaminyl-9-*epi*-nogalamycinone (**37**). HPLC-conditions: solvent A: H_2_O/0.1% FA; solvent B: CH_3_CN; flow rate: 0.5 mL min^-1^; 0-30 min, 5-100% B; 30-35 min, 100% B; 35-36 min, 100-5% B; 36-40 min, 5% B; Phenomenex NX-C18 column (250 × 4.6 mm, 5 *μ*m); 254 nm, 280 nm. UV-vis inset of full wavelength scan (190-600 nm).

[M-H]^-^

**Figure S159.** (‒)**-**HRESI-MS spectrum of 1-hydroxy-7-*O*-*α*-l-rhodosaminyl-9-*epi*-nogalamycinone (**37**).

**Figure S160.** ^1^H NMR spectrum (CD_3_OD, 600 MHz) of 1-hydroxy-7-*O*-*α*-l-rhodosaminyl-9-*epi*-nogalamycinone (**37**).

**Figure S161.** ^13^C NMR spectrum (CD_3_OD, 150 MHz) of 1-hydroxy-7-*O*-*α*-l-rhodosaminyl-9-*epi*-nogalamycinone (**37**).

**Figure S162.** ^1^H (CD_3_OD, 600 MHz) and ^13^C (CD_3_OD, 150 MHz) NMR spectra of 1-hydroxy-7-*O*-*α*-l-rhodosaminyl-9-*epi*-nogalamycinone (**37**).

**Figure S163.** ^1^H,^1^H-COSY spectrum (CD_3_OD, 600 MHz) of 1-hydroxy-7-*O*-*α*-l-rhodosaminyl-9-*epi*-nogalamycinone (**37**).

**Figure S164.** HSQC spectrum (CD_3_OD, 600 MHz) of 1-hydroxy-7-*O*-*α*-l-rhodosaminyl-9-*epi*-nogalamycinone (**37**).

**Figure S165.** HMBC spectrum (CD_3_OD, 600 MHz) of 1-hydroxy-7-*O*-*α*-l-rhodosaminyl-9-*epi*-nogalamycinone (**37**).

[(M-sugar/-2H_2_O)+H]^+^

[M-H]^-^

**Figure S166**. HPLC-MS analysis of *N*-acetyl-7-*O*-*α*-l-daunosaminyl-9-*epi*-aklavinone (**38**).HPLC-conditions: solvent A: H_2_O/0.1% FA; solvent B: CH_3_CN; flow rate: 0.5 mL min^-1^; 0-30 min, 5-100% B; 30-35 min, 100% B; 35-36 min, 100-5% B; 36-40 min, 5% B; Phenomenex NX-C18 column (250 × 4.6 mm, 5 *μ*m); 210 nm, 254 nm, 280 nm, 320 nm. UV-vis inset of full wavelength scan (190-600 nm).

[M-H]^-^

**Figure S167.** (‒)**-**HRESI-MS spectrum of *N*-acetyl-7-*O*-*α*-l-daunosaminyl-9-*epi*-aklavinone (**38**).

**Figure S168.** ^1^H NMR spectrum (CD_3_OD, 600 MHz) of *N*-acetyl-7-*O*-*α*-l-daunosaminyl-9-*epi*-aklavinone (**38**).

**Figure S169.** ^13^C NMR spectrum (CD_3_OD, 150 MHz) of *N*-acetyl-7-*O*-*α*-l-daunosaminyl-9-*epi*-aklavinone (**38**).

**Figure S170.** ^1^H (CD_3_OD, 600 MHz) and ^13^C (CD_3_OD, 150 MHz) NMR spectra of *N*-acetyl-7-*O*-*α*-l-daunosaminyl-9-*epi*-aklavinone (**38**).

**Figure S171.** ^1^H,^1^H-COSY spectrum (CD_3_OD, 600 MHz) of *N*-acetyl-7-*O*-*α*-l-daunosaminyl-9-*epi*-aklavinone (**38**).

**Figure S172.** HSQC spectrum (CD_3_OD, 600 MHz) of *N*-acetyl-7-*O*-*α*-l-daunosaminyl-9-*epi*-aklavinone (**38**).

**Figure S173.** HMBC spectrum (CD_3_OD, 600 MHz) of *N*-acetyl-7-*O*-*α*-l-daunosaminyl-9-*epi*-aklavinone (**38**).

**Figure S174.** TOCSY spectrum (CD_3_OD, 600 MHz) of *N*-acetyl-7-*O*-*α*-l-daunosaminyl-9-*epi*-aklavinone (**38**).

**Figure S175.** NOESY spectrum (CD_3_OD, 600 MHz) of *N*-acetyl-7-*O*-*α*-l-daunosaminyl-9-*epi*-aklavinone (**38**).

[2M+H]^+^

[M-H]^-^

[M+H]^+^

**Figure S176**. HPLC-MS analysis of 7-*O*-*α*-l-acosaminyl-aklavinone (**39**). HPLC-conditions: solvent A: H_2_O/0.1% FA; solvent B: CH_3_CN; flow rate: 0.5 mL min^-1^; 0-30 min, 5-100% B; 30-35 min, 100% B; 35-36 min, 100-5% B; 36-40 min, 5% B; Phenomenex NX-C18 column (250 × 4.6 mm, 5 *μ*m); 210 nm, 254 nm, 280 nm, 320 nm. UV-vis inset of full wavelength scan (190-600 nm).

[M-H]^-^

**Figure S177.** (‒)**-**HRESI-MS spectrum of 7-*O*-*α*-l-acosaminyl-aklavinone (**39**).

**Figure S178.** ^1^H NMR spectrum (CD_3_OD, 600 MHz) of 7-*O*-*α*-l-acosaminyl-aklavinone (**39**).

**Figure S179.** ^13^C NMR spectrum (CD_3_OD, 150 MHz) of 7-*O*-*α*-l-acosaminyl-aklavinone (**39**).

**Figure S180.** ^1^H (CD_3_OD, 600 MHz) and ^13^C (CD_3_OD, 150 MHz) NMR spectra of 7-*O*-*α*-l-acosaminyl-aklavinone (**39**).

**Figure S181.** ^1^H,^1^H-COSY spectrum (CD_3_OD, 600 MHz) of 7-*O*-*α*-l-acosaminyl-aklavinone (**39**).

**Figure S182.** HSQC spectrum (CD_3_OD, 600 MHz) of 7-*O*-*α*-l-acosaminyl-aklavinone (**39**).

**Figure S183.** HMBC spectrum (CD_3_OD, 600 MHz) of 7-*O*-*α*-l-acosaminyl-aklavinone (**39**).

**Figure S184.** TOCSY spectrum (CD_3_OD, 600 MHz) of 7-*O*-*α*-l-acosaminyl-aklavinone (**39**).

**Figure S185.** NOESY spectrum (CD_3_OD, 600 MHz) of 7-*O*-*α*-l-acosaminyl-aklavinone (**39**).

[M+H]^+^

**Figure S186.** (+)-ESI-MS spectra of compound **32** from cultural supernatant extracts

[M+H]^+^

**Figure S187.** (+)-ESI-MS spectra of compound **33** from cultural supernatant extracts

[M+H]^+^

**Figure S188.** (+)-ESI-MS spectra of compound **34** from cultural supernatant extracts

**Figure S189.** Comparison of compound **32**, **33**, and **34** to authentic or enzymatic standards. Standards for **32** and **33** were enzymatically synthesized according to the method described in the method section. Authentic standard for **34** were synthesized previously^[57]^.

**Figure S190**. Viability of 5 human cancer cell lines at 80 µM of anthracycline compounds.

A549 (non-small lung), PC3 (prostate) and HCT116 (colorectal) human cancer cell lines and Merkel cells MKL1 and MCC26 (after 72h) at 80 mM concentrations of compounds **4**-**6**, **19**-**24**, **26**-**31** and **35**-**39**.

**Figure S191.** Viability of 5 human cancer cell lines at 10 µM of anthracycline compounds.

A549 (non-small lung), PC3 (prostate) and HCT116 (colorectal) human cancer cell lines and MKL1 and MCC26 (Merkel cells) after 72h exposed to 10 μM concentrations of the indicated compounds. Samples are normalized to DMSO treated controls.

**a**

**b**

**d**

**c**

**e**

**Figure S192.** Dose-response of compounds **1**, **4**, **19**-**26**, **29**-**31** and **37**-**38** against cancer cell lines. **a**, A549 (non-small cell lung); **b**, PC3 (prostate); **c**, HCT116 (colorectal); **d**, MCC26 ); **e**, MKL1 cancer cell lines.

**Figure S193.** Concentration–response curves of anthracyclines in the DNA dye competition assays. **a**, PicoGreen; **b**, Hoechst 33258. Relative fluorescence was calculated after subtraction of the fluorescence background from anthracyclines and normalized by the fluorescence measured in the absence of anthracycline (0 μM). Curves were fitted using nonlinear regression (n = 3).

**Figure S194.** Uncropped Western blot gels indicating DNA damage activities of tested compounds.
